# Cytosponge-trefoil factor 3 versus usual care to identify Barrett's oesophagus in a primary care setting: a multicentre, pragmatic, randomised controlled trial

**DOI:** 10.1016/S0140-6736(20)31099-0

**Published:** 2020-08-01

**Authors:** Rebecca C Fitzgerald, Massimiliano di Pietro, Maria O'Donovan, Roberta Maroni, Beth Muldrew, Irene Debiram-Beecham, Marcel Gehrung, Judith Offman, Monika Tripathi, Samuel G Smith, Benoit Aigret, Fiona M Walter, Greg Rubin, BEST3 Trial team, Abhay Bagewadi, Abigail Patrick, Achuth Shenoy, Aisling Redmond, Ajay Muddu, Alex Northrop, Alice Groves, Alice Shiner, Amardeep Heer, Amrit Takhar, Amy Bowles, Andrea Jarman, Angela Wong, Angie Lucas, Anita Gibbons, Anjan Dhar, Anji Curry, Anna Lalonde, Anna Swinburn, Anne Turner, Anne-Marie Lydon, Anthony Gunstone, Arlene Lee, Arul Nambi, Arun Ariyarathenam, Ashley Elden, Ashley Wilson, Balaji Donepudi, Barbara Campbell, Basia Uszycka, Ben Bowers, Ben Coghill, Bruno de Quadros, Calvin Cheah, Carla Bratten, Carly Brown, Chantelle Moorbey, Charles Clisby, Charles Gordon, Chris Schramm, Chris Castle, Chris Newark, Chrissie Norris, Christine A'Court, Claire Graham, Clare Fletcher, Clare Grocott, Colin Rees, Corinne Bakker, Costas Paschalides, Craig Vickery, Damian Schembri, Danielle Morris, Daryl Hagan, David Cronk, David Goddard, David Graham, Dean Phillips, Deeksha Prabhu, Deepak Kejariwal, Dhirendra Garg, Diane Lonsdale, Dianne Butterworth, Donna Clements, Drew Bradman, Duncan Blake, Elizabeth Mather, Ewan O'Farrell, Florian Markowetz, Fran Adams, Francesca Pesola, Gareth Forbes, Gary Taylor, Glenn Collins, Gordon Irvine, Gysbert Fourie, Harriet Doyle, Heather Barnes, Helen Bowyer, Helen Whiting, Ian Beales, Ian Binnian, Ian Bremner, Ian Jennings, Ilona Troiceanu, Ines Modelell, Ingrid Emmerson, Jacobo Ortiz, Jacqueline Lilley, Jacquelyn Harvey, Jacqui Vicars, Jagjit Takhar, James Larcombe, Jan Bornschein, Jehad Aldegather, Jenny Johnson, Jill Ducker, Jo Skinner, Joanne Dash, Joanne Walsh, Jose Miralles, Josephine Ridgway, Julia Ince, Julie Kennedy, Kat Hampson, Kate Milne, Katherine Ellerby, Katherine Priddis, Kathy Rainsbury, Kelly Powell, Kerry Gunner, Krish Ragunath, Kyle Knox, Laura Baseley, Lauren White, Laurence Lovat, Lee Berney, Lindsay Crockett, Lisa Murray, Lisa Westwood, Lisa Chalkley, Loraine Leggett, Louise Dale, Louise Scovell, Lucy Brooks, Lucy Saunders, Lydia Owen, Maria Dilwershah, Marie Baldry, Marie Corcoran, Marie Roy, Mario Macedo, Mark Attah, Mary-Jo Anson, Matt Rutter, Matthew Wallard, Matthew Gaw, Matthew Hunt, Megan Lea-Hagerty, Melchizedek Penacerrada, Michele Bianchi, Michelle Baker-Moffatt, Michelle Czajkowski, Michelle Sleeth, Nick Brewer, Nick Wooding, Nicky Todd, Nicola Millen, Olga Zolle, Orla Whitehead, Patrick Ojechi, Patrick Moore, Paul Banim, Paula Spellar, Pradeep Bhandari, Prashant Kant, Rachel Nixon, Rebecca Russell, Rebekah Roberts, Rene Skule, Richard West, Robin Fox, Ruth Beesley, Ruth Gibbins, Ruth Osborne, S Thiagarajan, Sally Bastiman, Samantha Warburton, Samir Pai, Sarah Leith-Russell, Sarah Utting, Sarah Watson, Sarah Wytrykowski, Satish Singh, Shalini Malhotra, Sharon Woods, Shaun Conway, Sherrie Mateer, Shona Macrae, Shruti Singh, Simona Fourie, Siobhan Campbell, Siobhan Parslow-Williams, Sonica Goel, Stephen Dellar, Stephen Jones, Steve Knight, Stuart Mackay-Thomas, Stuti Mukherjee, Sue Allen, Suzanne Henry, Tara Evans, Theresa Leighton, Tim Bray, Tom Shackleton, Vanaja Santosh, Vicki Glover, Vijay Chandraraj, Will Elson, William Briggs, Zoe Barron, Zohrah Khan, Peter Sasieni

**Affiliations:** a MRC Cancer Unit, Hutchison-MRC Research Centre, University of Cambridge, Cambridge, UK; b The Primary Unit, Department of Public Health and Primary Care, University of Cambridge, Cambridge, UK; c Cambridge University Hospitals National Health Service Foundation Trust, Cambridge, UK; d Cancer Research UK and King's College London Cancer Prevention Trials Unit, School of Cancer and Pharmaceutical Sciences, King's College London, London, UK; e Cancer Prevention Group, School of Cancer and Pharmaceutical Sciences, King's College London, London, UK; f Cancer Research UK Cambridge Institute, University of Cambridge, Cambridge, UK; g Leeds Institute of Health Sciences, University of Leeds, Leeds, UK; h Institute of Population Health Sciences, Newcastle University, Sir James Spence Institute, Royal Victoria Infirmary, Newcastle upon Tyne, UK

## Abstract

**Background:**

Treatment of dysplastic Barrett's oesophagus prevents progression to adenocarcinoma; however, the optimal diagnostic strategy for Barrett's oesophagus is unclear. The Cytosponge-trefoil factor 3 (TFF3) is a non-endoscopic test for Barrett's oesophagus. The aim of this study was to investigate whether offering this test to patients on medication for gastro-oesophageal reflux would increase the detection of Barrett's oesophagus compared with standard management.

**Methods:**

This multicentre, pragmatic, randomised controlled trial was done in 109 socio-demographically diverse general practice clinics in England. Randomisation was done both at the general practice clinic level (cluster randomisation) and at the individual patient level, and the results for each type of randomisation were analysed separately before being combined. Patients were eligible if they were aged 50 years or older, had been taking acid-suppressants for symptoms of gastro-oesophageal reflux for more than 6 months, and had not undergone an endoscopy procedure within the past 5 years. General practice clinics were selected by the local clinical research network and invited to participate in the trial. For cluster randomisation, clinics were randomly assigned (1:1) by the trial statistician using a computer-generated randomisation sequence; for individual patient-level randomisation, patients were randomly assigned (1:1) by the general practice clinics using a centrally prepared computer-generated randomisation sequence. After randomisation, participants received either standard management of gastro-oesophageal reflux (usual care group), in which participants only received an endoscopy if required by their general practitioner, or usual care plus an offer of the Cytosponge-TFF3 procedure, with a subsequent endoscopy if the procedure identified TFF3-positive cells (intervention group). The primary outcome was the diagnosis of Barrett's oesophagus at 12 months after enrolment, expressed as a rate per 1000 person-years, in all participants in the intervention group (regardless of whether they had accepted the offer of the Cytosponge-TFF3 procedure) compared with all participants in the usual care group. Analyses were intention-to-treat. The trial is registered with the ISRCTN registry, ISRCTN68382401, and is completed.

**Findings:**

Between March 20, 2017, and March 21, 2019, 113 general practice clinics were enrolled, but four clinics dropped out shortly after randomisation. Using an automated search of the electronic prescribing records of the remaining 109 clinics, we identified 13 657 eligible patients who were sent an introductory letter with 14 days to opt out. 13 514 of these patients were randomly assigned (per practice or at the individual patient level) to the usual care group (n=6531) or the intervention group (n=6983). Following randomisation, 149 (2%) of 6983 participants in the intervention group and 143 (2%) of 6531 participants in the usual care group, on further scrutiny, did not meet all eligibility criteria or withdrew from the study. Of the remaining 6834 participants in the intervention group, 2679 (39%) expressed an interest in undergoing the Cytosponge-TFF3 procedure. Of these, 1750 (65%) met all of the eligibility criteria on telephone screening and underwent the procedure. Most of these participants (1654 [95%]; median age 69 years) swallowed the Cytosponge successfully and produced a sample. 231 (3%) of 6834 participants had a positive Cytosponge-TFF3 result and were referred for an endoscopy. Patients who declined the offer of the Cytosponge-TFF3 procedure and all participants in the usual care group only had an endoscopy if deemed necessary by their general practitioner. During an average of 12 months of follow-up, 140 (2%) of 6834 participants in the intervention group and 13 (<1%) of 6388 participants in the usual care group were diagnosed with Barrett's oesophagus (absolute difference 18·3 per 1000 person-years [95% CI 14·8–21·8]; rate ratio adjusted for cluster randomisation 10·6 [95% CI 6·0–18·8], p<0·0001). Nine (<1%) of 6834 participants were diagnosed with dysplastic Barrett's oesophagus (n=4) or stage I oesophago-gastric cancer (n=5) in the intervention group, whereas no participants were diagnosed with dysplastic Barrett's oesophagus or stage I gastro-oesophageal junction cancer in the usual care group. Among 1654 participants in the intervention group who swallowed the Cytosponge device successfully, 221 (13%) underwent endoscopy after testing positive for TFF3 and 131 (8%, corresponding to 59% of those having an endoscopy) were diagnosed with Barrett's oesophagus or cancer. One patient had a detachment of the Cytosponge from the thread requiring endoscopic removal, and the most common side-effect was a sore throat in 63 (4%) of 1654 participants.

**Interpretation:**

In patients with gastro-oesophageal reflux, the offer of Cytosponge-TFF3 testing results in improved detection of Barrett's oesophagus. Cytosponge-TFF3 testing could also lead to the diagnosis of treatable dysplasia and early cancer. This strategy will lead to additional endoscopies with some false positive results.

**Funding:**

Cancer Research UK, National Institute for Health Research, the UK National Health Service, Medtronic, and the Medical Research Council.

## Introduction

Heartburn symptoms caused by gastro-oesophageal reflux disease are common, affecting up to 20% of adults in northwest Europe, North America, Australia, and New Zealand and leading to enormous annual health-care costs.^1^, ^2^ Most of these individuals do not have a diagnosis and are treated over many years with acid-suppressant medication therapy. Symptoms of heartburn are important when one considers the link between heartburn and oesophago-gastric cancer.^3^ It is estimated that 3–6% of individuals with gastro-oesophageal reflux-predominant symptoms could have the precursor lesion to oesophageal adenocarcinoma, known as Barrett's oesophagus. However, only around 20% of patients with Barrett's oesophagus are diagnosed. Therefore, most cases of oesophageal adenocarcinoma are diagnosed de novo, without the opportunity to prevent progression.^4^, ^5^, ^6^

The incidence of oesophageal adenocarcinoma is six times higher than it was in the 1990s. Oesophageal adenocarcinoma also has a dismal prognosis due to late presentation, with an overall 5-year survival of less than 20%, despite advances in neoadjuvant therapy and surgery.^7^, ^8^ Clinical guidelines recommend urgent referral for an endoscopy in patients with warning symptoms, such as dysphagia and weight loss, and routine referral for an endoscopy in those with symptoms of gastro-oesophageal reflux that persist despite recommended lifestyle and pharmacological management strategies, and those with multiple additional risk factors for the disease.^9^, ^10^, ^11^, ^12^ Nevertheless, the proportion of patients referred for an endoscopy from general practice clinics varies widely, and the referral rates per practice correlate with the stage at diagnosis.^13^ A modelling study^14^ using data from the USA estimated that the burden of oesophageal adenocarcinoma could be reduced by up to 50% through implementing strategies for the systematic screening and early diagnosis of individuals with gastro-oesophageal reflux, who would otherwise not have been referred for an endoscopy.

Early detection needs to be combined with effective interventions to be clinically beneficial. There have been important advances in outpatient-based endoscopic therapies, which are now recommended for low-grade and high-grade dysplasia in Barrett's oesophagus, with low rates of recurrence.^15^, ^16^, ^17^ Patients with intramucosal stage I cancers have a survival of more than 90% and can be treated endoscopically, thus mitigating the risks of and side-effects from systemic therapy and an oesophagectomy, which is often required for more advanced disease.^18^, ^19^

**Research in context**

**Evidence before this study**

Barrett's oesophagus is a precancerous condition, which, if diagnosed, can permit early detection and curative treatment of dysplasia and oesophageal adenocarcinoma. The Cytosponge-trefoil factor 3 (TFF3) test is a novel non-endoscopic cell collection device coupled with an immunohistochemical biomarker that can diagnose Barrett's oesophagus in the primary care setting. Clinical studies to date have shown promising data on the safety, acceptability, accuracy, and cost-effectiveness of this technique.

**Added value of this study**

This is the first randomised controlled trial to show that offering the Cytosponge-TFF3 test to patients taking acid-suppressant therapy for symptoms of gastro-oesophageal reflux in primary care improves the detection of Barrett's oesophagus and early cancer when compared with usual clinical practice.

**Implications of all the available evidence**

Improved detection of Barrett's oesophagus, high patient acceptability, and few adverse events suggest that the Cytosponge-TFF3 technique could be adopted as a diagnostic triage test on a large scale.

**Figure 1:**
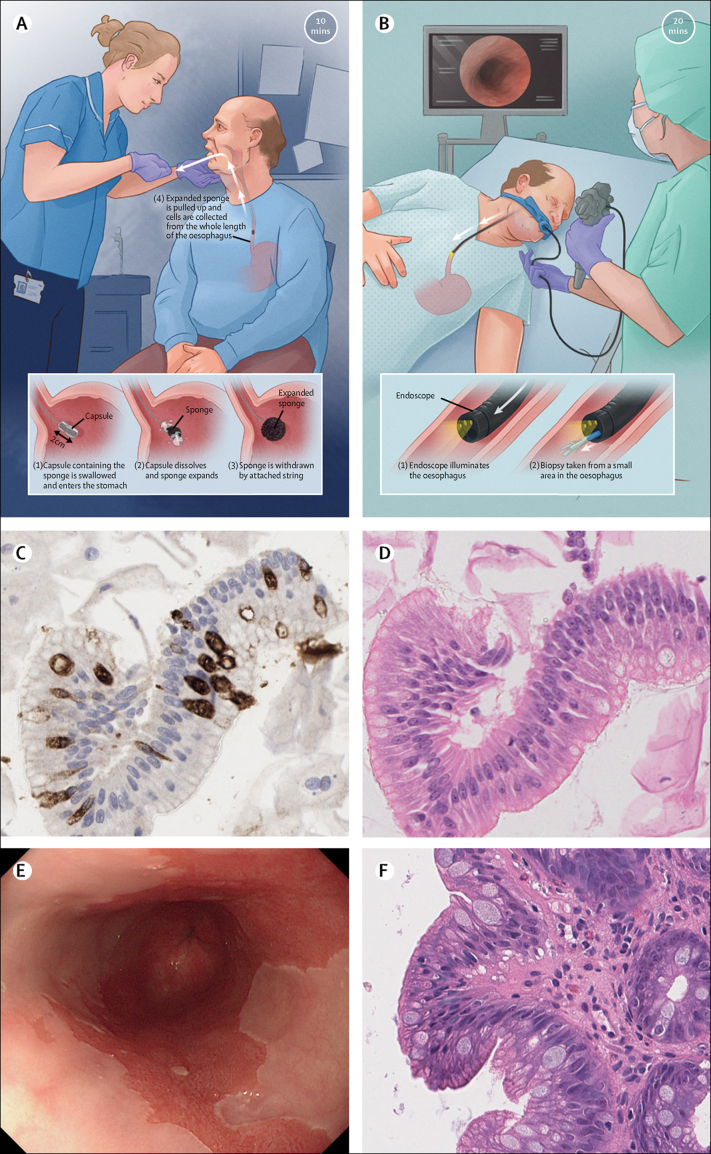
Comparison of the Cytosponge-TFF3 procedure with the endoscopy procedure (A) Administration and passage of the Cytosponge-TFF3 device to obtain a sample of oesophageal epithelial cells. (B) Administration and passage of an endoscope to visualise the oesophagus. The Cytosponge-TFF3 sample is processed to a paraffin block and stained with an antibody against (C) TFF3 and with (D) haematoxylin and eosin (magnification × 200). (E) Endoscopic white light view of Barrett's oesophagus in deep red compared with surrounding light pink squamous epithelium. (F) One or more endoscopic biopsies are taken and the tissues are stained with haematoxylin and eosin for histopathological assessment (magnification × 200). (A) and (B) were drawn by Campbell Medical Illustration (Glasgow, Scotland). TFF3=trefoil factor 3.

In view of the scale of gastro-oesophageal reflux disease, and the costs (both psychological and financial) of investigation, any new clinical strategy needs to be carefully evaluated. We have developed a test for Barrett's oesophagus that is suitable for use in the primary care setting. The test comprises a non-endoscopic cell collection device coupled with an in vitro test for the specific biomarker, trefoil factor 3 (TFF3), that identifies intestinal metaplasia (the histopathological hallmark of Barrett's oesophagus;^20^ figure 1). Thus far, two clinical studies^21^, ^22^ of this new clinical strategy, termed the Cytosponge-TFF3 procedure, have been done in over 2000 patients, with promising data on safety, acceptability, accuracy, and cost-effectiveness.^23^, ^24^, ^25^

We did this pragmatic, randomised controlled trial, involving patients with recurrent symptoms of gastro-oesophageal reflux who had been taking acid-suppressant medication prescribed by their general practitioner, to investigate whether the Cytosponge-TFF3 test, administered in the community setting, leads to the identification of more patients with Barrett's oesophagus than does usual clinical practice for endoscopy referral in England.

## Methods

### Study design and participants

This multicentre, pragmatic, randomised controlled trial took place in 109 socio-demographically diverse general practice clinics in England (appendix pp 129–130).

Patients were eligible for inclusion if they were aged 50 years or older and had records of having been prescribed acid-suppressant therapy (proton-pump inhibitor or histamine-2 receptor antagonists) for at least 6 months in the previous year. Patients with records of having been prescribed non-steroidal anti-inflammatory drugs together with acid-suppressant therapy, suggesting that their reflux symptoms were not the primary basis for the proton-pump inhibitor prescription, and patients who had undergone an endoscopy in the previous 5 years or with a previous diagnosis of Barrett's oesophagus, were excluded from the study.

All potential participants received an introductory letter to the study and were given 14 days to opt out, after which point they were enrolled in the trial.

The study protocol, which was approved by a central ethics committee, has been made publicly available (appendix pp 3–86).^26^ Aggregated data were collected from participating primary care clinics for all potential participants who did not opt out. Written informed consent was obtained before collecting any individual-level patient data and before any clinical procedure was done.

### Randomisation and masking

Initially, general practice clinics (ie, clusters) were randomly assigned (1:1) to either the usual care group, in which eligible patients with gastro-oesophageal reflux under the care of these clinics received standard management of their symptoms and were only referred for an endoscopy if required, or the intervention group, in which eligible patients received standard management and were offered the Cytosponge-TFF3 procedure, with a subsequent endoscopy if the procedure identified TFF3-positive cells. Approximately two-thirds of the way through recruitment, the trial switched to an individual patient-level randomisation approach, which was approved by an independent trial steering committee, the research ethics committee, and the Medicines and Healthcare products Regulatory Agency (MHRA). Cluster randomisation was initially chosen in order to remove selection bias by general practitioners; however, in the trial, all patients were selected by use of the prescribing database, so selection bias was not an issue. After recommendation by the trial steering committee, we switched to individual randomisation during the study, which substantially increased the statistical power and also satisfied patient and clinician demand for the Cytosponge procedure to be available in all clinics. Data from both the cluster and individual randomisations were analysed separately before they were combined, having established that their results were consistent, as required by an independent data monitoring committee and the MHRA (appendix pp 87–89).

The trial statistician did the cluster randomisation of general practice clinics by randomly sorting practices within strata (using computer-generated random number sequences) and then allocating alternately. Clinics were not randomly assigned until they had agreed to participate. Individual patient-level randomisation was done by the general practice clinics directly using the BEST3 app, which used a computer-generated random number sequence. Potential participants in both the clinic-level and the patient-level randomisations were informed about the research and given the option to opt out of participation (including data collection) before knowing which group they would be assigned to.

All patients who were randomly assigned were followed passively for a weighted overall average of approximately 12 months (range 8–18 months). The chief investigator (RCF) and the lead statistician (PS) were masked to the aggregated results by group until follow-up was complete. Pathologists analysing endoscopic biopsies for Barrett's oesophagus did not know whether the patient had undergone a Cytosponge-TFF3 procedure.

### Procedures

Participants randomly assigned to the usual care group received standard care, in which they received prescriptions for acid-suppressant medication and their general practitioner might have provided lifestyle advice or referral for an endoscopy, depending on the severity of their symptoms. Participants randomly assigned to the intervention group received a letter inviting them to undergo a Cytosponge-TFF3 test and, if they expressed interest, were subsequently screened by a nurse via a telephone interview. Sometimes patients were not contactable by telephone or they changed their mind in the intervening period. The telephone screening interview included a symptom screen to ascertain whether participants were taking acid-suppressant therapy for heartburn-predominant symptoms and to exclude any participants who were not deemed to be suitable for the Cytosponge-TFF3 procedure. Participants were not offered a Cytosponge-TFF3 test if they had dysphagia (as the capsule might not reach the distal oesophagus) or if they were at an increased risk of bleeding because of known cirrhosis or varices, or if they were unable to stop taking anticoagulants (see appendix p 26 for a full list of ineligibility criteria). Such participants were still included in the intention-to-treat analysis.

The Cytosponge consists of a polyester, medical-grade mesh sphere (3 cm in diameter), compressed within a gelatin capsule and attached to a thread (Cambridge University Hospitals NHS Foundation Trust [legal manufacturer]; produced by Europlaz, Essex, UK). The device was administered by a general practice clinic nurse or a Clinical Research Network nurse, following a training seminar and one-to-one training with an experienced practitioner (ID-B), until they were signed off as competent.

Samples collected from the Cytosponge procedure were processed centrally and assessed for the presence of Barrett's oesophagus by use of haematoxylin and eosin staining and immunohistochemical staining for TFF3 (Ventana Medical Systems, Tuscon, AZ, USA), as described previously.^20^ TFF3 staining was evaluated by experienced upper gastrointestinal pathologists, and consensus agreement from two or three pathologists was used in any cases of uncertainty. A sample in which no glandular cells were present was deemed to provide a low-confidence result, as the device might not have reached the stomach and a diagnosis of distal Barrett's oesophagus might have therefore been missed. Any sample with glandular groups of cells (indicating that the device had reached the stomach), and that did not have equivocal TFF3 staining, was considered a high-confidence result. Patients with low-confidence or equivocal results, and any with processing failure, were offered a repeat Cytosponge-TFF3 test. All patients with a positive TFF3 test result were offered an endoscopy to confirm the diagnosis of Barrett's oesophagus and inform treatment. After completion of trial follow-up, a random sample of participants from each study group were invited to undergo a research endoscopy procedure. The results of these research endoscopies will be presented elsewhere. All endoscopy samples (both in the usual care group and in the intervention group) were analysed by the local pathologist. Participants with Barrett's oesophagus diagnosed by use of the Cytosponge-TFF3 test also had their endoscopic biopsies centrally reviewed to confirm that intestinal metaplasia was present and to identify any dysplasia or cancer (by haematoxylin and eosin staining).

A census date 8–18 months after randomisation was set for each general practice clinic. Passive follow-up of all participants, irrespective of study group or whether they had undergone a Cytosponge-TFF3 procedure, was done up to the census date. Census dates were chosen independently of the randomisation, so as to have a weighted average follow-up of 12 months. The endpoint data collected were coded diagnoses of Barrett's metaplasia, Barrett's dysplasia, or oesophago-gastric adenocarcinoma, ascertained by at least one of the following three methods: (1) an electronic search of general practice clinic records for new diagnoses of Barrett's oesophagus or oesophageal adenocarcinoma, new referrals to gastroenterology departments, or new referrals for esophagogastroduodenoscopy procedures within the study period, followed by a manual search of the clinical records for those patients with a potential diagnosis of Barrett's oesophagus or oesophago-gastric adenocarcinoma identified by the electronic search; (2) a full manual search of the general practice clinic records for all participants registered with that clinic; and (3) secure anonymous record linkage between participating general practice clinics and participating endoscopy units to identify individuals who were both study participants and who had been diagnosed with Barrett's oesophagus or oesophago-gastric adenocarcinoma during the study period.

### Outcomes

The primary outcome was the diagnosis of Barrett's oesophagus at 12 months after enrolment, expressed as rate per 1000 person-years, in all participants in the intervention group (regardless of whether they had accepted the offer of the Cytosponge-TFF3 procedure) compared with all participants in the usual care group. The secondary outcomes were as follows: uptake of the Cytosponge-TFF3 procedure; the number of cases of Barrett's oesophagus with dysplasia and intestinal metaplasia-associated cancer, by stage at diagnosis; the positive predictive value of the Cytosponge-TFF3 test, measured in the subset of patients who had a subsequent endoscopy after testing positive for TFF3; and the acceptability and safety of the Cytosponge-TFF3 test.

### Statistical analysis

The results were statistically analysed by RM, MG, and PS. The statistical analysis plan for the study is provided in appendix (pp 90–125).

By use of a series of key assumptions about the prevalence of Barrett's oesophagus, the proportion of endoscopy referrals, and the sensitivity and uptake of the Cytosponge-TFF3 procedure (appendix pp 103–107), the expected proportions of Barrett's oesophagus diagnoses over 12 months were calculated as 1·38% in the intervention group and 0·60% in the usual care group. On the basis of these assumptions, we calculated that a sample size of 6764 patients was required to achieve a power of 90% and a significance level of 5% if randomisation was done at the individual patient level.

To account for the cluster-randomisation design, a variance inflation factor was estimated by strata (defined by number of patients from each clinic who were randomly assigned; 48–65, 66–90, 91–125, 126–175 or 176–198 patients) for the cluster-randomised group, assuming that the intraclass correlation coefficient of the proportion of patients with Barrett's oesophagus was 0·025. The actual numbers of participants in each strata were divided by the variance inflation factor to yield the equivalent numbers of individually-randomised patients. Throughout the trial, we ensured that the projected sum of the equivalent size of the cluster-randomised group and the size of the individual patient-level randomised group was at least 6764 participants.

The primary endpoint of Barrett's oesophagus diagnoses (excluding cancer diagnoses) in both groups at 12 months after enrolment, was analysed by use of a random-effects log-linear model. The number of Barrett's oesophagus diagnoses was the Poisson-distributed outcome, with a fixed effect for the strata, a random effect for each clinic, and an offset for the number of person-years of follow-up. We assumed two different treatment effects (fixed rate ratios [RRs]) for the intervention group (one in the first 4 months and the second thereafter) that were eventually combined at a weight ratio of 1:2. In the usual care group, the treatment effect was assumed to be constant over time. The analysis was first done for the cluster-randomised group, then for the individual patient-randomised group (with no cluster effect), and finally for the whole dataset. When analysing the whole dataset, the individual patient-randomised group was assigned to a separate stratum. This method was approved by the MHRA.

As only aggregated data about age and sex were available, and we only had access to individual-level data on age, sex, and medication history for patients who successfully swallowed the Cytosponge, no adjustment was made for these factors in the analysis of the primary outcome. Statistical significance was based on a two-sided test with an α-value of 5%.

The uptake of the Cytosponge-TFF3 procedure was assessed as the number of patients who successfully swallowed the capsule, expressed as a proportion of the patients who were offered the procedure. The number of patients with Barrett's oesophagus, Barrett's oesophagus with dysplasia, or Barrett's oesophagus and cancer is reported by study group and also by the number of participants who underwent the Cytosponge-TFF3 procedure in the intervention group. The positive predictive value of the Cytosponge-TFF3 procedure was calculated from the proportion of patients who underwent the procedure, in whom the subsequent endoscopy and pathological assessment confirmed the diagnosis of Barrett's oesophagus, Barrett's dysplasia, or cancer (gold standard).

The acceptability of the Cytosponge-TFF3 procedure was estimated from a questionnaire, in which participants rated the procedure using an 11-point visual analogue scale (from 0 to 10); the median and IQR are reported, together with the proportion of participants who scored 5 or more (indicating that the test was somewhat acceptable).

The safety of the Cytosponge-TFF3 procedure was assessed by recording any adverse events and serious adverse events that had occurred within 7–14 days of undergoing the procedure. This assessment was done proactively by a nurse via a telephone call with patients. The proportion of patients who had an adverse event, and the type and severity of adverse event, is reported. The adverse events were only collected for participants undergoing the Cytosponge-TFF3 procedure. Since endoscopy is standard of care, no adverse event data was collected in relation to this procedure.

Statistical analyses were done in Stata version 15 (StataCorp LLC, College Station, TX, USA). Pseudo-random numbers for all randomisations were generated in R (R Core Team [2019]).

An independent data monitoring committee and a trial steering committee, which included two lay members who provided a patient's perspective, oversaw the trial.

The trial is registered with the ISRCTN registry, number ISRCTN68382401.

### Role of the funding source

The funders of the study had no role in study design, data collection, data analysis, data interpretation, or writing of the report. The corresponding author (RCF), RM, MG, BM, and PS had full access to all the data in the study and had final responsibility for the decision to submit for publication.

## Results

**Figure 2:**
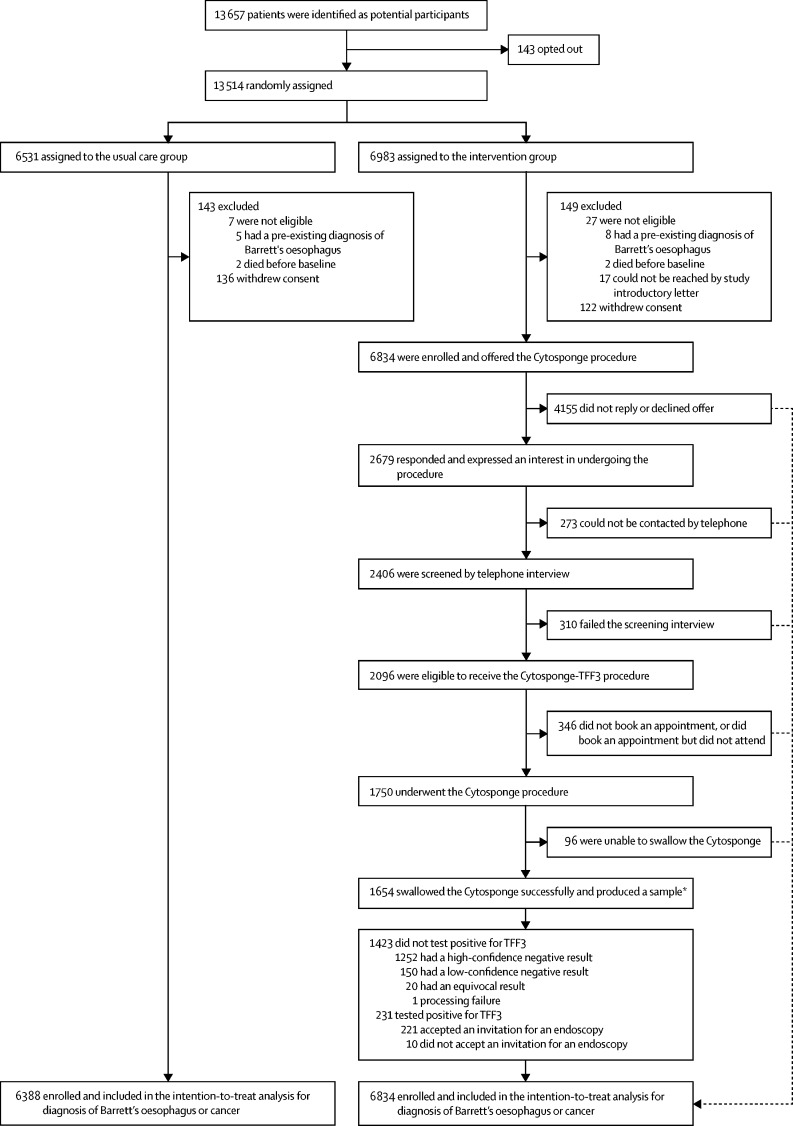
Trial profile *202 (12%) of these 1654 participants had a repeat Cytosponge test, as the first sample yielded a low-confidence result (defined as the absence of glandular cells in the sample) and a diagnosis of Barrett's oesophagus could have therefore been missed; patients with equivocal results, or technical or processing failures, were also invited for a repeat test. TFF3=trefoil factor 3.

Between March 20, 2017, and March 21, 2019, 113 general practice clinics located in socio-demographically diverse regions in England were enrolled, but four clinics dropped out shortly after being randomly assigned (three in the usual care group and one in the intervention group), leaving 109 clinics, comprising 13 657 patients. These patients were sent an introductory letter and given 14 days to opt out of the study. 143 of these patients opted out before individual patient-level randomisation, leaving 13 514 patients to be randomly assigned. After randomisation, 136 patients in the usual care group and 122 patients in the intervention group withdrew. Additionally, 17 patients (ten in the intervention group and seven in the usual care group) were excluded because they had either died or had been diagnosed with Barrett's oesophagus before randomisation, and 17 patients (all in the intervention group) were excluded because their contact details were absent. Of the remaining 13 222 enrolled patients, 7839 patients from 75 clinics were cluster-randomised (appendix p 127), and 5383 patients from 34 clinics were individually randomised (appendix p 128). Overall, 6388 participants were randomly assigned to the usual care group and 6834 participants to the intervention group (figure 2).

The demographics of the 13 222 participants included in the final analysis are summarised (table 1). The age distribution of participants who successfully swallowed the Cytosponge was similar to that of all participants. The randomly assigned clinics represented all ten deciles of the Index of Multiple Deprivation (data not shown). The median decile of deprivation among participants was seven (with one being the most deprived and ten the least deprived) and the lower quartile was four.

**Table 1:** Baseline characteristics of all randomly assigned participants

	**All participants (n=13 222)**	**Usual care group (n=6388)**	**Intervention group**	
			All participants (n=6834)	Participants who successfully swallowed the Cytosponge (n=1654)
**Sex**				
Male	6030 (46%)*	..	..	796 (48%)
Female	7155 (54%)*	..	..	858 (52%)
**Age distribution, years**				
50–59	3171 (24%)*	..	..	326 (20%)
60–69	4001 (30%)*	..	..	562 (34%)
70–79	4172 (32%)*	..	..	615 (37%)
80–89	1642 (12%)*	..	..	140 (8%)
90–99	199 (2%)*	..	..	11 (1%)
**Size of general practice surgery**				
Small (48–90 patients)	2083 (16%)	1038 (16%)	1045 (15%)	..
Medium (91–160 patients)	6746 (51%)	3071 (48%)	3675 (54%)	..
Large (161–231 patients)	4393 (33%)	2279 (36%)	2114 (31%)	..
**Medication use**				
Proton-pump inhibitor only	11 818 (92%)†	..	..	1434 (87%)‡
Histamine-2 receptor antagonists only	613 (5%)†	..	..	170 (10%)‡
Proton-pump inhibitor plus histamine-2 receptor antagonist	413 (3%)†	..	..	43 (3%)‡
**Socioeconomic factors**				
Median Index of Multiple Deprivation decile§	7 (4–9)	6 (4–9)	7 (5–9)	..

Data are n (%) or median (IQR). Most data were aggregated by site; therefore, there are no data for some fields.

* Baseline data were available in an aggregated form; data for age and sex are missing from one site.

† Baseline data were available in an aggregated form; data for medication are missing from six sites.

‡ Data for seven patients are not shown in the table, as they had records of over-the-counter acid-suppressant or other medications.

§ The Index of Multiple Deprivation (with a score of 1 indicating most deprived and a score of 10 indicating least deprived) scores were not available for individual participants and were calculated by assigning the score for each general practice clinic to each patient.

Following a written invitation, 2679 (39%) of 6834 patients in the intervention group responded and expressed an interest in taking part in the Cytosponge-TFF3 procedure. Of these, 2096 (78%) participants were eligible following the telephone assessment, and 1750 (65%) provided consent and underwent the procedure. 1654 (95%) of these participants (and 24% of all 6834 participants in the intervention group) successfully swallowed the device, including 796 men (48%) and 858 (52%) women, with a median age of 69 years (IQR 61–74; table 1). 311 (19%) of the 1654 participants who had successfully swallowed the device had a low-confidence negative or equivocal test result, and depending on local capacity, were invited for a repeat Cytosponge-TFF3 test. 202 (65%) of these participants attended the repeat appointment, 190 (94%) of whom successfully swallowed the device, leading to a further 140 patients producing a high-confidence (positive or negative) result. Overall, after the repeat test, 150 (9%) of the 1654 patients who successfully swallowed the Cytosponge-TFF3 still produced a low-confidence negative result (figure 2).

Apart from the eight participants who were found, on review, to have pre-existing Barrett's oesophagus, all participants who were invited for the Cytosponge-TFF3 procedure were included in the final intention-to-treat analysis, regardless of whether or not they accepted the invitation. Barrett's oesophagus diagnoses in both groups had to be identified from records of clinical coded diagnoses at all general practice clinics included in the study, the electronic records of local referral hospitals, or both, to ensure equity across the usual care and intervention groups (otherwise, diagnoses from the intervention group would have been more easily ascertained). One diagnosis of Barrett's oesophagus in a patient who had a positive Cytosponge-TFF3 test result was omitted from the results, as a coded diagnosis was not identified by any of these data collection methods.

We identified 140 Barrett's oesophagus diagnoses in the intervention group (127 in patients who underwent the Cytosponge-TFF3 procedure, and 13 in patients who did not undergo the Cytosponge-TFF3 procedure) compared with 13 diagnoses in the usual care group (table 2, 3; see appendix pp 131–32 for the corresponding tables for randomisation groups and a breakdown of the length of Barrett's oesophagus detected). 87 (69%) of the 127 participants who were diagnosed with Barrett's oesophagus from the Cytosponge-TFF3 procedure were male. As the results of the cluster-level randomisation and patient-level randomisation both favoured the intervention group, an overall RR was calculated (table 2). The estimated cumulative rate of Barrett's oesophagus at 12 months was 20·2 per 1000 person-years in the intervention group and 2·0 per 1000 person-years in the usual care group (RR adjusted for cluster randomisation 10·6 [95% CI 6·0–18·8], p<0·0001; table 2).

**Table 2:** Barrett's oesophagus diagnoses in the usual care group compared with the intervention group

	**Usual care group (n=6388)**	**Intervention group (n=6834)**	**Absolute difference in rates per 1000 person-years (95% CI)**	**Overall rate ratio (95% CI); p value**	**Adjusted rate ratios (95% CI); p value**		
					Cluster randomised group	Patient-level randomised group	Overall*
Number of participants diagnosed with Barrett's oesophagus	13 (<1%)	140 (2%)†	..	..	..	..	..
Follow-up, person-years	6579	6952	..	..	..	..	..
Rate of Barrett's oesophagus, per 1000 person-years	2·0	20·2‡	18·3 (14·8–21·8)	10·2 (5·8–18·1)	10·0 (5·0–20·0); p<0·0001	12·0 (4·3–33·2); p<0·0001	10·6 (6·0–18·8); p<0·0001

Data are n (%), unless otherwise specified.

* Overall adjusted rate ratio is a combined rate ratio of the two randomisation groups (cluster randomisation and individual patient-level randomisation) and accounts for clustering.

† Number of participants diagnosed with Barrett's oesophagus in the intervention group includes all participants who were offered the Cytosponge procedure.

‡ The rate of Barrett's oesophagus in the intervention group was calculated as the weighted average of the rate in the first 4 months of follow-up and the rate in the following months, with a weight ratio of 1:2.

Of 1654 participants in the intervention group who successfully swallowed the Cytosponge device, 221 (13%)with a positive TFF3 result had a subsequent confirmatory endoscopy. 127 (57%) of these participants were diagnosed with Barrett's oesophagus (one of whom had low-grade dysplasia, and three of whom had high-grade dysplasia), and four (2%) participants were diagnosed with stage I oesophago-gastric cancer. Therefore, the Cytosponge-TFF3 procedure had a positive predictive value of 59% (131 of 221 confirmatory endoscopies in patients with a positive Cytosponge-TFF3 result) for Barrett's oesophagus, dysplasia, or oesophago-gastric cancer (table 2, 3). Of those 90 participants who received a confirmatory endoscopy that did not result in a diagnosis of Barrett's oesophagus, dysplasia, or cancer, a further 33 (37%) participants had intestinal metaplasia, identified from a single biopsy taken from the cardia or at the gastro-oesophageal junction.

Using the available data, we calculated the empirical intraclass correlation coefficient of the proportion of patients with Barrett's oesophagus, and found that this value was similar to the expected empirical intraclass correlation coefficient (approximately 0·025).

For the secondary endpoints, we compared the number of endoscopic diagnoses of dysplasia and cancer in participants who were offered the Cytosponge-TFF3 procedure with the number of these diagnoses in participants in the usual care group (intention-to-treat analysis). Nine (<1%) of 6834 participants were diagnosed with dysplastic Barrett's oesophagus (n=4) or stage I oesophago-gastric cancer (n=5) in the intervention group, whereas no participants were diagnosed with dysplastic Barrett's oesophagus or stage I oesophago-gastric cancer in the usual care group (table 3). Of these nine participants in the intervention group, eight were detected as a result of a positive Cytosponge-TFF3 test and a subsequent endoscopy and have all undergone a curative intervention (seven participants underwent endoscopic therapies, and one participant underwent an oesophagectomy for a stage IB cancer involving the first layer of the submucosa; appendix p 133). Among those who were offered the Cytosponge-TFF3 procedure but did not have the test (n=5084), one participant, who initially expressed interest in the procedure, but was referred for an endoscopy before it could be done, was diagnosed with early-stage cancer. Of all 6388 participants in the usual care group included in the final analysis, three participants were diagnosed with cancer, of whom two participants were palliative at presentation and died during the study period (appendix p 133). In the intervention group, two participants who did not undergo the Cytosponge-TFF3 test were diagnosed with stage IV oesophago-gastric cancer.

**Table 3:** Number of individuals with Barrett's oesophagus in the usual care group and intervention group with or without cancer, by grade of dysplasia and cancer stage

		**Usual care group (n=6388)**	**Intervention group**		
			Underwent the Cytosponge procedure (n=1750)	Did not undergo the Cytosponge procedure (n=5084)	Overall (n=6834)
Grade of dysplastic Barrett's oesophagus					
	No dysplasia	13	116	13	129
	Indefinite	0	7	0	7
	Low-grade	0	1	0	1
	High-grade	0	3	0	3
	Total	13	127	13	140
Oesophago-gastric cancer stage					
	I	0	4	1	5
	II	1	0	0	0
	III	1	0	0	0
	IV	1	0	2	2
Total number of participants with Barrett's oesophagus, cancer, or both		16	131	16	147

In the intervention group, an acceptability score for the Cytosponge-TFF3 procedure was provided by 1464 (89%) of 1654 participants approximately 1 week after they underwent the procedure. The median acceptability score was 9 (IQR 8–10), with 10 being completely acceptable, and 1427 (97%) of 1464 participants gave a score of 5 or higher (appendix p 134).

In the intervention group, one serious adverse event associated with the Cytosponge-TFF3 procedure was reported (detachment of the sponge from the thread requiring endoscopy to retrieve the expanded sponge with no adverse sequelae), and three serious adverse events unrelated to the procedure were reported (table 4). Of 1654 participants who successfully swallowed the Cytosponge device, 142 (9%) participants reported an adverse event, including 63 (4%) participants who had a sore throat that required medication or that interfered with eating (table 4).

**Table 4:** Adverse events in participants who underwent the Cytosponge-trefoil factor 3 (TFF3) procedure

	**Adverse event severity (n=142)**			**Total (n=142)**
	Low (n=112)	Moderate (n=23)	High (n=7)	
**Adverse event**				
Sore throat	57 (51%)	5 (22%)	1 (14%)	63 (44%)
Dyspepsia indigestion reflux	16 (14%)	3 (13%)	0	19 (13%)
Oesophageal or gastric pain	11 (10%)	2 (9%)	2 (29%)	15 (11%)
Feeling non-specifically unwell	6 (5%)	3 (13%)	0	9 (6%)
Nausea or vomiting	5 (4%)	3 (13%)	0	8 (6%)
Voice disturbance	3 (3%)	1 (4%)	0	4 (3%)
Diarrhoea or an upset stomach	4 (4%)	1 (4%)	0	5 (4%)
Chest pain or discomfort	2 (2%)	0	0	2 (1%)
Allergic reaction	1 (1%)	0	0	1 (1%)
Anxiety	1 (1%)	0	0	1 (1%)
Bad taste	1 (1%)	0	0	1 (1%)
Paroxysmal positional vertigo	1 (1%)	0	0	1 (1%)
Blood clot excretion	1 (1%)	0	0	1 (1%)
Vasovagal attack	1 (1%)	0	0	1 (1%)
Nosebleed	1 (1%)	0	0	1 (1%)
Headache	1 (1%)	1 (4%)	0	2 (1%)
Bloodshot eye	0	1 (4%)	0	1 (1%)
Chest infection	0	1 (4%)	0	1 (1%)
Abrasion	0	1 (4%)	0	1 (1%)
Fall	0	1 (4%)	0	1 (1%)
**Serious adverse event**				
Unconscious after minor accident	0	0	1 (14%)	1 (1%)
Detachment of the sponge on day of the procedure	0	0	1 (14%)	1 (1%)
Hernia*	0	0	1 (14%)	1 (1%)
Myocardial infarction†	0	0	1 (14%)	1 (1%)

Data are n (%). All percentages calculated with the total number of participants in that column who had an adverse event as the denominator. The severity of adverse events was classified as low, moderate, or high by the nurse during the proactive follow-up telephone call with the patient. Serious adverse events were those classified according to the regulatory requirement for a device trial.

* Hernia was repaired 5 days after the procedure.

† Occurred 3 days after the procedure.

## Discussion

In this multicentre, pragmatic, randomised controlled trial we found that an invitation to have a Cytosponge-TFF3 test led to increased diagnosis of Barrett's oesophagus when compared with usual care by general practitioners. This comparison was made in patients identified as being high-risk for this condition, on the basis of a systematic search of electronic patient records for anti-gastro-oesophageal reflux medication. With regard to the secondary endpoint of the proportion of cancer diagnoses, although the numbers were small, we found that all participants in the intervention group who had dysplasia and cancer identified by the Cytosponge-TFF3 procedure were suitable for curative therapy, whereas the cancers detected in the usual care group, and among participants who did not undergo a Cytosponge-TFF3 procedure, had more advanced disease (four of six participants had stage III and IV cancer) and two of these were palliative at presentation and died during the study period.

For a device to be suitable for use in general practice clinics, its uptake, safety, and acceptability are key. The acceptability data obtained in our study are encouraging, with a median acceptability score of 9 out of 10, consistent with our previous trials.^21^, ^22^ In this pragmatic trial done across a wide range of demographic areas across England, the proportion of all participants in the intervention group (n=6834) who expressed an interest in the Cytosponge-TFF3 procedure was 39% (n=2679), and 24% (n=1654) of participants had the procedure and successfully swallowed the device, after accounting for inclusion and exclusion criteria and scheduling limitations. Since we anticipate the Cytosponge-TFF3 procedure being offered by a patient's general practice clinician during a consultation for symptoms of gastro-oesophageal reflux or for a repeat prescription of acid-suppressant medication, as opposed to an unexpected written invitation, and since we will now be able to provide information regarding the efficacy of this procedure from this trial, we predict that the uptake of the Cytosponge-TFF3 procedure will increase substantially compared with that observed in this trial. This hypothesis will require further evaluation in future studies or in clinical implementation research.

The safety of the Cytosponge-TFF3 device has been evaluated previously in a systematic review^25^ of 2672 procedures done across four different studies in the UK, the USA, and Australia. In this review,^25^ 2334 (97%) of 2418 patients swallowed the device successfully and there were two adverse events associated with the device; one was a detachment and one was a self-limiting pharyngeal bleed. These results are similar to those of our trial. The proactive telephone call to patients 7–14 days after they underwent the procedure also allowed us to collect data on side-effects. We found that 63 (4%) of 1654 participants had a sore throat after the procedure, indicating that patients should be told that they might experience this adverse event after the procedure.

The prevalence of Barrett's oesophagus or cancer in the 221 participants who received an endoscopy after testing positive for TFF3 was 59% (n=131). We also identified intestinal metaplasia of the gastro-oesophageal junction and gastric cardia, which was extensive throughout the stomach in some cases, in 33 (15%) of 221 patients. These findings were not included in the primary endpoint, as intestinal metaplasia without visible columnar epithelium is not Barrett's oesophagus. The guidelines for gastric intestinal metaplasia including the cardia were recently reviewed (2019), and UK and US societies suggest that, although the evidence is more scarce than it is for Barrett's oesophagus, surveillance endoscopy should be considered when the gastric intestinal metaplasia is extensive or when there are other factors indicating an increased risk of gastric cancer, such as a family history.^27^, ^28^

Overdiagnosis of cancer is a matter of much debate in the screening community, together with whether short segments (1 cm or less) of Barrett's oesophagus should be considered as such. The TFF3 test is sensitive and detects some short segments of Barrett's oesophagus. Additionally, since this was a pragmatic trial that relied on a coded diagnosis of Barrett's oesophagus, we also identified patients in the usual care group who had short segments of Barrett's oesophagus (1 cm or less in length) and were diagnosed as having the condition, reflecting the variable practice in UK hospitals (appendix p 132). We expect that these patients can be reassured and probably do not require surveillance. This expectation is consistent with the clinical guidelines, which suggest that patients with over 1 cm of salmon-coloured epithelium containing intestinal metaplasia should be monitored.^11^, ^12^ With regard to the primary endpoint analysis, if we use a stringent criterion to diagnose the most clinically significant cases of Barrett's oesophagus (ie those 3 cm or more in length; appendix p 132), four (<1%) of 6388 participants would be diagnosed with Barrett's oesophagus in the usual care group and 46 (1%) of 6834 participants would be diagnosed with Barrett's oesophagus in the intervention group. This result would still show a positive effect of introducing the Cytosponge-TFF3 procedure into clinical care, with an RR of 10·5 (95% CI 3·8–29·4), after accounting for clustering (data not shown).

Further guidance will be required to tailor the follow-up of patients diagnosed via the Cytosponge-TFF3 procedure, depending on their degree of risk of progressing to dysplasia or cancer according to the clinical surveillance guidelines. In the future, we expect that additional biomarkers will distinguish indolent Barrett's oesophagus from Barrett's oesophagus at high risk of progression, so that many patients can be followed up with the Cytosponge-TFF3 procedure, and endoscopy can be reserved for those at a high risk who are likely to require intervention. Identification of risk stratification biomarkers is an ongoing area of research.^29^

In this trial, patients were offered the Cytosponge-TFF3 procedure if they required medication for heartburn symptoms. In many health-care systems, a one-off endoscopy would be considered for these patients given that many require long-term medication (ie, for 3 years or more). The sensitivity and specificity of the Cytosponge-TFF3 procedure have been evaluated previously,^22^ and our trial was not designed to re-evaluate these aspects. However, based on the number who had an endoscopy following a Cytosponge-TFF3 procedure but did not have Barrett's oesophagus or cancer (n=90), and on the number who successfully swallowed the Cytosponge-TFF3 but did not have Barrett's oesophagus or cancer (n=1523), we estimated the specificity of the Cytosponge-TFF3 procedure to detect Barrett's oesophagus, dysplasia or cancer to be 94%. In the future, consideration should be given to the ideal enrichment criteria, which might include a different age cutoff for men compared with women because of the difference in incidence (ie, 87 [69%] of 127 Barrett's oesophagus diagnoses in patients who successfully swallowed the Cytosponge were male), and also the inclusion of other risk factors, such as body-mass index.

Among the strengths of our trial is the real-world implementation of the Cytosponge-TFF3 procedure, including the administration of the device by a nurse in the community setting. The TFF3 test was done in a clinically certified laboratory, and the results were communicated in real time. The use of coding to ascertain diagnoses of Barrett's oesophagus, dysplasia, and cancer ensured equity across both study groups. Since informed consent from individual patients was obtained only for those who underwent the Cytosponge-TFF3 procedure, the use of coding was the only way to ascertain the diagnoses for participants in the usual care group and those in the intervention group who declined the invitation to undergo the Cytosponge-TFF3 procedure.

This trial has some limitations. First, those participants who agreed to undergo the Cytosponge-TFF3 procedure might have had more problematic symptoms than those who did not accept the offer of the procedure. We eliminated this bias by analysing the data of the whole trial as an intention-to-treat analysis. Second, 150 (9%) of 1654 participants still had a low-confidence result after the offer of a repeat test. Work is ongoing to find out how to reduce this outcome. Third, there were slightly more women than men agreeing to undergo the Cytosponge-TFF3 procedure, even though Barrett's oesophagus is more prevalent in men than in women. In future, strategies to encourage men to attend the procedure, and whether to alter the threshold for testing men versus women, should be considered. Finally, variation in the quality of endoscopies was apparent across the 24 hospitals that took part in the study.^30^ We (MDP) did a central review of video images and liaised with hospitals to ensure consistency in reporting. Currently, the TFF3 test requires manual reading by a pathologist trained in analysing these specimens, which are much larger and more cytological in nature than endoscopic biopsies. Work is underway to use deep machine learning to automate the quality control and assist the pathologists in their diagnosis, thus substantially reducing the reporting time and observer bias.

In conclusion, for patients with heartburn-predominant symptoms requiring acid-suppressant therapy for at least 6 months, the Cytosponge-TFF3 procedure is a feasible, safe, and generally acceptable test to administer in the general practice clinic setting. This procedure results in improved detection of Barrett's oesophagus, thus enabling a more proactive approach for the identification and minimally invasive treatment of dysplasia and early cancer. An economic evaluation will establish the effect of this strategy, taking into account the additional number of endoscopies required as a result of the Cytosponge-TFF3 procedure.

## Data sharing

Aggregated data are available for the usual care group and the intervention group, and individual patient-level data are available for patients who consented to the Cytosponge-TFF3 intervention. The trial protocol, statistical analysis plan, and statistical report will be available via the University of Cambridge data repository (https://www.data.cam.ac.uk/repository).
