# Supplementary material for: Cytosponge-trefoil factor 3 versus usual care to identify Barrett's oesophagus in a primary care setting: a multicentre, pragmatic, randomised controlled trial
Source: Lancet. 2020 Aug 1;396(10247):333–44. doi: 10.1016/S0140-6736(20)31099-0 (PMC7408501; doi:10.1016/S0140-6736(20)31099-0)
Supplement: Supplementary appendix [file mmc1.pdf]

THE LANCET

Supplementary appendix

This appendix formed part of the original submission and has been peer reviewed. We post it as supplied by the authors.

Supplement to: Fitzgerald RC, di Pietro M, O'Donovan M, et al. Cytosponge-trefoil factor 3 versus usual care to identify Barrett's oesophagus in a primary care setting: a multicentre, pragmatic, randomised controlled trial. *Lancet* 2020; **396:** 333–44.

Appendices - Cytosponge-trefoil factor 3 versus usual care to identify Barrett's oesophagus in a primary care setting: a prospective, multicentre, pragmatic, randomised controlled trial – Fitzgerald et al, Lancet 2020

Table of Contents

Appendix 1 - Members of the BEST3 trial team and of the data monitoring and trial steering committees	Page 2
Appendix 2 - BEST3 Study Protocol	Page 3
Appendix 3 - MHRA approval to amendment in study design	Page 87
Appendix 4 - BEST3 Statistical Analysis Plan	Page 90
Appendix 5 - Supplementary figures and tables	Page 126

Appendix 1 – Members of the BEST3 trial team and of the data monitoring and trial steering committees

BEST3 Trial Team

Abhay Bagewadi, Abigail Patrick, Achuth Shenoy, Aisling M Redmond, Ajay Muddu, Alex Northrop, Alice Groves, Alice Shiner, Amardeep Heer, Amrit Takhar, Amy Bowles, Andrea Jarman, Angela Wong, Angie Lucas, Anita Gibbons, Anjan Dhar, Anji Curry, Anna Lalonde, Anna Swinburn, Anne Turner, Anne-Marie Lydon, Anthony Gunstone, Arlene Lee, Arul Nambi, Arun Ariyarathenam, Ashley Elden, Ashley Wilson, Balaji Donepudi, Barbara Campbell, Basia Uszycka, Ben Bowers, Ben Coghill, Bruno de Quadros, Calvin Cheah, Carla Bratten, Carly Brown, Chantelle Moorbey, Charles Clisby, Charles Gordon, Chris Schramm, Chris Castle, Chris Newark, Chrissie Norris, Christine A'Court, Claire Graham, Clare Fletcher, Clare Grocott, Colin Rees, Corinne Bakker, Costas Paschalides, Craig Vickery, Damian Schembri, Danielle Morris, Daryl Hagan, David Cronk, David Goddard, David Graham, Dean Phillips, Deeksha Prabhu, Deepak Kejariwal, Dhirendra Garg, Diane Lonsdale, Dianne Butterworth, Donna Clements, Drew Bradman, Duncan Blake, Elizabeth Mather, Ewan O'Farrell, Florian Markowetz, Fran Adams, Francesca Pesola, Gareth Forbes, Gary Taylor, Glenn Collins, Gordon Irvine, Gysbert Fourie, Harriet Doyle, Heather Barnes, Helen Bowyer, Helen Whiting, Ian Beales, Ian Binnian, Ian Bremner, Ian Jennings, Ilona Troiceanu, Ines Modelell, Ingrid Emmerson, Jacobo Ortiz, Jacqueline Lilley, Jacquelyn Harvey, Jacqui Vicars, Jagjit Takhar, James Larcombe, Jan Bornschein, Jehad Aldegather, Jenny Johnson, Jill Ducker, Jo Skinner, Joanne Dash, Joanne Walsh, Jose Miralles, Josephine Ridgway, Julia Ince, Julie Kennedy, Kat Hampson, Kate Milne, Katherine Ellerby, Katherine Priddis, Kathy Rainsbury, Kelly Powell, Kerry Gunner, Krish Ragunath, Kyle Knox, Laura Baseley, Lauren White, Laurence Lovat, Lee Berney, Lindsay Crockett, Lisa Murray, Lisa Westwood, Lisa Chalkley, Loraine Leggett, Louise Dale, Louise Scovell, Lucy Brooks, Lucy Saunders, Lydia Owen, Maria Dilwershah, Marie Baldry, Marie Corcoran, Marie Roy, Mario Macedo, Mark Attah, Mary-Jo Anson, Matt Rutter, Matthew Wallard, Matthew Gaw, Matthew Hunt, Megan Lea-Hagerty, Melchizedek Penacerrada, Michele Bianchi, Michelle Baker-Moffatt, Michelle Czajkowski, Michelle Sleeth, Nick Brewer, Nick Wooding, Nicky Todd, Nicola Millen, Olga Zolle, Orla Whitehead, Patrick Ojechi, Patrick Moore, Paul Banim, Paula Spellar, Pradeep Bhandari, Prashant Kant, Rachel Nixon, Rebecca Russell, Rebekah Roberts, Rene Skule, Richard West, Robin Fox, Ruth Beesley, Ruth Gibbins, Ruth Osborne, S Thiagarajan, Sally Bastiman, Samantha Warburton, Samir Pai, Sarah Leith-Russell, Sarah Utting, Sarah Watson, Sarah Wytrykowski, Satish Singh, Shalini Malhotra, Sharon Woods, Shaun Conway, Sherrie Mateer, Shona Macrae, Shruti Singh, Simona Fourie, Siobhan Campbell, Siobhan Parslow-Williams, Sonica Goel, Stephen Dellar, Stephen Jones, Steve Knight, Stuart Mackay-Thomas, Stuti Mukherjee, Sue Allen, Suzanne Henry, Tara Evans, Theresa Leighton, Tim Bray, Tom Shackleton, Vanaja Santosh, Vicki Glover, Vijay Chandraraj, Will Elson, William Briggs, Zoe Barron, Zohrah Khan

The Trial was managed by the Cancer Research UK & King's College London Cancer Prevention Trials Unit.

Independent data monitoring committee: Stephen Attwood (Chair), Max Parmar, Brendan Delaney.

Independent trial steering committee: John de Caestecker (Chair), Wendy Atkin (in memoriam), Allan Hackshaw, Charles van Heyningen (patient representative), Tim Underwood, Nick Roberts (patient representative).

Appendix 2: BEST3 Study Protocol

Protocol

Randomised controlled trial comparing the Cytosponge™-TFF3 test with usual care to facilitate the diagnosis of oesophageal pre-cancer in primary care

Barrett's oESophagus Trial 3 (BEST3): A trial of a new GP-based test for patients with heartburn symptoms

This protocol has regard for the HRA guidance and order of content

<u>**Research reference numbers**</u>

**Protocol version and date**	**Version 3.1 dated 10 June 2019**
**REC reference:**	**16-EE-0546**
**IRAS Number:**	**210292**
**MHRA reference**	**CI/2016/0057**
**ISRCTN Number:**	**ISRCTN68382401**
**Sponsor reference:**	**A093969**
**Funder reference:**	**C14478/A21047**

Signature page

The undersigned confirm that the following protocol has been agreed and accepted and that the Chief Investigator agrees to conduct the trial in compliance with the approved protocol and will adhere to the principles outlined in the Medical Devices Directive **(**93/42/EEC), GCP guidelines, the Sponsors' SOPs, and other regulatory requirements as amended.

I agree to ensure that the confidential information contained in this document will not be used for any other purpose other than the evaluation or conduct of the clinical investigation without the prior written consent of the Sponsor.

I also confirm that I will make the findings of the study publicly available through publication or other dissemination tools without any unnecessary delay and that an honest accurate and transparent account of the study will be given; and that any discrepancies from the study as planned in this protocol will be explained.

Chief Investigator
…………………………………………………………Date..10 June 2019…
Name (Please print)
………………………… Prof Rebecca Fitzgerald…………………………

Statistician
………………………………………………………. Date…10 June 2019…
Name (Please print)
………………………………Prof Peter Sasieni…………………………….

KEY TRIAL CONTACTS

Chief Investigator	Rebecca C Fitzgerald, Professor of Cancer Prevention, MRC Cancer Unit, University of Cambridge and Honorary Consultant Gastroenterologist, Cambridge University Hospitals NHS Foundation Trust rcf29@mrc-cu.cam.ac.uk Tel: 01223 763240
Clinical Trial Design and Statistics and Co-Investigator	Peter Sasieni, Professor of Cancer Prevention, Guy's Cancer Centre and Director of CRUK & KCL CPTU, King's College London. peter.sasieni@kcl.ac.uk, Tel: 020 7188 4484
Primary Care - Collaborator	Fiona Walter, GP & Principal Researcher in Primary Care Cancer Research, Department of Public Health and Primary Care, University of Cambridge
Primary Care - Collaborator	Greg Rubin, Professor of General Practice and Primary Care, University of Newcastle
Health Economics - Collaborator	Stephen Morris, Professor of Health Economics, University College London
Epidemiology	Judith Offman, King's College London, Faculty of Life Sciences & Medicine, School of Cancer & Pharmaceutical Sciences, Cancer Prevention Group, Innovation Hub, Guys Cancer Centre, Guys Hospital.
Health economics	Nick Swart, Research Associate, University College London
Health Psychology	Sam Smith, Research Fellow, University of Leeds
Funder - CRUK	Cancer Research UK, Strategy & Research Funding CRUK, Angel Building, 407 St John Street, London, EC1V 4AD prc@cancer.org.uk Tel 020 3469 5232<br>Primary funders, Research Costs (Part A)
Funder – National Institute of Health Research	Service support costs and Research Costs (Part B)
Funder - NHS Commissioners	Financial support via NHS Treatment Costs
Funder - Medtronic	Global Market Development Medtronic, GI Solutions 540 Oakmead Parkway, Sunnyvale, CA 94085 USA<br>terry.s.davison@medtronic.com Tel: 408 328 7352
Clinical Trials Unit	CRUK & KCL Cancer Prevention Trials Unit, King's College London, Cancer Prevention Group, Innovation Hub, Guys Cancer Centre, Guys Hospital, Great Maze Pond, London SE1 9RT, UK

	cptu@kcl.ac.uk Tel 020 784 89705
Joint Sponsor	Dr Jo Martindale, Assistant Director, Research Operations Office, School of Clinical Medicine, University of Cambridge, Greenwich House, Madingley Rd, Cambridge researchgovernance@medschl.cam.ac.uk Tel: 01223 765145
Joint Sponsor	Stephen Kelleher, R&D Manager, Cambridge University Hospitals NHS Foundation Trust, Box 277, Addenbrookes Hospital, Hills Rd, Cambridge CB2 0QQ research@addenbrookes.nhs.uk<br>Tel: 01223 348490
Trial Steering Committee	c/o CRUK & KCL Cancer Prevention Trials Unit, King's College London, Cancer Prevention Group, Innovation Hub, Guys Cancer Centre, Guys Hospital, Great Maze Pond, London SE1 9RT, UK<br>cptu@kcl.ac.uk Tel 020 784 89705
Data Monitoring Committee	c/o CRUK & KCL Cancer Prevention Trials Unit, King's College London, Cancer Prevention Group, Innovation Hub, Guys Cancer Centre, Guys Hospital, Great Maze Pond, London SE1 9RT, UK<br>cptu@kcl.ac.uk Tel 020 784 89705

TRIAL SUMMARY

Trial title	Barrett's oESophagus Trial 3 (BEST3): Randomised controlled trial comparing the Cytosponge™-TFF3 test with usual care to facilitate the diagnosis of oesophageal pre-cancer in primary care	
Internal ref. no. (or short title)	Barrett's oESophagus Trial 3 (BEST3)	
Trial design	Randomised controlled trial using cluster and individual randomisation	
Trial participants	- Male and female<br>- Aged 50 and above<br>- With at least 6 months of prescriptions for either a proton pump inhibitor or an H2 receptor antagonist in the last year<br>- Who have not had an endoscopy in the last 5 years<br>- Who are not on a regular prescription of NSAIDs	
Planned sample size	8,988 patients: 4,494 patients in each arm with 50% expected to accept the Cytosponge™ test	
Revised sample size (Milestone 1 review)	Approximately 15,656 patients: ~7,828 patients in each arm with 27% expected to accept the Cytosponge™ test	
Current sample size	Cluster randomised group: 7,859 patients<br>Patient-level randomised group: 4,641 patients *(adjusted according to size of cluster randomised group)*<br>Total: 12,500 patients, approximately 50% per arm	
Trial duration	Set-up period: 3 months<br>GP recruitment: 23 months + 3 months patient recruitment at last practice<br>Follow-up: Average 12 months (9-15 month range)<br>Analysis: 3 months<br>Total: 44 months<br><br>Research endoscopies: patients invited in practices recruited in first 9 months	
Objectives	**Objective**	**Outcome measure**
Primary	To compare histologically confirmed Barrett's oEsophagus (BE) diagnosis between intervention and the control, i.e. usual care, arms	BE diagnosis within follow-up period
Secondary	Multiple	Multiple

Investigational medical device	Barrett's oEsophagus Cytosponge™ test kit, Class I, single-use, non-sterile, non CE-marked
Long-term follow up duration (Future research)	10 years.<br>• Future research: Anonymous data follow-up including for health episode statistics, cancer incidence and mortality<br>• Future research: Patient-level follow up (via datasets including those maintained by NHS Digital, NHS Health and Social Care Information Centre (HSCIC), ONS and Public Health England for consented patients)

Funding and support in kind

FUNDER(S)	FINANCIAL AND NON FINANCIAL SUPPORT PROVIDED
CRUK	Primary funders, Research costs (Part A)
NIHR	Service support costs, Research costs (Part B)
NHS Commissioners	Excess treatment costs
Medtronic	Device and antibody supply

Role of sponsor and funder

Cambridge University Hospitals NHS Foundation Trust and the University of Cambridge will act as joint sponsors for this Trial. These organisations will retain overall responsibility for the design, management and conduct of study implementation in line with Cancer Research UK's grant funding conditions. Access to data and Intellectual Property Rights will be governed by these grant conditions.

Trials management will be conducted by the CRUK & KCL Cancer Prevention Trials Unit, who will manage the trial implementation on behalf of the Sponsors. Medtronic will have access to trial data as licensee of the Cytosponge™ technology from the Medical Research Council. The Trial database, including the housing of person-identifiable data, will be implemented from Queen Mary University of London.

Trial Committees
Data Monitoring Committee
An independent Data Monitoring Committee (DMC) will review trial data and advise the sponsors (directly or indirectly) on the future management of the Trial. Its membership will include two independent clinicians and an independent statistician.

Trial Steering Committee (TSC)
The Trial Steering Committee is to provide overall supervision for the Trial on behalf of the trial sponsors and trial funders and to ensure that the Trial complies with relevant regulatory, legal and best practice standards.

Trial Management Group (TMG)

The Trial Management Group is responsible for the day-to-day running and management of the Trial. The TMG will oversee the progress of the Trial for the day-to-day implementation and act on the advice of the TSC. Amongst its members are the lead investigators (clinical and non-clinical), trial co-ordinators, and staff from King's College London (KCL).

Protocol contributors

A range of stakeholders have contributed to the development of this protocol including academic and research management staff at the University of Cambridge, Cambridge University Hospitals NHS Foundation Trust, the Centre for Cancer Prevention at the Wolfson Institute of Preventive Medicine, Queen Mary University of London, King's College London including CRUK KCL Cancer Prevention Trials Unit team members, Newcastle University, University College London, Medical Research Council Biostatistics Unit and research delivery and management staff at the National Institute of Health Research. Protocol design has taken account of the view of patients and the public via several rounds of reviews, focus groups and other events.

The Sponsors are solely responsible for study design, conduct, data analysis and interpretation, manuscript writing, and dissemination of results. They will have the final decision in all matters relating to design and implementation and in line with funding conditions.

**KEY WORDS:**	Heartburn, acid reflux, primary care, Barrett's oesophagus, early detection, oesophageal cancer

Table of contents

TRIAL SUMMARY **5**
1 Trial flowchart **11**
2 Background **12**
2.1 Clinical need 12
2.2 Cytosponge^{TM} diagnostic test for Barrett's oESophagus (BE) 12
3 Rationale **14**
3.1 Assessment and management of risk 14
4 Objectives and primary measures/endpoints **15**
4.1 Primary objective and hypothesis 15
4.2 Secondary objectives 15
4.3 Outcome measures/endpoints 15
4.4 Objectives and study endpoints 15
5 Trial design **21**
6 Study setting **22**
7 Eligibility criteria **22**
7.1 Eligibility criteria for the BEST3 data collection 22
7.2 Eligibility criteria for Cytosponge^{TM}-TFF3 Test 23
7.3 Eligibility criteria for research endoscopies (both arms excluding participants with positive result) 24
8 Trial procedures **24**
8.1 Recruitment 24
8.2 Patient identification 25
8.3 Consent procedures 25
8.3.1 Consent at practice level (Opt in) 25
8.3.2 Introductory letter provided to patients about use of anonymous data (BEST3 Data collection) 25
8.3.3 Written consent for BEST3 Intervention study (Opt in written consent) 25
8.4 Practice randomisation (Cluster) 30
8.4.1 BEST3 Introductory letter 30
8.4.2 Anonymised data collection for BEST3 Trial 32
8.5 Patient randomisation (Individual) 33
8.6 BEST3 Intervention: Cytosponge^{TM}-TFF3 test 33
8.6.1 Patient invitation to BEST3 Intervention 33
8.6.2 Cytosponge^{TM} clinic procedure 34
8.6.3 Cytosponge^{TM}-TFF3 results 35
8.7 Endoscopies 35
8.7.1 Invitation for endoscopies - Intervention arm: Patients with positive result 35
8.7.2 Invitation for research endoscopies - Patients who do not require diagnostic endoscopy 37

8.7.3 Endoscopy procedures: Patients with positive results 38
8.7.4 All other participants 38
8.8 Acceptability measures- Intervention group only 39
8.8.1 Patient acceptability measures 39
8.9 12-month (Average) follow-up 40
8.10 Long-term follow-up 40
8.11 Intervention acceptability for patients and health care professionals 40
8.12 Withdrawal criteria 42
8.13 End of Trial 43
9 Investigational medical device 44
9.1 Name and description of device 44
9.2 Legal status of the device 45
9.3 Technical design 45
9.4 Device storage and supply 45
9.5 Schedule of use 45
9.6 Interaction with other therapies 45
10 Safety reporting and medical device vigilance 46
10.1 Scope 48
10.2 Mode of reporting 48
10.3 Recording of SA(D)Es 49
10.4 Electronic management of SA(D)Es 49
10.5 Device deficiencies 50
10.6 Responsibilities 51
11 Storage and analysis of samples 54
11.1 Laboratories 56
12 Statistics and data analysis 57
12.1 Sample size and power calculations 57
12.2 Timescale, potential challenges and milestones 58
12.3 Statistical analysis plan 61
12.4 Economic evaluation 63
13 Data handling 64
13.1 Anonymous GP datasets 64
13.2 Case report form 66
13.3 Record retention and archiving 67
14 Trial Committees 67
15 Monitoring, audit and inspection 68
16 Ethical and regulatory considerations 68
16.1 Health Research Authority (HRA) and Research Ethics Committee (REC) approvals 68
16.2 Notification of No Objection from MHRA 68

16.3 Peer review 68
16.4 Regulatory compliance 69
16.5 Protocol compliance 69
16.6 Data protection and patient confidentiality 69
16.7 Financial and other competing interests 70
16.8 Insurance and indemnity arrangements 70
16.9 Amendments 70
16.10 Access to the final trial dataset 71
17 Public and Patient Involvement 71
17.1 Cytosponge^{TM} acceptability study 71
17.2 Involvement of PPI representatives in the study design 71
17.3 Further involvement of PPI representatives 71
18 Dissemination policy 71
18.1 Sharing of datasets 71
18.2 Discoverability of dataset 72
18.3 IP considerations 73
18.4 Authorship guidelines 73
19 References 73
Appendix 1: List of abbreviations 74
Appendix 2: Risk Assessment 77
Appendix 3: Table of procedures 78
Appendix 4: Safety Reporting Flow Chart 79
Appendix 5: Amendment History 80

1 Trial flowchart

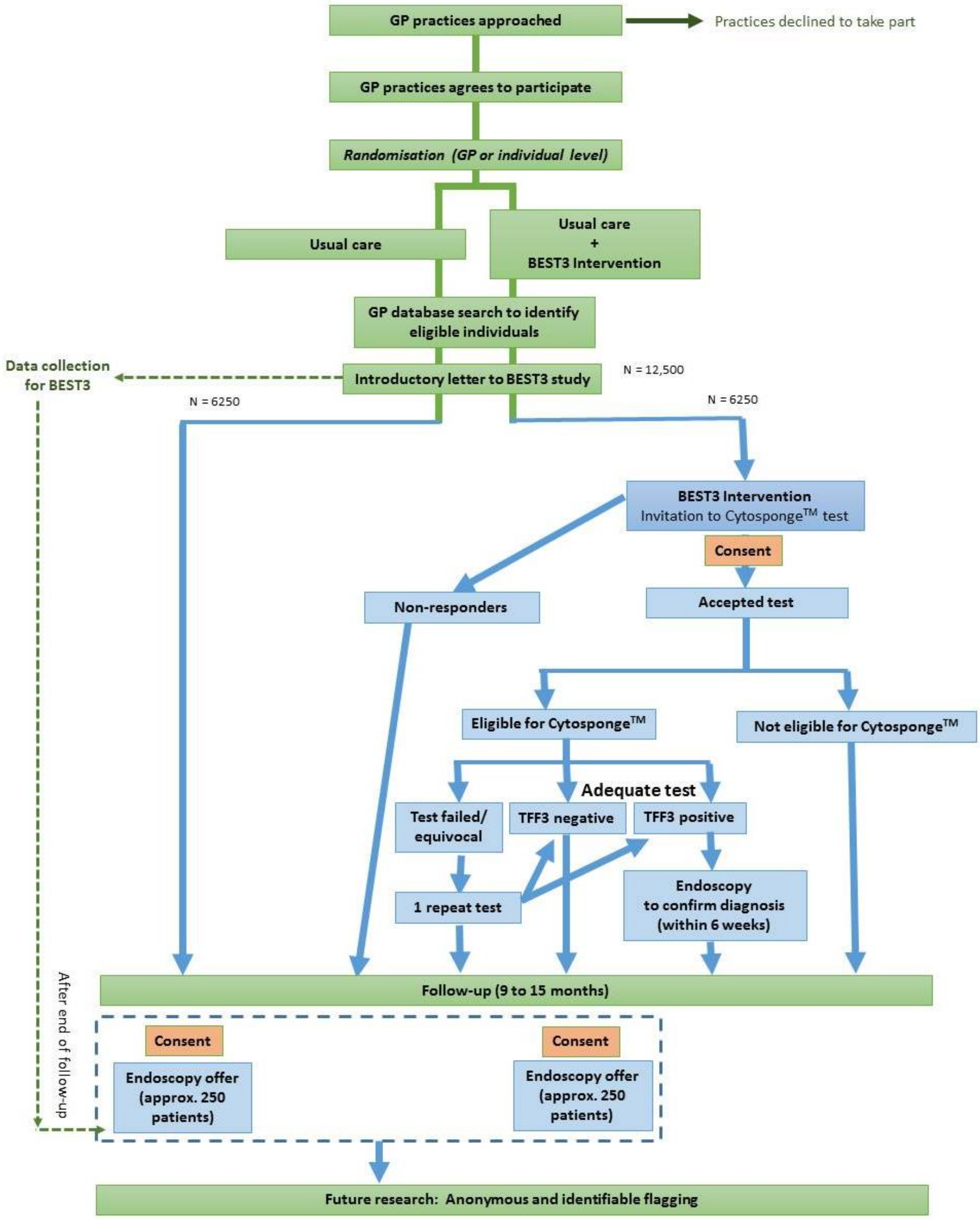

Figure 1: Trial design schematic

2 Background

2.1 Clinical need

OEsophageal adenocarcinoma (EAC) is a cancer whose incidence has increased 6-fold since the 1990s and carries a dismal prognosis (13% 5-year survival) despite advances in neo-adjuvant therapy and surgery [1]. This cancer has been highlighted as a public health concern in the UK [2]. Clinical guidelines have focused on urgent referral for those with alarm symptoms, and routine referral for those with symptoms that persist despite recommended lifestyle and pharmacological management strategies [3]. Nevertheless, General Practice (GP) referral rates vary widely, and low endoscopy referral rates have been linked with poor outcomes from oesophageal cancer [4].

3 to 6% of individuals with reflux predominant symptoms may have Barrett's oEsophagus (BE), which is the precursor lesion to EAC, but only 20 to 25% of patients with BE are diagnosed [5]. It is estimated that the burden of EAC could be reduced by up to 50% as a result of increasing the proportion of individuals with reflux predominant symptoms who are investigated [6]. This is a formidable task since dyspepsia and gastro-oesophageal reflux disease (GERD) affect between 5%-20% of the population [7] and account for up to 10% of general practitioner (GP) consultations in the UK. Recent national awareness campaigns are likely to increase these consultations further [8]. In view of the scale of the problem and the costs (psychological and financial) of investigation, any new strategy needs to be carefully evaluated. As highlighted by the Chief Medical Officer, Sir Liam Donaldson, in his 2008 report, there is a need for a safe, minimally invasive, affordable test applicable to the primary care setting to diagnose BE [2].

Endoscopic treatment of BE, which progresses through dysplastic and superficially invasive stages, offers the opportunity to prevent the development of EAC. Indeed, endoscopic treatment is now recommended for patients with low and high grade dysplasia following new Randomised Controlled Trial evidence [9].

In summary, NICE guidelines refer only a small proportion of patients with reflux for endoscopy. Thus the vast majority of patients with reflux would not be seen in endoscopy. This proposal addresses an unmet need in those patients. By identifying and referring to endoscopy those most likely to have BE, therefore at higher risk of cancer progression, it should be possible to reduce mortality from EAC in patients with reflux who would not otherwise receive endoscopy.

2.2 Cytosponge™ diagnostic test for Barrett's oESophagus (BE)

A non-endoscopic diagnostic modality for BE has been developed which involves a device called the Cytosponge™ combined with molecular biomarker Trefoil Factor 3 (TFF3) [10].

The Cytosponge™ consists of a Class I, non-CE marked 3cm diameter, polyester, medical grade sphere on a string, compressed within a gelatine capsule. The capsule is swallowed whilst holding onto the string. After 5 minutes, the gelatine capsule has dissolved allowing the sphere to expand. Using the string the sphere is pulled from the stomach to the oesophagus and mouth thus collecting cells from the whole of the BE segment [11], as well as from the squamous oesophagus and oropharynx. The sample is then put into a preservative. The sample can then be processed and assessed for the presence of BE via TFF3. The device is not CE-marked and has been used in 2

previous research studies, the most recent of which is the BEST2 Trial involving 1500 participants (CI/2010/0040).

Our long-term vision is for the Cytosponge™-TFF3 technology to be adopted as a triage test within the standard primary care clinical pathway for patients on long-term treatment with acid suppressants who do not fulfil the referral criteria for endoscopy. This strategy will increase the proportion of patients diagnosed with BE, and in turn allow for endoscopic therapy and monitoring for those at greatest risk of EAC.

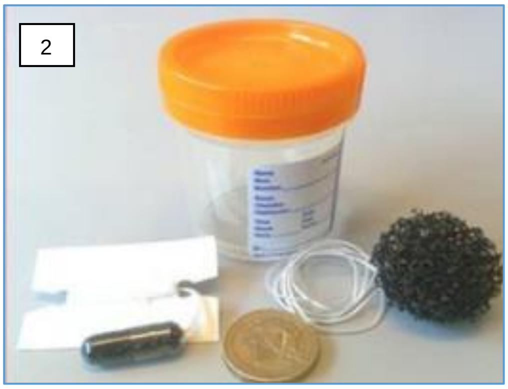

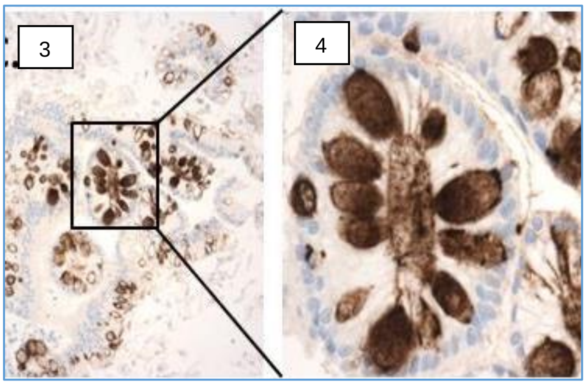

Figure 2: Cytosponge™ and Figures 3-4: Representative pictures of positive TFF3 staining in a sample from a patient with BE (x100 and x400 magnification)

A series of 4 clinical studies (outlined in Table 1) have demonstrated:

- The intervention can be applied to primary care: Feasibility study conducted in 504 patients in 11 general practices [12]
- The intervention is safe: Cytosponge™ administered to 2000 patients and no serious adverse events attributed to the device [10, 12-15]
- The Cytosponge™-TFF3 test is accurate for diagnosing BE: regardless of patient cohort or study setting. A commercially-developed device used in CASE1 has improved sensitivity further (Table 1).

Table 1: Studies summary, sensitivity and specificity of the Cytosponge™ -TFF3 test per segment length

Study Ref n#	Publication Year	Study type	Setting	BE length	Sensitivity % (95% CI)	Specificity % (95% CI)
**Pilot [14]**	2008	Cohort	2ndary care	≥C1	78.0 (64.0-89.0)	94.0 (87.0-98.0)
**BEST1 [12]**	2010	Prospective	1ary care	≥C1	73.3 (44.9-92.2)	93.8 (91.3-95.8)
				≥C2	90.0 (55.5-99.7)	93.5 (90.9-95.5)
**BEST2 [13]**	2014	Case:control	2ndary care	≥C1	79.5(75.9-82.9)	92.4 (89.5-94.7)
				≥C2	83.9(80.0-87.3)	
				≥C3	87.2(83.0-90.6)	
**CASE1 [15]**	2015	Cohort	2ndary care	≥C1 or ≥M3<br>≥C3	95.4 (86.9-98.9)<br>96.8 (83.7-99.5)	N/A

- The Cytosponge™ is acceptable to patients: mean score of 6.0 (95%CI 5.0-8.0) on a visual analogue scale from 0 (worst experience) to 10 (best experience) [10, 12-15]
- Transferability to the NHS: 27 nurses were trained with a single training session in 11 sites. Sample processing run in an NHS pathology laboratory [2, 12]
- The cost-effectiveness of the test in comparison to the usual care: micro-simulation model suggested a gain of 0.015 QALYs and an ICER of $15,700 per QALY for Cytosponge™ versus endoscopic diagnosis of BE followed by endoscopic treatment [16]

3 Rationale

In light of the unmet need set out above, the BEST3 Trial is now needed to:

- Demonstrate that the invitation to the Cytosponge™ -TFF3 test leads to an increase in the number of patients diagnosed with BE compared to the usual clinical care pathway in primary care. An increase in diagnosis will not only depend on accuracy but also acceptability and therefore uptake of the test.
- Gain an in-depth understanding of the health economics of the Cytosponge™ -TFF3 test in patient on long-term treatment with acid suppressants as well as the economics for the projected reduction of EAC-related mortality.

3.1 Assessment and management of risk

The Cytosponge™-TFF3 test may directly benefit patients as they will be tested for BE even if they would not have been referred for an endoscopy as part of their routine care. Any -positive results will be confirmed through an endoscopy.

The Cytosponge™-TFF3 test itself has been shown to be very low risk in relevant clinical studies to date. The inclusion and exclusion criteria aim to reduce the risk to a minimum by careful management of patients with bleeding tendencies through medication (warfarin, etc.) as well as other contra-indicated clinical conditions. If detachment did occur (<1% risk) then the patient would need to be endoscoped at their local hospital. Where there are obvious indications of bleeding [1], the patient would be assessed for the need for endoscopy according to current clinical practice.

In addition to the patients who receive a positive test result, approximately 250 of the patients in each arm (who have not had endoscopy since the start of the Trial) will be randomly selected to be invited for an endoscopy at approximately 12 months. This proportion may be adjusted upwards depending on project's progress to ensure a minimum number of participants are invited. The risks for a diagnostic endoscopy are extremely low (<1:1000 perforation and haemorrhage risk). The endoscopists will use routine care protocols for reporting BE and benign conditions.

This Trial is categorised as Type A = No higher than the risk of standard medical care (See Appendix 1)

[1] Such as weakness, change on pallour, dizziness, fainting, haematemesis / vomiting blood, black tarry stool

4 Objectives and primary measures/endpoints

4.1 Primary objective and hypothesis

The Trial's primary objective is to compare histologically-confirmed BE diagnosis between the intervention and the control, i.e. usual care, arms in all patients entered into the study.

The Trial's hypothesis is that the introduction of the Cytosponge™-TFF3 test in primary care offers a cost-effective and acceptable method to triage patients on long-term treatment with acid suppressants at increased risk of EAC to endoscopy, without unduly burdening the patients or the endoscopy services. See Section 12 for summary of plan for statistical analysis and the standalone document for full details.

The study's endpoint will be measured via coded search to ensure that the depth of data review is equitable in both study arms. Practices will run a search using the codes used in everyday practice to identify Upper GI referrals/OGDs, BE and EAC. In practices not reviewing all patients in depth, results from the coded search will be integrated with results from a manual review on a sample of randomly-selected participants' records and, where applicable, performed in an equal number of usual care and intervention GP practices. For cluster-randomised practices, it should be ensured that randomly-selected case note reviews are in an approximately equal number in the two study arms. A manual review will also be performed on all those records identified by the automated search. Finally, when possible, an automated linkage method will allow us to add any additional information coming from participants' anonymised records in secondary care using encrypted NHS numbers.

As required, a central review on endoscopic images and histology samples by the Trial Endoscopist and Pathologist will also be undertaken to assess the quality of BE diagnosis in BEST3 patients who received a trial endoscopy following a positive Cytosponge™ test result.

4.2 Secondary objectives

There are multiple secondary objectives of the Trial including:

- To evaluate the cost of the Cytosponge™ -TFF3 test versus usual care
- To evaluate the cost-effectiveness of the Cytosponge™ TFF3 test versus usual care

Secondary trial endpoint data will be collected on a randomly-selected subset of the total trial population. Further secondary objectives are set out in section 4.4.

4.3 Outcome measures/endpoints

The main outcome measure for the primary objective is:

- BE diagnosis within approximately 12 months of joining the study (depending on length of follow-up period – see Section 12.2.3), excluding BE found on research endoscopy after the end of follow-up.

Outcome measures for the secondary objectives are listed in section 4.4.

4.4 Objectives and study endpoints

A table with the study endpoints and primary and secondary objectives can be found below (Table 2).

Table 2. BEST3 Trial objectives and endpoints

Primary objectives	Endpoint	Usual care arm	Intervention arm
1. To compare histologically-confirmed BE diagnosis between intervention and the control, i.e. usual care, arms in all patients entered into the study	BE diagnosis within approximately 12 months of joining the study (depending on length of follow-up period for practice), excluding BE found on research endoscopy after the end of follow-up. Number of BE cases diagnosed by the Cytosponge™ with also depend on uptake of the offer to have the test.	Anonymised partly patient-level and partly aggregated data (by sex and age) from<br>- GP databases<br>- Confirmed by Upper GI endoscopy (biopsy result) as recorded in the GP record before end of follow-up period (automatic extraction + manual extraction where required)<br>- Secondary care records (when possible)<br><br>**Endpoint will be measured via coded search and integrated with a random manual review and NHS number linkage between primary and secondary care to ensure depth of data review equitable in both study arms**	Anonymised partly patient-level and partly aggregated data (by sex and age) from<br>- GP databases<br>- Confirmed by Upper GI endoscopy (biopsy result) as recorded in the GP record before end of follow-up period (automatic extraction + manual extraction where required)<br>In addition, for patients with Cytosponge™ -TFF3 test:<br>- Endoscopy record and pathology results<br>- Secondary care records (when possible)<br><br>**Endpoint will be measured via coded search and integrated with a random manual review and NHS number linkage between primary and secondary care to ensure depth of data review equitable in both study arms**

Secondary objectives	Endpoints	Usual care arm	Intervention arm
(i) To evaluate the cost of the Cytosponge™ -TFF3 test versus usual care<br>(ii) To evaluate the cost-effectiveness of the Cytosponge™ TFF3 test versus usual care	(i) Mean cost per patient receiving the Cytosponge™ -TFF3 test versus usual care. Costs to include costs of diagnosis using the Cytosponge™ -TFF3 test, endoscopies and biopsies, endotherapy, oesophagectomy, medications, and follow-up in primary and secondary care<br>(ii) Incremental cost per QALY gained of the Cytosponge™ TFF3 test versus usual care	(i) Volume of resource use (endoscopies and biopsies, endotherapy, oesophagectomy, medications, and follow-up in primary and secondary care) from patient records<br>Unit costs (of each item of resource use) from published sources<br>(ii) Calculation of incremental cost per QALY gained to be based on a pre-existing model, supplemented with new data from the Trial.	(i) Volume of resource use (Cytosponges™, endoscopies and biopsies, endotherapy, oesophagectomy, medications, and follow-up in primary and secondary care) from patient records<br>Unit costs (of each item of resource use) from published sources<br>(ii) Calculation of incremental cost per QALY gained to be based on a pre-existing model, supplemented with new data from the trial.

Secondary objectives	Endpoints	Usual care arm	Intervention arm
To assess the diagnostic accuracy of the Cytosponge™ in primary care	Positive Predictive Value (PPV), Negative Predictive Value (NPV) in relation to the length of BE	N/A	- PPV: proportion of TFF3 positive results confirmed to have BE by endoscopy<br>- NPV: proportion of negative Cytosponge™ cases confirmed to not have BE by endoscopy (~250 invited to research endoscopy)
To assess diagnostic performance of Cytosponge™ in detecting severity for BE	Score of BE severity based on BE biopsy results	N/A	Endoscopy reports for participants
To assess the ability of the Cytosponge™ to detect intestinal metaplasia (IM) of the gastric cardia	IM detection by gastric biopsy in TFF3 positive patients without BE	N/A	Endoscopy and pathology reports
To report on the sampling adequacy	Inadequacy rate (same as BEST1 and BEST2)	N/A	CRF to capture:<br>- Sample sufficient to generate result<br>- Proportion of Cytosponge™ samples with <5 and <1 columnar cells (minimal standard)
To confirm the endoscopy referral rate in the intervention arm	Proportion positive out of all adequate TFF3 tests and out of all patients swallowing a Cytosponge™ at least once	N/A	Cytosponge™ -TFF3 test results
To report on patient acceptability for Cytosponge™	(i) Willingness: proportion of patients offered Cytosponge™ who accept<br>(ii) Cytosponge™ swallowing failures<br>(iii) Increased and decreased cancer worry due to procedure and results<br>(iv) Long term emotional or physical harm caused by procedure<br>(v) Test experience<br>(vi) Willingness to have repeat procedure	N/A	(i) Number of patients invited vs those consenting to Cytosponge™ -TFF3 test<br>(ii) Number of patients who fail to swallow and number of attempts<br>Acceptability measures at baseline:<br>(iii) STAI-6<br>(iv) Perceived risk of oesophageal cancer<br><br>Acceptability measures at day 7-14:

Secondary objectives	Endpoints	Usual care arm	Intervention arm
			(iii) perceived risk of oesophageal cancer<br>(iv) STAI-6<br>(v) a visual analogue scale to rate experience<br>(v-vi) the Inventory to Assess Patient Satisfaction,<br>(iii – v) up to 30 qualitative patient interviews
To assess physician/nurse acceptability of the Cytosponge™	Experience and acceptability of Cytosponge™: administration, skills, reliability, side effects, user information	N/A	Qualitative interviews of clinical staff
To report on the safety of the Cytosponge™ in primary care	Any ADE/ARs reported by patients up to 7 days post swallowing	N/A	Contact card given in case of ADE/SADE and 7-day telephone call
(i) To understand how much BE is missed in current management of patients<br>(ii) To compare undiagnosed BE in general population vs those who have been tested with Cytosponge™ -TFF3	- BE during follow-up period<br>- PPV for endoscopy referral, i.e. Cytosponge™ vs current GP criteria for referring for an endoscopy to look for BE	Research endoscopy invites for 250 patients not requiring a clinically indicated endoscopy in time period of the study (see Section 8.7.2)	Confirmatory endoscopy for patients with positive result and endoscopy findings from patients with negative result who accept research endoscopy after the end of their follow-up (see Section 8.7.2)
To assess prevalence of benign oesophageal conditions	Prevalence of oesophageal conditions aside from BE in primary care population consulting with reflux symptoms	Endoscopy findings in approximately 250 patients invited to a research endoscopy (see Section 8.7.2)	Endoscopy findings in approximately 250 patients invited to a research endoscopy and on Cytosponge™ test (via pathology assessment) (see Section 8.7.2)
Epidemiology:<br>(i + ii) To confirm the prevalence (and incidence) of BE in both arms<br>(iii) To confirm the prevalence (and incidence) of OC	(i) Diagnosis of BE<br>(ii) Diagnosis BE with dysplasia<br>(iii) Diagnosis of OC + stage at diagnosis<br>(iv) Diagnosis of cancer of the gastric cardia + stage at diagnosis<br>(v) Percentage of expected reduction in EAC mortality based on prevalence of BE if Cytosponge™ test introduced	(i-iv) Part-aggregated data from GP databases (following coded search, random manual review and NHS number linkage)<br>(i-iv) Endoscopy data from 250 endoscopy invites offered after end of follow-up (see Section 8.7.2)	(i-iv) Part-aggregated data from GP databases (following coded search, random manual review and NHS number linkage)<br>(i-iv) Endoscopy data from 250 endoscopy invites offered after end of follow-up (see Section 8.7.2)<br>(i-iv) Cytosponge™ patients:

Secondary objectives	Endpoints	Usual care arm	Intervention arm
diagnosis (by stage) in both arms<br>(iv) To confirm the prevalence (and incidence) diagnosis of IM and cancers of the gastric cardia (by stage) in both arms<br>(v) To produce model to predict the reduction in EAC related mortality from this strategy			- Cytosponge™ findings<br>- Endoscopy findings
Approximately 500 endoscopy invitations across both arms:<br>(i) Acceptability of endoscopy<br>(ii) Perceptions around CytospongeP™P use and reliability<br>(iii) Number of BE diagnoses at the end of follow-up period after negative Cytosponge™ tests	(i) Comparisons between acceptance of invitation to endoscopy compared to Cytosponge™ test<br>(ii) Proportion of patients with Cytosponge™ test who take up invitation to endoscopy<br>(iii) Number of BE diagnosis in negative Cytosponge™ patients in intervention arm	(i) uptake of invitation to endoscopy	(i) uptake of invitation to endoscopy for all participants who have not received the Cytosponge™ (excluding ineligible patient and non-attendees for Cytosponge™)<br>(i) Cytosponge™ test invitation uptake<br>(ii) Endoscopy uptake amongst patients with previous Cytosponge™ test<br>(iii) BE diagnosis amongst patients with previous negative Cytosponge™ test

Longer-term objectives	Endpoints	Usual care arm	Intervention arm
Epidemiology<br>For up to 10 years, to confirm the prevalence (and incidence):<br>(i + ii) of BE in both arms<br>(iii) of OC diagnosis (by stage) in both arms<br>(iv) of cancers of the gastric cardia (by stage) in both arms<br>(iv)To undertake modelling to predict the reduction in EAC related mortality from this strategy	(i) Diagnosis of BE<br>(ii) Diagnosis BE with dysplasia<br>(iii) Diagnosis of OC + stage at diagnosis<br>(iv) Diagnosis of cancer of the gastric cardia + stage at diagnosis<br>(v) Percentage of expected reduction in EAC mortality based on prevalence of BE if Cytosponge™ test introduced	(i-iv) Anonymised data from cancer registry flagging- conducted anonymously via novel encryption method<br>(v) based on BE prevalence, prevalence of BE with dysplasia, flagging with the cancer registry, ONS and HES datasets	(i-iv) Anonymised data from cancer registry flagging- conducted anonymously via novel encryption method<br>(v) based on BE prevalence, prevalence of BE with dysplasia, flagging with the cancer registry, ONS and HES datasets
Research and Development (including in future studies)	Genetic and biochemical risk factors for disease progression (germline and somatic variants and other biomarkers) including targeted, exome level and whole genome sequencing.	250 patients who have been invited to a research endoscopy (see Section 8.7.2) - surplus material from biopsies	- Surplus Cytosponge™ material<br>- Saliva samples (for TFF3 positive patients only)<br>- Surplus endoscopy biopsies

5 Trial design

The Trial is a pragmatic randomised controlled trial to evaluate whether an invitation to the Cytosponge™-TFF3 test will increase the number of BE diagnoses among patients on long-term treatment with acid suppressants[2] not meeting guidelines for urgent referral (Figure 1). Analysis of a CPRD cohort has shown that patients with at least 6 months of prescriptions for an acid suppressant during one year had the highest rates of BE, and would therefore benefit the most from the Cytosponge™-TFF3 test. Furthermore, the majority of BE patients in this cohort had received at least 6 months of PPI/H2RA prescriptions prior to their BE diagnosis. As patients on repeat prescription obtain their medication from their pharmacy and not during an appointment with their GP, this group of patients will be most comprehensively identified and invited based on the GP database.

Up to 150 practices will be randomised as the project requires. Cluster randomisation practices will be randomised on a 1:1 basis to either the intervention or control arm; whereas for individual randomisation, a 1:1 assignment will be performed on individual patients by practice staff.

The Trial is designed to answer how the use of the Cytosponge™ would work in standard clinical practice in primary care, based on the successful completion of the hospital-based trial, BEST2. Hence a pragmatic clinical trial is required and the most appropriate control arm is ‘usual care’. As uptake is an important contributor to the primary outcome the intervention will be ‘invitation to the Cytosponge™-TFF3 test’.

Anonymised data will be collected from all patients in both arms at baseline (this step will be optional depending on local capacity) and one year post entry into the study. Patients will be informed about being entered into BEST3 data collection by letter.

Advice was sought from three NCRI groups on all aspects of the study design and logistics. Furthermore, input on the study design and trial documents was provided by a panel of PPI representatives based at Cambridge University Hospitals NHS Foundation Trust. This study design is set within the MRC framework for the design and evaluation of complex interventions [17]. Further advice has been sought prior to adding the individual randomisation procedure – this will maximise the statistical power of the study given the highly variable participation rate between practices.

Patient diagnosed with any new conditions as a result of the Trial will be treated in line of standard NHS treatment.

[2] PPIs and H2RAs

6 Study setting

We will recruit up to 150 general practices from across the UK. For IT reasons, only practices using Egton Medical Information Systems (EMIS) or SystmOne, the two most commonly systems, will be included in the study. Linked NHS trusts will be further recruited to provide confirmatory endoscopies for participants who receive on positive Cytosponge™ results and provide research endoscopies after the end of the follow-up period.

7 Eligibility criteria

7.1 Eligibility criteria for the BEST3 data collection

**Inclusion Criteria:**	**Exclusion criteria:**
• Male and female<br>• Aged 50 and over<br>• Records of at least six months of prescription for acid-suppressant medication in the last year*	• Recorded regular prescriptions of NSAIDs<br>• Recorded upper GI endoscopy in the preceding 5 years as identified from the practice database<br>• Recorded diagnosis of a current or previous oro-pharynx, oesophageal or gastro-oesophageal tumour<br>• Recorded diagnosis of Barrett's oEsophagus (BE)<br>• Unable to attend the GP surgery<br>• Deemed not fit enough by their GP, including lacking capacity

Eligible patients will be identified via GP database searches. GPs may exclude patients if they feel it would be inappropriate to include them in the Trial for example for lack of mental incapacity or long-term illness.

Patients who at the point of screening are found to, despite having had prescriptions for 6 months of acid suppressant medication, might not have been able to take 6 months' worth of medication in the last year, will still be able to receive the test if otherwise eligible. Endoscopy in the preceding 5 years has been included as a high-level exclusion criteria to act as a methodological tool to maximise BE detection of the study. If a patient confirms that they have received an Upper Gastrointestinal endoscopy in the preceding 5 years at the point of screening for the intervention, the patient will continue to be included and receive the Cytosponge™ (if eligible against the safety related criteria).

*Any patient identified by electronic searches meeting this criterion will be deemed to meet the criterion even if subsequent manual examinations of patient records show that they had less than 6 months' worth of acid-suppressant prescriptions in the year preceding recruitment, provided there is no evidence of a complete lack of

prescriptions or symptoms prior to enrolment. (Any patient with evidence of no prescriptions and no symptoms will be classified as a protocol violation and excluded.)

To achieve balance in across arms and the appropriate sample size, the study team may institute a 50:50 split for females: males in line with known BE prevalence, at the discretion of the Trial Statistician. This step may be taken if the proportion of females to males consistently exceeds 55:45 within the overall cohort.

7.2 Eligibility criteria for Cytosponge™-TFF3 Test

**Inclusion criteria:**	**Exclusion criteria:**
• Same as in 7.1	• Withdrawn from BEST3 study/BEST3 Data collection<br>• Meeting the guidelines for an urgent endoscopy referral according to NICE guidelines (dyspepsia together with significant acute GI bleed, un-investigated dyspepsia that fails to respond to PPI or H2RA and Helicobacter pylori testing, progressive dysphagia, progressive unintentional weight loss, persistent vomiting, iron deficiency anaemia, epigastric mass or suspicious barium meal, where the risk of gastric cancer or anxiety about cancer is heightened)<br>• Recorded diagnosis of an oro-pharynx, oesophageal or gastro-oesophageal tumour (T2 staging and above), or symptoms of dysphagia<br>• Difficulty in swallowing due to a known cerebrovascular accident or neurological disorder<br>• Recorded oesophageal varices, cirrhosis of the liver<br>• Inability to temporarily discontinue anti-thrombotic medication prior to procedure<br>• Having eaten and drank within the preceding 4 hours<br>• Received prior surgical intervention to the oesophagus<br>• Known pregnancy<br>• Unwilling to swallow beef gelatine capsule as part of dietary preferences<br>• Lacking capacity to provide informed consent

7.3 Eligibility criteria for research endoscopies (both arms excluding participants with positive result)

Inclusion criteria:	Exclusion criteria:
• Same as in 7.1	• Upper GI endoscopy during the study period<br>• Withdrawn from BEST3 study/BEST3 Data collection<br>• Lacking capacity to provide informed consent<br>• Known pregnancy<br>• Severe hypertension (e.g. systolic >200 diastolic >100)<br>• Myocardial infarction or any cardiac event within the previous six months.<br>• Cerebrovascular event or other neurological disorder where swallowing has been affected within the previous six months<br>• Any previous treatment such as Photodynamic therapy (PDT) or Radio Frequency Ablation (RFA) to the oesophagus<br>• Anticoagulation therapy/medication on day of procedure (warfarin, heparin or tinzaparin) according to local guidelines.<br>• Other medical condition: low platelets or blood abnormalities that may cause excessive bleeding post procedure<br>• Eaten or drank within the preceding 6 hours<br>• Preference for sedation and has not brought anyone or has anyone to accompany them at home. Follow local guidelines

8 Trial procedures

8.1 Recruitment

Participating GP sites will be recruited via the central trial team. This model is determined by the availability of treatment cost funding from NHS commissioners in each geographical location. The MHRA will be informed of primary care sites randomised to deliver the Cytosponge™ prior to the use of the device at each site.

Up to 150 practices will be recruited from multiple CRNs: North Thames, North East and North Cumbria, Eastern, Wessex, Thames Valley, North Thames, South West Peninsula, Yorkshire and East Midlands. Other regions may participate in the Trial as required in line with sample size requirements and treatment cost support. In the first instance, endoscopy units interested in participating in the Trial will be identified and practices linked to these units. Local CRNs will then identify and approach suitable practices amongst these. Lead GPs will be identified for each CRN to help with

recruitment. If the primary focus on endoscopy units and linking back to practices creates challenges for practice recruitment, the CRN will identify suitable practices not linked with any specific endoscopy units. New suitable endoscopy units will then be recruited based on these practices.

For cluster randomisation, practices will agree to participate in the BEST3 Trial prior to them being randomised into either of the two arms (intervention or usual care). In line with Sponsors' requirements, appropriate research agreements may be put in place depending on the level of involvement of practice staff. A statement of activities and schedule of events will be developed accordingly

8.2 Patient identification

Patients at participating GP sites will be selected by GP staff based on their inclusion and exclusion criteria above and following randomisation of the respective practice.

8.3 Consent procedures

In randomised trials, consent can occur at two levels [18]:

(1) Consent to the Trial occurring at the collective unit, here the practice, to take part/be randomised within the Trial
(2) Consent to receiving the intervention, i.e. individual-level consent

8.3.1 Consent at practice level (Opt in)

Firstly, consent will occur at practice level. GPs will provide consent that the practice will take part and participants contacted within the BEST3 Trial. For cluster randomisation, GPs will only be aware of the arm that they have been randomised to following agreement to participate in the Trial to avoid bias. GPs will furthermore consent to anonymised patient data being collated from their practice database and patient notes.

8.3.2 Introductory letter provided to patients about use of anonymous data (BEST3 Data collection)

All participants across both arms, for individual as well as cluster randomisation, will receive an information letter from their practice outlining that anonymous data collected in the course of their routine care will be included in the study. They can opt-out of having their anonymised data collected and analysed as part of BEST3. They will have 14 days to opt out of their data being collected, but they will be able to withdrawal at any time after these 14 days. All letters will be marked 'Return to Sender' to allow participants to be removed from the dataset as required.

8.3.3 Written consent for BEST3 Intervention study (Opt in written consent)

Written (individual-level) consent will be obtained before carrying out any procedures (including swallowing a Cytosponge™ or receiving an endoscopy). All participants receiving a Cytosponge™ –TFF3 test or endoscopy as part of the study will be individually-consented to have this procedure and for the associated clinical data to be collected. Participants will have the opportunity to discuss the nature and objectives

of the Trial, and possible risks associated with their participation with a member of the research team or practice staff.

Participants will receive written material that is REC-approved. Furthermore, at consent, participants will agree to their personal identifiable data (PID) being held by the central study team at the University of Cambridge and Queen Mary University of London (QMUL) for the purposes of the BEST3 Trial, including direct communications from the lead site. Consent will also be obtained for the use of anonymised clinical and genomic data and human tissues samples in future research, including by other organisations in the UK and overseas and the commercial sector. This will be for patients receiving trial-specific interventions such as Cytosponge™, saliva and biopsy collection.

The Principal Investigator (PI) will retain overall responsibility for the informed consent of participants at their site. They must ensure that any person delegated responsibility to participate in the Informed Consent process is duly authorised, trained and competent to participate according to the ethically-approved protocol, principles of Good Clinical Practice (GCP) and Declaration of Helsinki. The taking of consent can be delegated to research nurses (or in specific circumstances, practice nurse), who have undertaken NIHR Informed Consent Training or equivalent.

A person is assumed to have the mental capacity to make a decision unless it is shown to be absent. Mental capacity is considered to be lacking if, in a specific circumstance, a person is unable to make a decision for him or herself because of impairment or a disturbance in the functioning of their mind or brain.

- A capable person will:
  - understand the purpose and nature of the research
  - understand what the research involves, its benefits (or lack of benefits), risks and burdens
  - understand the alternatives to taking part
  - be able to retain the information long enough to make an effective decision
  - be capable of making this particular decision at the time it needs to be made (though their capacity may fluctuate, and they may be capable of making some decisions but not others depending on their complexity)

Where participants are capable of consenting for themselves but are particularly susceptible to coercion, it is important to explain how their interests will be protected.

7.2 Trial procedure summaries in chronological order

Study procedures for both arms are outlined below in Table 3a.

Table 3a: Study procedures for practices in both arms

Study procedure (chronological order)	Method	Person responsible and additional information
**Initial database search to determine number of eligible patients and practice randomisation stratum**	Automated GP database search	Practice staff
**Practice agrees / consents to participate in BEST3 Trial**	Written confirmation including confirmatory email for example HRA documentation	PI or PI representative
**Randomisation (Cluster randomisation practices only)**	Practices taking part in the cluster randomised design are randomised to either intervention or usual care.	KCL CPTU research team
**Identification of eligible patients to be included in study**	Automated GP database search based on coded data	Practice staff
**BEST3 Introductory letter sent to those eligible**	Automatic mail-out from database search after GP approval	Practice staff
**Patient randomisation (Individual randomisation practices only)**	Practices taking part in the individual randomised design will randomise patients to either intervention or usual care.	Practice staff
**Screening log**	Screening log of patients entered into the study will be kept at GP surgery	Practice staff
**GP training**	Measures to strengthen coding of endoscopies / BE diagnosis	Central team
**Baseline data collection (this step will be optional depending on local capacity)**	Batched, aggregated data collection for all patients who received an introductory letter and did not object; Automated GP database search (no manual checks)	Practice staff
**Data collection after end of follow-up**	Partly participant-level and part aggregate data collection from patient based on screening log<br><br>Automated GP database search + random manual checks where endoscopy referral has been coded + encrypted NHS number	Practice staff

	linkage with secondary care records (when possible)	
**Endoscopy invitation to ~500 patients who have not had an upper GI endoscopy during the study period**	- Random selection<br><br>- Invitation letter<br><br>- Consent to endoscopy<br><br>- Endoscopy carried out at local endoscopy centre<br><br>-Endoscopy findings retrieved from GP records	Practice staff for non-consented participants, research nurses for Cytosponge™ participants
**Health economic data**	Derived from GP health utilisation data as collected above	Practice staff

The following additional procedures only apply to practices in the intervention arm:

Table 3b: Study procedures for the BEST3 Intervention arm (Intervention Practices/Intervention patients)

**Study procedure (chronological order)**	**Method**	**Person responsible and additional information**
**Invitation letter to Cytosponge™ intervention + simplified leaflet**	For those who have not objected following Introductory letter (to be confirmed via screening log)	Practice staff. Reply slip include email/text options including consent to be contacted by research staff
**Follow-up phone call + scheduling of appointment**<br><br>**(Patients will receive a total of 1 reminder in the form of a letter, phone call or text message by way of reminder)**	Eligibility criteria for Cytosponge™	Research nurse or Practice staff
**Letter with further information and Intervention PIS/consent form**	Letter and PIS/consent	Research nurse

**Cytosponge™ appointment**	- Informed consent<br>- Demographic/clinical info collection including GERD Impact Scale<br>-Administer Cytosponge™<br>- Baseline questionnaire	- Consent by designated research nurse<br>- Demographic and clinical info collected straight onto electronic CRF by study nurse<br>- Sponge administration by designated nurse<br>- Baseline questionnaire to be filled in paper copies to be entered into the database by study nurse
**Patient questionnaire 7-14 days**	7-14 days questionnaire	Research nurse to coordinate
**Recording ADEs 7 days post intervention**	Telephone call at 7 days	Research nurse
**Cytosponge™ processing**	- Cytosponge™ sent to CUHTB (or similar lab) directly from GP surgery or roaming research nurse<br>- Result recorded by CUHTB (or pathologist) on trial application (trial app) within 4-6 weeks of procedure	Research nurse to ensure results reported within 6 weeks and GP informed and f/up decision made
**Patient follow up and Cytosponge™ result**	- GP and research nurse notified by automated email of results if TFF3+<br>- Advise on management and record (medication (type and duration), number of GP visits, symptom resolution)	- if TFF3+, GP to write to patient AND refer for endoscopy within 6-8 weeks Research nurse to organise and follow up<br>- GP letter with Cytosponge™ result<br>- GP to arrange further patient appointment as required<br>- Option for patient to phone study nurse for more info<br>- F/up data entered onto CRF
**Endoscopy for patients with positive result**	- Referred on basis of Cytosponge™ result if positive<br>- Record endoscopy findings (macroscopic with photo and biopsies)<br>- Collect saliva, including at home for convenience	-Research nurse to liaise re appointment and enter these data onto trial app.<br>- Research nurse to ensure all OGD and path findings completed
**Patient acceptability**	(i) Recruitment/uptake rate for Cytosponge™ and endoscopy<br>(ii) Patient acceptability measures to be complete:<br>- Pre- Cytosponge™ procedure:, STAI-6 (prior to formal consent)	Baseline: Research nurse to provide patients with paper questionnaires to be completed at the Cytosponge™ appointment and responsible for data entry to trial app<br>7-14 day follow-up: Questionnaires emailed automatically via trial app or paper copies sent by delegated GP staff

	- Post- Cytosponge™ procedure: perceived risk of OC visual analogue scale, Inventory to Assess Patient Satisfaction<br>(iii) 30 semi-structured patients interviews within 6-8 weeks of their trial consultation	in the post depending on patient preference<br>Qualitative researcher to carry out patient recruitment and interviews in year 2 of study
**Health care professional acceptability**	Semi-structured interviews with up to 20 GPs and 20 research nurses	Qualitative researcher to carry out patient recruitment and interviews in year 2 of study

8.4 Practice randomisation (Cluster)

Practices will be randomised via block randomisation.

To simplify randomisation and avoid any imbalances, stratification by practice size will not be taken into account during randomisation for the remaining cluster randomisation practices but instead later in the analysis. Randomisation will also be balanced by geographical area to ensure that Cytosponge™ clinics will have similar number of bookings in each area.

If the observed uptake is lower than expected (50%), the total number of participants enrolled at practices may be adjusted to increase the overall number of patients in an individual practice’s cohort. A minimum of 120 practices would still be required to achieve the appropriate power. The number of patients at a practice may also be reviewed if a large number of patients are deemed ineligible owing to their GP not agreeing to the discontinuation of anti-thrombotic medication.

8.4.1 BEST3 Introductory letter

All patients aged 50 and over who have received at least 6 months’ supply of an acid suppressant drug (either PPI or H2RA) in the last year will be identified by carrying out a database search on coded clinical information (Figure 5). These patients will be sent an introductory letter from their treating clinician. This letter will inform them that their practice is taking part in the Trial and that anonymised routinely-collected data will be retrieved and analysed by the BEST3 study team at both baseline and end of follow-up. This letter will additionally explain that they may receive an invitation to participate in later stages of the study.

As indicated in figure 5A and B, both cluster and individually randomised practices will receive the same introductory letter.

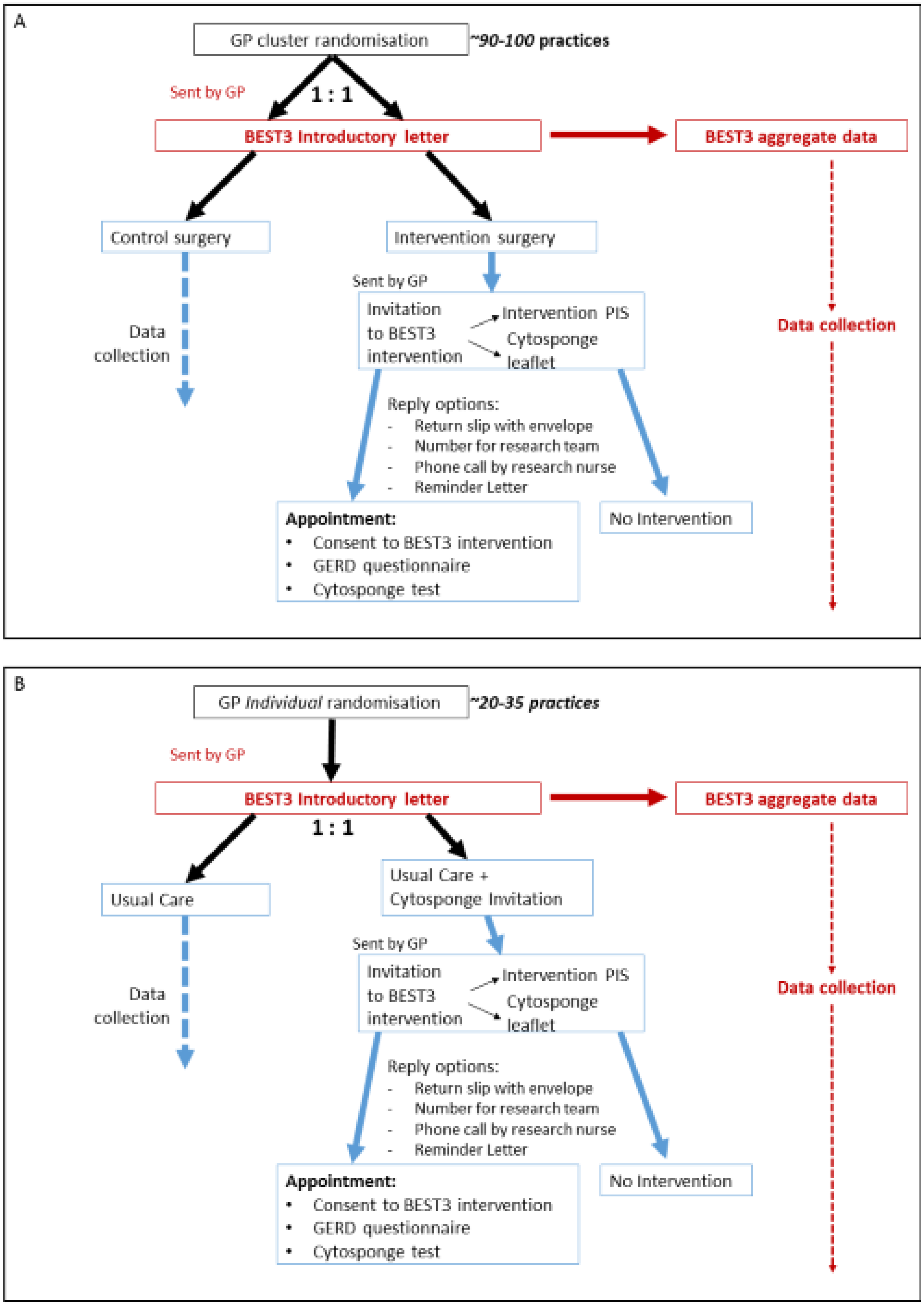

Figure 5: A) Patient recruitment diagram for cluster randomised practices and B) for Individually randomised practices

A screening log recording every patient sent an introductory letter will be kept in each practice. Any patient who was sent an introductory letter will be counted as recruited to the Trial, and the trial coordinating team at CPTU will be informed of the number of participants for each practice. The letter will explain that patients can opt out of their

anonymous data being included in the Study by calling their practice and asking not to take part within the 14 days after receiving the letter. This will be noted on the screening log. Any patient who opts out based on the letter will not receive an invitation for a Cytosponge™-TFF3 (intervention arm) or a research endoscopy (both arms). Patients whose letters are returned due to unknown address will also be removed.

8.4.2 Anonymised data collection for BEST3 Trial

Data management procedures are explained in more detail in Section 13. The dataset will be sent by secure transfer medium and loaded to the BCC IT database server at QMUL. Study entry will be considered as 14 days after the date the introductory letter is sent. At this point baseline demographic, upper GI symptoms and medication data will be extracted from the GP database for every patient entered into the study. This dataset will then be partly participant-level and partly aggregated into sex and 10-year age groups (Table 4 and standalone Data Table).[3] This step will be optional depending on local capacity and will apply to all trial sites

Table 4: Baseline and follow-up data to be collected

**Baseline data extract**		**Follow-up dataset**	
**Time point / period**	**Variable**	**Time point / period**	**Variable**
**Baseline**	Sex	**Baseline**	Sex – **Participant-level**
	Age		Age
	Obesity records	**Baseline and end of follow-up (where available)**	Obesity records
	Smoking status		Smoking status
	Alcohol consumption		Alcohol consumption
**Previous 9-15 months (depending on length of follow-up)**	PPI / H2RA prescriptions	**Previous 9-15 months (depending on length of follow-up)**	PPI / H2RA prescriptions
	Other prescription medication: Aspirin, antibiotics for H Pylori eradication		Other prescription medication: Aspirin, antibiotics for H Pylori eradication
	Heartburn and / or GERD related symptoms		Heartburn and / or GERD related symptoms
			Number of GP consultations at the practice
			Number of endoscopy or GI referrals - **Aggregated**
			Diagnosis of BE
			Diagnosis of EAC or pre-malignant conditions
			Diagnosis of benign oesophageal conditions
			Records on any upper GI specific procedures, e.g. endotherapies or oesophagectomies

[3] For the avoidance of doubt, the research team may add further fields as required to the standalone Data Table without the need for an ethical amendment.

			Anonymised endoscopy reports including pathology reports for BE and OC diagnosis; and benign conditions via a tick box (EoE, candida, inflammation, ulcer slough, squamous dysplasia, herpes, other)
			Type of referral: emergency via A&E, 2 week wait /urgent, routine and in or out patient (either form GP records or endoscopy report)
			Number of biopsies (from endoscopy reports)
			Anonymised letters from upper GI consultants

Follow-up data will be collected an average of 12 months (depending on follow-up period of the study site) after study entry for all patients in each practice, irrespective of study arm and whether they had a Cytosponge™-TFF3 test. Where new diagnosis of BE or EAC, an endoscopy has been coded in the medical records, data will be extracted from endoscopy and pathology reports and entered into participant-level and aggregate data CRFs (See Table 4 and Standalone table BE scores and cancer details).

Practices recruited in the latter stages of the Trial may adopt shorter follow-up periods to allow timely completion of study activities with a simulation tool used to ensure parity across the datasets (see Section 12.2.3 on variable follow-up periods).

8.5 Patient randomisation (Individual)

For practices participating in the individual randomisation procedure, the practice staff will be given instructions to run an automated procedure to randomise eligible patients to either usual care or intervention. There will be no formal upper limit to the number of patients randomised per practice although a limit may be agreed with the GP site so that the workload is manageable.

8.6 BEST3 Intervention: Cytosponge™-TFF3 test

8.6.1 Patient invitation to BEST3 Intervention

The trial liaison practice team member will update the local screening log to ensure that patients who objected to anonymous data being used in the Trial will not be contacted. All participants in the intervention arm will receive a second letter from their GP team inviting them to have the Cytosponge™-TFF3 test. This communication will include a standalone Cytosponge™ information leaflet.

There will be three possible ways for a patient to be in contact with a research nurse to express further interest in the Cytosponge™ test.

- The letter will inform participants that they might receive a telephone call from their practice within a specified period of time.
- Participants will be provided with a return slip to express interest in the Cytosponge™-TFF3 test and request a phone call from the research nurse.

- They will be provided with a phone number for the research nurse to contact him or her directly.

On return of a reply slip or telephone call from the participant, the participant will receive a telephone call to further assess eligibility. A short questionnaire will be used by the nurse over the phone to determine if a participant is eligible to take part in the BEST3 intervention. The participant will then be sent a Cytosponge™ patient information sheet and consent form and appointment confirmation. A Cytosponge™ appointment confirmed on the telephone or email confirmation would be further ways of scheduling the intervention visit.

At 2 weeks after the initial invitation but dependent on local circumstances, all participants who have not responded to the Cytosponge™ test will receive one reminder which may be in the form of:

- Second letter
- Phone call from a suitable member of practice staff and in line with local arrangements
- Text message using the practice's standard service with the following message: We recently wrote to you about receiving a Cytosponge™ test as part of the BEST3 Trial. Please call us if you are interested in taking part and receiving the test at the practice.

8.6.2 Cytosponge™ clinic procedure

Cytosponge™ clinics for participants from several practices will be held by the local research nurse at a pre-arranged time convenient for the surgery and with appropriate medical cover in place. Written consent will be taken at the beginning of the Cytosponge™ clinic appointment, as described above. They will be asked to refrain from eating and drinking for 4 hours. Patients will be made aware on their appointment letter/email that they can continue to take their daily prescribed medication.

The STAI-6 will be provided to the patient pre-consent. Once consent has been obtained, the nurse will complete a questionnaire on demographic and clinical information and the previously validated GERD Impact Scale (GIS) with the participants [19] using CRFs in the BEST3 Database.

Patients will then be asked to swallow the capsule. The Cytosponge™ is a Class 1 device consisting of a spherical 3.0 cm diameter reticulated polyester compressed and encapsulated in a capsule (size 00). The sponge is attached to a length of suture material which passes out through the capsule.

The capsule is swallowed with the use of water (approximately 200mls) and allowed to reach the stomach while remaining attached to the suture which is held onto by the patient or nurse (and which is affixed to a card preventing inadvertent swallowing of the suture). In the stomach the capsule is left for up to 5 minutes where it dissolves allowing the sponge to expand to its full size.

Patients will be provided with the option of having an anaesthetic throat spray Lidocaine (Tradename Xylocaine) 10mg per spray (maximum of 4 sprays), prior to retrieval to minimise discomfort. The Sponsor may provide supplies of Lidocaine spray to practices delivering the intervention.

It is then withdrawn using the suture, and as it does so collects cells from the lining of the oesophagus. The retrieved sphere will then be placed in preservative liquid and stored in line with a trial-specific SOP.

If a patient fails to swallow the capsule, they will be asked to try again. Patients will be able to try up to two times before they are classified as “Cytosponge™ swallowing failure”. Linked anonymised samples (with date of birth) will be sent directly from the practice to CUHTB to be processed and analysed for TFF3 and H&E according to a specific SOP. In the event of a Cytosponge™ detachment or an obvious bleed the research nurse will immediately inform the GP as the patient falls under their duty of care for medical assessment. In the very rare event of inhalation of the Cytosponge™, the device would be removed by an appropriately trained health care professional. A specific emergency SOP (incorporated with the Cytosponge™ Handbook) will be provided and following medical assessment, the GP's normal emergency procedures will be followed. The research nurse will telephone all participants to receive the Cytosponge™ the patient at 7 days post-procedure to assess safety and report ADEs.

8.6.3 Cytosponge™-TFF3 results

Patients will be informed about the results of their Cytosponge™-TFF3 by a standardised Cytosponge™ feedback letter from their GP within 4-6 weeks (maximum 6 weeks) of their Cytosponge™ test. Patients will be given the option to phone the study nurse for more information on how to manage their condition, or to make an appointment with their GP. In cases where the test is a low-confidence negative result, the sample fails in processing or is equivocal the patient may be invited for a repeat test at a suitable location depending on local capacity. Other benign conditions of the oesophagus will be reported with the TFF3 result. Some patients can opt to receive their result (if negative) by system-generated text message.

8.7 Endoscopies

8.7.1 Invitation for endoscopies - Intervention arm: Patients with positive result

Patients with a positive TFF3 test will receive an invitation for an upper gastrointestinal (upper GI) endoscopy (to take place within a maximum of 6-8 weeks from receiving their Cytosponge™ test result) at their local hospital-based endoscopy clinic to test for BE. The research team will arrange an endoscopy appointment for the patient. They may receive a phone call from the research nurse within a specified period of time to answer any queries and remind patients to attend their endoscopy appointment. Patients with a diagnosis of Dysplasia or above will only receive a standard clinical feedback letter (and not an additional research letter) due to the sensitivity of the findings.

The primary endpoint diagnosis of BE[4] on the routinely recorded endoscopy and pathology reports will be defined at three different levels of certainty:

- Diagnosis by the endoscopist or gastroenterologist following BSG guidelines
  - >3cm likely correct
  - <3cm more suspect unless biopsy with IM
- Confirmed by study pathologist or gastroenterologist
  - >C1 or >M3 +IM on biopsy
- IM on biopsy any length

A decision will be made on final endpoint as the data will be used for the primary outcome after initial results have been reviewed blindly. This will be clearly specified in the Statistical Analysis Plan.

Endoscopy departments in participating hospital trusts located in the catchment area of the participating practices will be encouraged to record both circumferential (C) and maximal (M) lengths and to take 4 biopsies every 2cm of suspected BE, but the blinded review may sometimes have incomplete information. As a secondary endpoint we will also use a scoring system using for BE according to severity (Table 5). This will be further developed after initial results have been reviewed blindly as part of a separate SOP, for example categories 2 and 3 could be subdivided according to whether IM is present.

A central review of endoscopic biopsies will be undertaken to provide the necessary reassurance around standardisation of BE pathology review.

Table 5: Proposed BE scoring system

Score	BE severity
**0**	Pathology report not available
**1**	Intestinal metaplasia (IM) on biopsy and endoscopic findings not seen in categories below
**2**	C1 or C0 up to M3 + IM
**3**	C2 or more, C0 M4 or more +IM
**4**	C3 or more
**5**	Low grade dysplasia (LGD)
**6**	High grade dysplasia (HGD) or T1a cancer

[4] Which also could be determined by a blinded adjudicating committee.

Endoscopic images may be requested/mandated (especially short ones) to try to exclude mis-diagnosed hiatus hernias and IM at normal appearing gastro-oesophageal junction.

Patients will then be asked to provide a saliva sample using the Oragene DNA kit for future genetic research. Instructions on how to collect the sample will be enclosed in the kit. Patients will be asked not to eat, drink, smoke or chew gum for 30 minutes before giving the saliva sample. Saliva samples will be stored for future DNA extraction. This could be collected at a hospital clinic or patient home depending on local circumstances.

8.7.2 Invitation for research endoscopies - Patients who do not require diagnostic endoscopy (all arms, excluding participants with a positive Cytosponge™ result)

Approximately 800 participants who have not had an endoscopy during the study[5] will be invited for a research endoscopy around 12 months after entry into the study (in any case, after end of follow-up). This will allow analysis of the following:

- Acceptability of endoscopy, i.e. comparisons between the acceptance of an invitation to endoscopy compared to a Cytosponge™ test.
- Perceptions around Cytosponge™ use and reliability, i.e. the extent to which patients feel they can rely on the Cytosponge™ test and do not need to go for an endoscopy.
- Number of patients with negative Cytosponge™ result, who are diagnosed with BE by endoscopy after the end of follow-up.

Depending on trial progress, only participants entered in the first half of the recruitment period will be invited to receive a research endoscopy. The total number of invitations and the proportion of each group may be adjusted due to time constraints or higher/lower than expected uptake. The latest expected number of invitations to a research endoscopy to be sent is approximately 500. Alternatively, a cap on the number of research endoscopies carried out might be set (approximately 40 in the Usual care arm).

Since the Trial will be assessing the acceptability of endoscopy by determining the uptake of invitations to endoscopy, the intervention here will be “invitation to endoscopy”. I

Participants to be invited will be selected at random from the screening log following a procedure implemented by CPTU. They will receive an invitation letter for a research endoscopy at their local hospital-based endoscopy clinic to test for BE. This letter will include an endoscopy-specific BEST3 Patient information sheet and a consent form. The letter will inform participants that they will receive a telephone call from their

[5] In particular, this will include:

- Participants in the Usual care arm
- And participants in the Intervention arm who received a negative Cytosponge™ test result

practice within a specified period of time to assess interest in an endoscopy and determine eligibility. In addition, they will be provided with a return slip to express interest in the endoscopy test. Participants who have declined or not responded to the original Cytosponge^{TM} intervention will also be invited in this step.

8.7.3 Endoscopy procedures: Patients with positive results

At the beginning of the research endoscopy appointment a research nurse or endoscopist will go through the details of the procedure and answer any questions. Patients will be asked to sign a study-specific consent form agreeing to undertake the endoscopy procedure and for any findings to be fed back into clinical care, see above. Where required by the hospital trust, an NHS consent form will be signed prior to this procedure as part of standard clinical practice. Standard trans-oral endoscopy following guidelines for diagnosis of BE will be carried out. During the procedure the endoscopist will note the diagnostic endoscopic landmarks for BE using a standard protocol and in line with the Seattle protocol:

- The findings will be entered by the hospital-based team in the endoscopy CRF as part of the BEST3 trial app. For all endoscopies where BE is found, biopsies will be collected (in all 4 quadrants) every 2cm according to surveillance guidelines (Seattle protocol). All biopsy samples will be processed and analysed by the local pathologist according to standard clinical practice including for benign conditions. Endoscopically-suspicious areas will also be targeted for biopsies.
- A further two biopsies from the GOJ will be collected (below the z-line) to determine whether intestinal metaplasia of the gastric cardia could account for a TFF3 positive test.
- Study participants will be informed about research endoscopy findings by letter from the research team / their GP.
- Endoscopic images may be requested/mandated (especially short ones) to try to exclude mis-diagnosed hiatus hernias and IM at normal appearing gastro-oesophageal junction

8.7.4 All other participants

The consenting procedure will follow 7.6.1 using a procedure-specific patient information sheet and consent form. Local trust arrangements will be followed with regards to clinical consent.

- For all endoscopies where BE is found, biopsies will be collected (in all 4 quadrants) every 2cm according to surveillance guidelines (Seattle protocol). All biopsy samples will be processed and analysed by the local pathologist according to standard clinical practice, including for benign conditions. Endoscopically-suspicious areas will also be targeted for biopsies.
- For patients with negative Cytosponge^{TM} result, a further two biopsies from the GOJ will be collected (below the z-line) for research purposes.

Local guidelines will be followed where participant has diabetes.

8.8 Acceptability measures- Intervention group only

8.8.1 Patient acceptability measures

The aims of the patient acceptability measures are:

- to measure if the Cytosponge™-TFF3 test is making patients more worried about cancer
- to ensure that the Cytosponge™-TFF3 test does not cause any short-term emotional harm
- to ensure that the Cytosponge™-TFF3 test does not cause any long-term physical harm

Baseline: Participants receiving the Cytosponge™ will be asked to complete a baseline questionnaire consisting of:

I. STAI-6, a short-form of the state scale of the Spielberger State-Trait Anxiety Inventory (STAI): This 6-item self-completed scale has been widely used to measure short-lived health experience [22]; To aid the appointment process, and to ensure that the state being measured accurately reflects views about the procedure, the questionnaire will be provided the patient pre-consent in the clinic waiting room area. If the patient does not go on to consent into the study, their questionnaire response will be disposed of securely.

II. Lifestyle and family history questionnaire

III. Perceived risk of oesophageal cancer, using 2 items which have been widely used for other cancer risk assessments to assess estimated percent risk of developing OAC and perceived risk compared with a person of the same age (relative risk) [21];

This set of questionnaires take up to 5 minutes to complete. The participant will be asked for their preferred method of completing follow-up questionnaires including by email with a web-link, or by mail with a paper copy.

7-14 day Follow-up: 7 – 14 days post-study consultation, all participants receiving the Cytosponge™ will be either sent an email or text message with a link to an online questionnaire (or mailed a questionnaire as preferred). This questionnaire will consist of:

I. the Inventory to Assess Patient Satisfaction, used following flexible screening sigmoidoscopy by Schoen et al , and having a 5 point ordinal scale with 18 items [24];

II. a visual analogue scale (VAS) in which 0 represents “Completely unacceptable,” 10 represents “Completely acceptable,” [23];

III. Perceived risk of oesophageal cancer [21];

IV. STAI-6 [22]

The set will take up to 10 minutes to complete. If the follow-up questionnaire has not been returned after 2 weeks, a further reminder will be sent. This might be followed with a reminder phone call after a further 2 weeks later. The Inventory to Assess Patient Satisfaction has been adapted for the Cytosponge™ test and will be validated using face validation with 6-12 patients who are either at high risk of BE, or have had the Cytosponge™ test.

8.9 12-month (Average) follow-up

Length of follow-up will vary between 9 and 15 months depending on the study sites (see Section 12.2.3 on variable follow-up periods).

The NHS number of recruited participants from each practice will be encrypted and sent to the central trial team. Additionally, the encrypted NHS number of all patients with a BE diagnosis made during the study period in participating (Secondary care) endoscopy sites (and possibly other local endoscopy units) will be sent to the central trial team. All sites (GP sites and endoscopy units) will use the same encryption. The central trial team will not be able to decrypt the information. The central trial team will look for encrypted NHS numbers in common between the two sources. When a match is found, the endoscopy unit concerned will be asked to ensure that full details of the BE diagnosis are transmitted to the patient's GP and the GP will be asked to ensure that the diagnosis is appropriately coded (as it is standard of care). This will enable the trial to obtain BE diagnosis data required to meet the primary objective. Additionally, local GP practices will conduct automatic and manual case note reviews (on at least some of the participating patients) to identify additional data required to meet secondary trial objectives including resource use.

8.10 Long-term follow-up

When suitable anonymisation models become available, long term cancer registration and mortality data will be obtained from NHS Digital and the NHS Health and Social Care Information Centre (HSCIC) or equivalent. A sophisticated encryption mechanism will be used to transfer patient details directly from practices to HSCIC. .

Individually-consented participants in the Trial may have their longer-term heath status followed up via data held by NHS Digital, NHS Health and Social Care Information Centre (HSCIC) or its successor, the Office of National Statistics, Public Health England and other national databases via a linkage completed by QMUL as holder of the identifiable data. This is in line with the requirements for safety monitoring, funding conditions and maximising the individual's contribution to research.

8.11 Intervention acceptability for patients and health care professionals

8.11.1 Patients

Some participants from intervention practices will be interviewed to increase understanding of patient views on the Cytosponge™ and its use in the primary care

setting. Up to 30 patients will be interviewed within 6-8 weeks of their trial consultation. The research team will screen the patients who have consented to be interviewed to purposively sample for age and sex. Patients will be invited for interview in their own home or a place of their choosing; they will provide written consent prior to interviews commencing.

Data will be audio-recorded and transcribed professionally; it will remain confidential and will not be shared with their GP. Data will be analysed using Thematic Analysis, supported by NVivo. We expect the following themes to be explored in the analysis: patient views of acceptability of Cytosponge™ use in primary care; patient understandings and perceptions of 'risk' in relation to their symptoms. As qualitative analysis is an inductive and iterative process, further themes will evolve throughout the analytical process.

8.11.2 Healthcare professionals - GPs

Semi-structured interviews, either in person or over the phone, with up to 20 GPs from intervention practices will be undertaken to identify and gain an understanding of the facilitators and constraints influencing use of the Cytosponge™ in primary care routine clinical practice. GPs will be recruited purposively to sample as widely as possible (region, gender, age, trainer status, rural/urban location). GPs can choose to be interviewed face to face or by telephone: those who are interviewed by telephone will be asked to provide verbal consent at the beginning of the interview and also complete a written consent form to be returned by post. Data collection, transcription and analysis will be undertaken in a similar way to the patient interviews.

8.11.3 Research nurses

All research nurses involved in delivering the intervention will be asked to complete a short on-line questionnaire at the beginning and end of their involvement in the study. This will focus on issues around their training, patient recruitment to their clinics, and delivering the intervention. We will also undertake semi-structured interviews with up to 20 research nurses from intervention practices, to identify and gain an understanding of the facilitators and constraints influencing use of the Cytosponge™ in primary care routine clinical practice. Research nurses will be recruited purposively to sample as widely as possible (region, gender, age, trainer status, rural / urban location), for a telephone interview. The methods of data collection, transcription and analysis will be identical to the GP interviews.

Semi-structured interviews with up to 20 research nurses will be undertaken from intervention practices, to identify and gain an understanding of the facilitators and constraints influencing use of the Cytosponge™ in primary care routine clinical practice. As with the GPs, research nurses will be recruited purposively to sample as widely as possible (region, gender, age, trainer status, rural / urban location), for a telephone interview. The methods of qualitative data collection and transcription will be identical to the GP interviews. We will analyse the qualitative data in a similar fashion to the GP data. In addition, we will undertake descriptive analyses of the

questionnaire data, and use both datasets in a mixed methods analysis to look for overarching themes

8.12 Withdrawal criteria

Participants (including recipients of the invitation letter) are free to withdraw at any time from the Trial without giving reasons and without prejudicing his/her further treatment and will be provided with a contact point where he/she may obtain further information about the Trial. Under GDPR, patients consented on new patient information will be made aware of limitations in their rights to withdraw their identifiable data at a later date. The study falls into 2 major components which will have an impact on the nature of withdrawal:

- Analysis of anonymised GP-held records for both arms
    - As outlined above, participants in both arms will receive an introductory letter. All participants in both arms will be able to object (within 14 days) to their anonymised data from their GP records being subsequently transferred to the study team at any time. Once the data have been transferred there will be no possibility of removing that data from analysis. The data of any participants objecting after the initial 14 day period (or before the baseline extraction occurs) will not be collected after this date the objection is received.
- Collection of samples and data from individually-consent participants
    - Participants who undergo study related procedures and have data/samples collected will be individually consented. They will be provided with information about how to withdraw from the study in relevant patient information sheets and the BEST3 website.
    - At consent, participants will be made aware that they can withdraw from the study at any time without any adverse impact on their treatment. They will be made aware that any samples that have been taken as part of the study will continue to be analysed and data retained in the project however no new samples or data will be collected (except part-aggregated data collected as part of the observation study)
    - Participants will be made aware that their GP or study team may withdraw them from the study at any point. They will receive a standard letter informing them of this step.
    - Participants who actively withdraw from the study will no longer be contacted (by phone, letter or email) by the study team (London/Cambridge) or the practice in relation to the Trial.

Throughout the duration of the Trial, participants in all arms will be able to visit the study website for full information including accessing up-to-date contact details and the process for withdrawing their consent for both parts of the study.

8.12.1 Types of withdrawal

1. ***Withdrawal of consent**:* For participants who actively withdraw, their data and samples will be retained with no new procedures undertaken. Participants will be made aware that if they withdraw following the Cytosponge™ test, they, or their GP, will still receive these results including for benign conditions. Any data already collected may continue to be analysed and reported.

2. ***Withdraw from future interventions:*** For participants who wish to opt of further procedures including repeat tests, endoscopy and further contact, any data already collected may continue to be analysed and reported.

3. ***Loss of capacity:*** For participants who lose the capacity to consent, their data and samples will continue to be retained in the study. No new data or samples will be taken. Their data will continue to be analysed.

The following scenarios should **not** result in participants being withdrawn from the BEST3 Trial:

4. ***Unable to swallow Cytosponge™:*** Their data may continue to be collected and analysed.

5. ***Inadequate Cytosponge™ test:*** Their data may continue to be collected and analysed.

6. ***Scheduled to have a clinical endoscopy during Trial:*** Participants in both arms who are referred by their GP to receive a clinically-indicated endoscopy will not be eligible for a research endoscopy after the end of follow-up. Unless specifically requested, their data may continue to be collected and analysed.

7. ***Not responding to invitation to or unwilling or unable to undertake research endoscopy after the end of follow-up:*** Participants will continue to be included in the study. Unless specifically requested, their data may continue to be analysed.

8. ***Subsequently deemed ineligible for the Cytosponge™:*** Based on further review following written consent, participants found to not be eligible will not be withdrawn. Unless specifically requested, their data may continue to be collected and analysed.

8.13 End of Trial

The end of the Trial will be when the last practice has extracted the last data from GP records i.e. an average of 12 months (9-15 months, depending on study site) after the last practice has extracted their baseline aggregate data.

The end of the active recruitment of the Trial will be defined as the date 30 days after the final participant has completed their research endoscopy (where relevant) in either arms and data been inputted into the BEST3 App or when the final practice has completed its end-of-follow-up endpoint data collection.

Where possible, there will be a period of up to ten years for anonymised follow-up data to be retrieved and reviewed from General Practice datasets and other health records including data held by NHS Digital, HSCIC, Office of National Statistics and Public Health England after study procedures are completed. Individual consent from Intervention arm participants will be sought for identifiable flagging of data for research outside this current protocol.

The Chief Investigator will inform the REC and MHRA of the end of the Trial within 90 days of its completion. A summary of the final research report will be sent to the REC and MHRA within 12 months of the end of the study.

9 Investigational medical device

9.1 Name and description of device

The Cytosponge™ is a single-use, non-sterile, 3cm diameter, polyester, medical grade sphere on a string, compressed within a gelatine capsule. The device is classified as Class 1 as defined in Annex IX of the Medical Devices Directive 93/42/EEC.

- The use of the device is “transient”, (Definition 1.1) the whole procedure taking less than 10 minutes.
- The use of the device is “invasive”, but not “surgically invasive” in that the entrance of the device is via a “body orifice” , namely mouth and throat (Definition 1.2)
- According to Rule 5 (Annex IX, Section 3, Clause 2.1) all invasive devices with respect to body orifices, other than surgically invasive devices and which are not intended for connection to an active medical device: are in Class I if they are intended for transient use.

Generic name: Barrett’s oesophagus Cytosponge™ test kit (**FPB-11-0022-SP)**

- Device is intended for single use
- Device will not be provided sterile (Annex I Section 8.4 & 8.5)
- Shelf life: 6 months

Manufacturer: Cambridge University Hospitals NHS Foundation Trust (CUH), Box 277, Addenbrookes Hospital, Cambridge Biomedical Campus, CB2 0QQ. Supplied by Europlaz Technologies Ltd, 1-9 The Maltings Industrial Estate, Southminster, Essex CM0 7EQ. This company has been successfully manufacturing the non-CE marked device for research study, BEST2 and other international studies.

Labelling: A unique identifier will be added by the manufacturer to aid device tracking and accountability. In line with regulatory requirements, labels will carry the following wording BEST 3 Clinical Trial — Chief Investigator Prof Rebecca Fitzgerald. This device is only to be used for the BEST3 Clinical Trial.

9.2 Legal status of the device

In early 2019, the CytospongeM achieved CE-marking for use in the UK. The Trial will continue to be conducted under a Notification of No Objection from the MHRA. Within the context of this study, the device will be only provided to approved primary care sites. The Barrett oESophagus Trial 2 Study (BEST2) demonstrated that the Cytosponge™-TFF3 test is safe and acceptable, and has accuracy comparable to other screening tests. A full analysis of accuracy, including sensitivity and specificity, and safety is presented in published results Document F (reference 20). A full rationale for the design specification can be found in Document B BEST3 FPB-12-0101-D_A1.

9.3 Technical design

Please see design note Document A BEST3 FPB-11-0022-SP for further details.

9.4 Device storage and supply

Cytosponge™ devices will be received from the manufacturer by KCL CPTU. Upon receipt, the study team will log the devices in on an individual device basis in the trial database. Devices will be logged to facilitate stock management and device tracking at GP practices and device tracking prior to dispatch.

The BEST3 App will have distinct functions to allow tracking of dispatch and use of devices, to facilitate safety reporting and to maximise stock supplies (i.e. by proactively monitoring expiry dates). Devices should be stored in locked room at room temperature.

The study team will be able to assess stock levels and expiry dates at the site level via the App and to conduct device accountability activities. Sites will be able to monitor their usage, request more supplies and record the use of the device on a per unit basis. This electronic system will assist in the event that device supplies are recalled by the manufacturer or at the request of other trial committees as part of safety management. Each device will have a unique identifier to further facilitate accountability, and safety-related measures. Expired devices will be placed in the clinical waste and logged using paper and electronic records.

9.5 Schedule of use

Participants will be provided with 2 opportunities to successfully swallow the device. All use will be logged on the BEST3 App. Participants will be asked to refrain from eating and drinking for 4 hours prior to the procedure.

9.6 Interaction with other therapies

Participants receiving anti-thrombotic medication will discontinue their medication under the medical guidance of their GP to minimise the risk of bleeding. Other restrictions are listed in Section 5. A PT-INR test may be conducted on patients on Warfarin prior to the Cytosponge™ procedure, in line with local arrangements.

10 Safety reporting and medical device vigilance

The section sets out the process for identifying, recording and reporting Adverse Events (AEs) and Serious Adverse Events (SAEs) and other safety events according to the requirements of the Good Clinical Practice and the Medical Device Directive in the BEST3 Trial. Further categories of Adverse Device Event and Serious Adverse Device will be used as it relates to the Cytosponge™ device (as an Investigational Medical Device). The approach used in the Trial is based on the outcomes of a risk assessment carried out by the Sponsor and all reporting will follow Cambridge University Hospitals NHS Foundation Trust's Standard Operating Procedure R&D/SOP011 Safety Reporting for Medical Device Trials and BEST3 Safety reporting SOP 006.[6] Further details for PIs can be found in the Trial's Investigator Brochure.

The study team will keep in close communication with the manufacturer including in the case of a serious adverse device event, device deficiencies and other quality control aspects. Main quality and safety issues will be reported via enquiries@europlaz.co.uk.

Definitions

- An adverse device event (ADE), as it relates to the use of the Cytosponge™ or Upper GI endoscopic procedure is defined as an untoward medical occurrence resulting from: insufficiencies or inadequacies in the instructions for use, deployment, implantation, installation, operation, or any malfunction, a use error or intentional misuse.

- A serious adverse device event (SADE), as it relates to the use of the Cytosponge™ or Upper GI endoscopic procedure, is defined as any adverse event that has resulted in any of the characteristics of an SADE, that (see Table 7):

    - led to a death

    - led to a serious deterioration in health

    - resulted in a life threatening illness or injury, (A Life-threatening refers to an event where the participant was at risk of death at the time of the event. It does not refer to an event which hypothetically might have caused death if it were more severe resulted in a permanent impairment of a body structure or a body function.)

    - required in-patient hospitalisation or prolongation of existing hospitalisation, (Any hospitalisation that was planned prior to randomisation will not meet SADE criteria. Any hospitalisation that is planned post randomisation, will meet the SADE criteria.)

[6] These SOPs will take precedence over this protocol section in the event of new version of the SOP becoming available. Following the SOP (where process is different to that specified in this protocol) will not constitute a protocol breach.

- resulted in medical or surgical intervention to prevent life threatening illness or injury or resulted in persistent or significant disability or incapacity
- led to foetal distress, foetal death or a congenital abnormality or birth defect

This includes device deficiencies that might have led to a serious adverse event if:

- suitable action had not been taken
- intervention had not been made
- circumstances had been less fortunate

Other non-device related SAEs will be reported in line with BEST3 SOP 006. A planned hospitalisation for pre-existing condition, or a procedure required by the protocol without a serious deterioration in health, is not considered to be a SAE.

Assessment of adverse events: Each SA(D)E must be assessed for causality, seriousness, severity and expectedness by the site PI and CI, including association with the Investigational Medical Device.

Assessment of relatedness: An adverse event should be categorised as unrelated or possibly related. Where an adverse event is deemed to be possibly related this will indicate that the nature of the event, the underlying medical conditions, concomitant medication or temporal relationship make it possible that the AE has a causal relationship to the research procedure.

Assessment of severity: The assessment of severity will be recorded on the CRF on the BEST3 App according to the following categories:

- **Mild**: an event that is easily tolerated by the participant causing minimal discomfort and not interfering with every day activities.
- **Moderate**: an event that is sufficiently discomforting to interfere with normal every day activities.
- **Severe**: an event that prevents normal everyday activities.

Assessment of expectedness: The investigator must make an assessment of the expectedness of the ADE based on knowledge of the effect and any relevant product information. Where the effect is not expected, the SADE will be deemed to be an unanticipated serious adverse event (USADE). In the Trial, a USADE, as it relates to the use of the Cytosponge™ or Upper GI endoscopic procedure, is defined as a SADE that, by its nature, incidence, severity of outcome, has not been identified in the current version of the protocol or risk assessment. A list of anticipated SADEs is set out below.

Table 7: Anticipated SADEs that are not required to be reported to the REC

Cytosponge-related	Endoscopy-related
Cytosponge ™ detached from the string while in the patient's oesophagus/stomach	Bleeding from biopsy site
Inability or difficulty to remove the Cytosponge ™	Perforation or tear of the oesophagus
Laceration at the back of the throat	Sedation complications e.g. hypoxia, allergic reaction
Obvious bleeding from the oesophagus	Damage to teeth
Perforation or tear of the oesophagus	

10.1 Scope

AEs and SAEs reported up to 7 days after the administration of the Cytosponge™ and 7 days post any endoscopy (in a study participant during the length of the Trial) will be recorded and reviewed. All participants receiving the Cytosponge™ will receive a telephone call at 7 days to assess any ADEs.

All SAEs occurring from the time of Cytosponge™ administration (up to 7 days post) and research endoscopy (up to 7 days post) must be recorded on the BEST3 App and reported to KCL CPTU within 24 hours of the PI becoming aware.

10.2 Mode of reporting

SAEs and AEs will be reported following the Sponsors' procedures (See R&D/SOP11) using specific CRFs and via the BEST3 App managed by KCL CPTU.

Site PIs (with delegation to research nurses registered on the site delegation log) must report SADEs within 24 hours of becoming aware of the incident to KCL CPTU as Sponsor delegate. KCL CPTU will alert the Sponsor as soon as they become aware of the event and the data has been QC'ed, in coordination with the Chief Investigator. KCL CPTU, having checked original report will alert the CI (and will transmit all views about seriousness and unexpectedness etc if there is disagreement). KCL CPTU will send notification to the Sponsor (research@addenbrookes.nhs.uk) cc'ing the CI (no PID should be transmitted to KCL CPTU or the Sponsor).

The initial report must be made by completing an AE/SAE eCRF accessible via the BEST3 App and a paper CRF completed with PI signature sent to best3trial@qmul.ac.uk (initials and day of birth redacted). If for any reason the web application cannot be accessed, a paper AE/SAE CRF should be completed and emailed to the study team. Sites should ensure that any patient identifiable information is not contained in any paper CRFs or documents when transferring to KCL CPTU. Any such information should be redacted and information not visible.

Reporting SAEs to KCL CPTU

Web app: https://www.cptu-edc.org
Email: BEST3trial@qmul.ac.uk

Any change of condition or other follow-up information should be sent by sites to KCL CPTU, and then onto the Sponsor, as soon as it is available or at least within 24 hours of the information becoming available. Events will be followed up until the event has resolved or a final outcome has been reached. Reports of related and unexpected SADEs will be submitted to the REC within 15 days of KCL CPTU becoming aware of the event. All SADES will be reported immediately to MHRA following advice from the Sponsors.

10.3 Recording of SA(D)Es

The trial information will be recorded in the participant's notes (both GP record and BEST3 App record). Each patient will be provided with an alert card and contact details indicating clearly whom to contact in the event of an ADE. Only ADEs (including SADE) occurring within 7 days of a research procedure will be recorded and investigated. For each SADE, the following information will be collected:

- full details in medical terms and case description
- event duration (start and end dates, if applicable)
- action taken
- outcome
- seriousness criteria
- causality (i.e. relatedness to study procedures), in the opinion of the investigator
- whether the event would be considered anticipated or unanticipated.

10.4 Electronic management of SA(D)Es

PIs, delegated to site research nurses, at GP sites will record SADEs directly onto the BEST3 App within 24 hours of becoming aware of the event/effect. This will generate an automated email to the KCL CPTU, the CI and the Sponsor. Details provided within the SADE CRF will include BEST3 ID, type of SADE, severity, outcome and remedial actions taken.

KCL CPTU will then assess the information received and immediately notify the Chief Investigator for a review of the seriousness, relatedness and expectedness of the event. An update about outcomes and remedial actions taken will be requested from the site research nurse/Principal Investigator and the SADE record reviewed by the Project Manager as complete (fully investigated, remedial actions taken and REC informed where relevant).

A report will be generated directly from the BEST3 App for KCL CPTU to forward to the Sponsor as soon as possible after data cleaning/QC. In the event of a difference in opinion between the PI and the CI, all views will be recorded and reported to the Sponsor. Specified users will be able to view all SA(D)Es including type of SA(D)E, severity, outcome and follow up actions in a single location on the BEST3 App. This will be in the form of a real-time project monitoring report.

Reporting to participating sites: KCL CPTU will report a summary of SADEs to participating sites including related safety information and Urgent Safety Measures, on a quarterly basis.

Reporting to Cytosponge™ manufacturer: The Chief Investigator, University of Cambridge Study team will report any safety concerns directly to the manufacturer.

Reporting to REC: All related and unanticipated SAEs will be reported to the REC by the Chief Investigator in conjunction with the Sponsor within 15 days of KCL CPTU and CI being made aware. SADEs (USADEs) will be reported by the Sponsor to the REC within 15 days of KCL CPTU and CI being made aware. This will follow the current HRA guidance: http://www.hra.nhs.uk/resources/during-and-after-your-study/progress-and-safety-reporting/ Safety aspects will be addressed in the Annual Progress Report to the REC by the Chief Investigator.

Reporting to the MHRA: All SADEs, involving the Cytosponge™ (falling within the scope of the Medical Devices Directive) will be reported immediately to the MHRA (devices) by the Sponsors in line with R&D/SOP011 Safety Reporting for Medical Device Trials

Record keeping: All reports of SADEs and USADES must be kept in the local site file (including AEs), Trial Master File and Sponsors' master files, as well the individual's GP-held health care record.

10.5 Device deficiencies

In light of the scale of the project, the DMC will be convened to independently review safety concerns, including trends, identification of affected device batches and remedial actions including implementation of Urgent Safety Measures. If any USM are taken the Sponsor shall immediately and in any event no later than 3 days from the date the measures are taken, give written notice to the MHRA and the relevant REC of the measures taken and the circumstances giving rise to those measures. Anonymised record-level data may be shared with the manufacturer and their associates for safety and product development purposes.

For the purposes of this Trial, device deficiency is defined as: inadequacy of a medical device with respect to its identity, quality, durability, safety or performance. Device deficiencies include malfunctions, use errors and inadequate labelling. KCL CPTU will be responsible for monitoring and reporting of device deficiencies to the CI and Sponsor, including related Urgent Safety Measures to sites. All device deficiencies will be recorded in the BEST3 App and recipients of related batches, informed in real time.

10.6 Responsibilities

This section outlines the responsibilities for safety reporting and review in the BEST3 Trial. Responsibilities between University of Cambridge/Cambridge University Hospitals NHS Foundation Trust (CUH lead sponsor for safety reporting) Queen Mary University of London and King’s College London have been defined in their collaborative agreement dated February 2019.

Stakeholder	Reporting	Review
**Principal Investigator (GP)**	1. Identifying AEs when participants attend for treatment / follow-up including post 7 days (delegated to team appearing on delegation log).<br>**2.** Using medical judgement in assigning seriousness, causality and expectedness.<br>**3.** Ensuring that all SAEs are recorded in the BEST3 App and reported to KCL CPTU within 24 hours of becoming aware of the event and provide further follow-up information as soon as available.<br>**4.** Ensuring that SAEs are chased with KCL CPTU if a record of receipt is not received within 2 working days of initial reporting.	N/A
**Chief Investigator**	1. Ensuring that AEs are recorded and reported to the Sponsor in line with the requirements of the protocol.<br>2. Using medical judgement in assigning seriousness, causality and expectedness of SAEs where it has not been possible to obtain local medical assessment.<br>**3.** Using medical judgement in assigning expectedness.	**1.** Clinical oversight of the safety of patients participating in the Trial, including an on-going review of the risk / benefit.<br>**2.** Review of specific SAEs in accordance Trial Monitoring Plan.
**Barts CTU up to March 2019, KCL post this date**	1. Development and maintenance of the reporting system.<br>2. Central data collection and verification of AEs and SAEs and according to the Trial protocol onto a database.<br>3. Reporting of SAEs to sponsor within as soon as possible after QC.<br>4. Notifying investigators of related and unexpected SAEs that occur within the Trial.<br>**5.** Following up on Urgent Safety Measures.	**1.** Providing safety information to the CI for their on-going assessment of risk/benefit.<br>**2.** Reporting general safety information to DMC.

**Sponsor**	**1.** Expedited reporting of unanticipated and related SAEs to the REC within 15 days.<br>**2.** Reporting of SAEs to MHRA in line with applicable processes.	N/A

11 Storage and analysis of samples

The study will collect several types of samples from participants and all will be handled in line with the Human Tissue Act 2004. Material transfer agreements will be put in place as required by the Sponsor and relevant consumables provided by the relevant stakeholder.

Table 6: Summary of BEST3 samples

Sample collected	Sample	Kit provided by (Cambridge or KCL CTU)	Storage conditions	Transport of samples (from / to)	Intended analysis
**Cytosponge™ samples**	Cytosponge™ device	KCL CPTU	Follow SOP for storage/ transport once sample is taken. Refrigerate at 4° degrees where possible	GP to CUHTB	TFF3 Test and benign conditions reporting
**Saliva samples**	Oragene. DNA Kit - room temperature	KCL CPTU	Room temperature and -20 degrees celsius where possible	Hospital trust to Cambridge	Rendered acellular DNA Sequencing
**Endoscopic biopsies - TFF3 positive (BE)**	Clinically-indicated sampling where BE or benign conditions suspected	N/A	In line with Trust procedures	N/A – local testing only	Histology as per usual practice
	Research sample: FFPE block with 2 biopsies from the Gastric Cardia 2 cm below the GOJ	N/A	Formalin – then processed to a paraffin block	In future research from NHS Trust to Cambridge where requested	For future research
**Endoscopic biopsies – 250 invites to randomly-selected negative Cytosponge™ patients**	Clinically-indicated sampling where BE or benign conditions suspected	N/A	In line with Trust procedures	N/A	Histology as per usual practice
	Research sample: FFPE block with 2 biopsies from the Gastric Cardia 2 cm below the GOJ		Formalin – then processed to a paraffin block	In future research from NHS Trust to Cambridge where requested	For future research
**Endoscopic biopsies – 250 invites to randomly-selected control patients**	Clinically-indicated sampling where BE or benign conditions suspected	N/A	In line with Trust procedures	N/A<br>In future research from NHS Trust to Cambridge where requested	Histology as per usual practice

All samples to be collected are summarised in Table 6.

- Cytosponge™ samples (intervention arm only) will be sent directly from GP sites to Cambridge University Hospitals NHS Foundation Trust Research Tissue Bank (or similar laboratories) to be processed into Formalin Fixed

Paraffin Embedded (FFPE) blocks for TFF3 testing, and H&E analysis following procedures outlined in the BEST3 Processing SOP 013. Samples will be transferred in pre-supplied media and sample containers with preservative. Courier will be arranged by KCL CTU / Cambridge. Analysis by CUHTB will take place according to a trial-specific SOP. Residual unprocessed Cytosponge material will be disposed of after analysis and FFPE material returned to the Hutchison/MRC Research Centre at the University of Cambridge.[7] Any material requiring disposal will be disposed of by the partner laboratory in line with the HTA procedures.

- Saliva samples (intervention arm only for participants with positive test result) will be sent directly from hospital sites or patient homes to the Hutchison/MRC Research Centre at the University of Cambridge (or similar laboratories) or similar laboratories as required via recorded Royal Mail delivery / pre-paid envelope.
- Endoscopy research samples (both arms) will be processed locally in NHS pathology departments. Additional biopsies may be taken at the discretion of the endoscopist and in line with clinical practice should abnormalities in the oesophagus be observed at the time of endoscopy. FFPE blocks may be transferred from participating NHS hospital trusts to the Hutchison/MRC Research Centre (or similar laboratories) in line with consent for future research.

Sample tracking: All Relevant Material that is to be disposed of will be logged appropriately on a tracking system and disposed of following the respective organisations' SOPs relating to HTA compliance and in line with the Human Tissue Act 2004. Residual Cytosponge™ and biopsy samples may be stored long-term in an HTA licenced facility at the University of Cambridge and used in future unspecified research (including whole genome sequencing) by the University and other organisations (including by overseas organisations and in the commercial sector) in line with patient consent and the appropriate regulatory requirements. New ethical approval will be secured as required.

It is the responsibility of the site to ensure that samples are appropriately labelled in accordance with the trial procedures to comply with the 1998 Data Protection Act. Biological samples collected from participants as part of this Trial will be transported, stored, accessed and processed in accordance with national legislation relating to the use and storage of human tissue for research purposes and such activities shall at least meet the requirements as set out in the 2004 Human Tissue Act. Custodianship for samples will transfer to the University of Cambridge and Cambridge University Hospitals NHS Foundation Trust. All agreed procedures, including standard reporting and manifest forms that will accompany samples, will be set out with the BEST3 Cytosponge™ Handbook. All transfers of human tissue samples will be covered under appropriate material transfer agreements between organisations, where deemed necessary by the Sponsor.

All use of human tissue samples (including analysis, transfer and disposal) will be conducted in line with organisational SOPs that comply with HTA and other

[7] Participants will be made aware that the University of Cambridge may commission other third party laboratories for analysis and long term storage of all trial samples as required.

regulatory frameworks. Analysis may be conducted in a variety of laboratories in the UK and overseas as the project demands, as it relates to the BEST3 Trial, including for cost savings as required. All receipt of samples, irrespective of the organisation conducting it, will be logged in Trial databases accessed by all organisations including originating site, lead site and partner laboratories.

As part of this protocol, we will undertake a range of genetic and biochemical analysis to assess known and yet-to-be identified risk factors in disease progression in BE and related conditions, including in future research projects by the University of Cambridge and its partners (UK, overseas and commercial sector). Analysis will include but not be limited to targeted, exome level and whole genome sequencing. With patient consent, genomic and linked clinical data may be deposited in a wide range of international repositories for access by international researchers. In light of the current knowledge base, no participants will receive any feedback related to the genetic analysis of their samples.

Patients will consent to the future use of their samples in related health research conducted by the University of Cambridge, and by its partners in the UK and overseas including the commercial sector. Samples at the end of the study will be stored in a HTA-licence facility at the University of Cambridge on completion of the ethically-approved project. Future research may include currently undefined genetic analysis including whole genome sequencing of which results may be deposited in a range of international repositories for access to researchers worldwide.

11.1 Laboratories

11.1.1 Central laboratories

Cambridge University Hospitals NHS Foundation Trust Research Tissue Bank (CUHTB) designated as the central laboratory responsible for the processing, analysis and evaluation of Cytosponge™ samples. Hutchison/MRC Research Centre, University of Cambridge (or commissioned laboratories) will act as the central laboratories designated to store, process and analyse saliva samples and FFPE tissue blocks.

11.1.2 Local laboratories

NHS Pathology departments, with relevant accreditation and meeting clinical standards, linked to local endoscopy units at hospital trusts will be responsible for the processing and evaluation of endoscopy biopsy samples.

12 Statistics and data analysis

12.1 Sample size and power calculations

The sample size calculation is based on the following assumptions:

i. BE prevalence in individuals eligible for the study is 4%
ii. 10% of patients in the usual care arm will be referred to endoscopy for clinical reasons (after excluding urgent referral)
iii. The prevalence of BE in patients referred to endoscopy in the usual care arm is 6%,
iv. Uptake of the Cytosponge™ test is currently expected to be 50%
v. Cytosponge™ -TFF3 sensitivity is 85%
vi. Endoscopy sensitivity is 100%

Since only 50% of patients in the Cytosponge™ arm are predicted to have the Cytosponge™ test and patients who do not take up the offer of the test will have the same management as if they were in the usual care arm we only expect 2.0% of patients in the intervention arm to be diagnosed with BE:

Percentage diagnosed with Cytosponge test

Percentage diagnosed without Cytosponge test

$$\frac{0.85*4\%}{2} + \frac{0.6\%}{2} = 2\%$$

Furthermore, we expect 0.6% of patients in the usual care arm to be diagnosed with BE. For a 90% power (not allowing for clustering) comparing 0.6% with 2.0%, we would need 3028 individuals (1514 in each arm).

To account for the fact that individuals within a cluster, here a general practice, might be more similar to each other than to individuals in other clusters, the number of participants required for an individually randomised trial has to be increased. The sample size calculated above was therefore multiplied by the variance inflation factor (VIF). We estimated the VIF as follows:

$$VIF \;=\; 1 + \{(cv\text{^}2 + 1)m - 1\}rho$$

Where cv is the coefficient of variation of the numbers of patients per practice, m is the mean number per practice and rho is the intra-class correlation (of the proportion of patients with BE), which is set to 0.025.

We allowed for variation in practice size and adjusted the sample size calculations for three groups of practices recruiting either 50-60, 61-74 and 75-100 patients. Allowing for intra-cluster correlation of 0.025, a coefficient of variation of 0.2, and mean cluster sizes of 55, 68 and 88 respectively we would need a total of 8988 patients. If we are recruiting three groups of 40 practices with 50-60, 61-74, or 75-100 patients each, then 120 practices will result in approximately 90% power. Assuming 50% uptake, this would result in 2247 patients having the Cytosponge™ test.

12.2 Timescale, potential challenges and milestones

Proposed timelines can be found in a specific project plan. The full study will run for approximately 3 years.

The main identified potential challenge would stem from an underestimation of the number of eligible patients and proportion of eligible patients who are willing to participate. There is a milestone at 6 months to run a formal review of the proportions of eligible individuals per surgery (% of population covered) and of participating individuals (% of eligible population), and of the Cytosponge™ uptake.

12.2.1 Milestone review 1: 6 months (January 2018)

As part of the scheduled milestone review at 6 months after opening the first GP site, the proportion of eligible individuals per surgery (% of population covered) and of participating individuals (% of eligible population), and of the Cytosponge™ uptake was evaluated. Uptake of the Cytosponge™ test was expected as outlined in section 12.1 to be 50%. Data to date has suggested uptake is actually 20% of those invited (with a further 7% awaiting an appointment). Given the substantial impact to the power of the trial, it was proposed that an additional individual randomisation design be added to the trial.

In a cluster randomised trial, individuals within the same cluster do not act independently, so it is necessary to randomise more individuals. We can calculate how many people one needs to include in a cluster randomised trial to provide the same amount of information as one person in an individually randomised trial. That number is called the variance inflation factor (VIF). With a VIF of 3.0, one would need 9000 patients in a cluster randomised trial to have the same power as 3000 in an individually randomised trial.

As we increase the number of invitees per practice, the “variance inflation factor” (VIF) increases, too. In simple terms, there are diminishing returns. For practical reasons, practices that have already commenced being set up, trained etc. on the cluster randomisation design, will be allowed to continue to randomise in a cluster fashion. Therefore, we don’t know exactly how many participants will be recruited within clusters but we envisage recruiting about 11,800 from ~100 cluster-randomised practices (equivalent to approximately 2920 individually randomised patients).

Originally, the trial was envisaged as recruiting patients presenting to their GPs with (incident) symptoms of reflux. For this reason, we: (1) felt that GP-practice-level randomisation was essential; and (2) assumed that with the personal endorsement of the GP, acceptance of the offer of a Cytosponge™ test would be very high (50%).

For practical reasons (related to trial delivery), BEST3 has been randomising “prevalent” patients i.e. practices identify patients who have drug prescription records indicating reflux and invite them (or not depending on randomisation) all at once. Identification using prescribing history rather than at the presenting appointment has resulted in a much lower uptake than anticipated (approximately 27%).

To obtain sufficient power without greatly expanding the number of participants and practices required, we plan to allow sites to complete individual-level randomisation. Practices already setup or trained to take part in the cluster-randomised arm will be

permitted to continue with this randomisation method. Newly engaged practices will randomise using individual randomisation methods.

At the time of Milestone 1 review (Jan 2018), the original sample size calculation in 12.1 was reviewed and updated:

The following parameters have not changed:

- BE prevalence (overall): 4.0%
- Endoscopy in usual care arm (over 12 months): 10%
- BE prevalence in patients getting routine endoscopy: 6%
- Cytosponge™ TFF3 sensitivity: 85%
- Endoscopy sensitivity: 100%
- Intra-class correlation: 0.025

The following parameter has been updated:

- Uptake of Cytosponge™: 27% (was 50%)

Additionally, the formula for estimating the percentage of those invited to a Cytosponge™ test found to have BE over the course of a year (see Section 12.1) has been updated to allow for those with a false-negative Cytosponge test to have the same chance of having BE diagnosed via routine endoscopy as in the control arm (10%). (Previously we assumed that no one with a false negative Cytosponge™ would have a subsequent endoscopy). This adjustment in the formula and the updated figure for the uptake of the Cytosponge™ test allowed us to estimate that about 1.4% patients in the intervention arm will be diagnosed with BE in a year's time.

Our estimate of the proportion of patients in the usual care arm diagnosed with BE does not change.

Requiring 90% power and a significance level of 0.05% gives a sample size of 6764 individually randomised patients.

For analysis, we will stratify practices according to the cluster size: 1 (i.e., individual-level), 51-65, 66-90, 91-125, 126-175,176-225. Taking into account the coefficient of variation within stratum, calculated as the standard deviation divided by the average size, we estimate the variance inflation factor in each to be: 1.00, 2.44, 2.96, 3.59, 4.77, and 5.91, respectively, for the practices that have confirmed their participation and for which we have data on their number of recruited patients to this date. Based on confirmed and projected numbers of participants in cluster-randomised practices, we anticipate 11,816 patients from 100 practices contributing the equivalent of 2924 individually randomised patients. That would leave 3840 to be recruited from practices employing individual randomisation and a total sample size of 15,656 participants overall.

The table below shows a range of possible values for our sample size depending on how we adjust the estimate of our parameters.

	BE prevalence	Cytosponge™ TFF3 sensitivity	Endoscopy in usual care arm (over 12 months)	BE prevalence in patients getting routine endoscopy	Uptake of Cytosponge™	Intra-class correlation coefficient	Sample size if only individual randomisation	Equivalent sample size accounted for by cluster randomisation (11,816 patients in 100	*Remaining sample size for current individual randomisation*
Our assumptions	4.0%	85%	10%	6%	27%	0.025	6764	2924	***3840***
Varying assumptions	***3.9%***	85%	10%	6%	27%	0.025	7098	2924	***4174***
	4.1%	85%	10%	6%	27%	0.025	6456	2924	***3532***
	4.0%	***84%***	10%	6%	27%	0.025	6894	2924	***3970***
	4.0%	***86%***	10%	6%	27%	0.025	6638	2924	***3714***
	4.0%	85%	***9%***	6%	27%	0.025	5658	2924	***2734***
	4.0%	85%	***11%***	6%	27%	0.025	8176	2924	***5252***
	4.0%	85%	10%	***5%***	27%	0.025	5048	2924	***2124***
	4.0%	85%	10%	***7%***	27%	0.025	9344	2924	***6420***
	4.0%	85%	10%	6%	***26%***	0.025	7190	2924	***4266***
	4.0%	85%	10%	6%	***28%***	0.025	6380	2924	***3456***
	4.0%	85%	10%	6%	27%	***0.024***	6764	3012	***3752***
	4.0%	85%	10%	6%	27%	***0.026***	6764	2840	***3924***

12.2.2 Update on uptake (March 2019)

In the updated sample size calculations of Milestone 1, uptake of the Cytosponge™ invitation was expected to be 27%. However, in the current data available, uptake seems to be closer to 23.7%, which would be equivalent to 83% power according to the same assumptions as in the section above.

A power of 80% will be guaranteed by the study as long as uptake does not fall under 23.0%. In any case, small tweaks to our initial assumptions (e.g. prevalence of BE in eligible population of 5% instead of 4%) should be discussed if the power of the study happens to fall under this threshold.

The following table shows how the power of the study varies by keeping the same sample size and changing some of the initial assumptions.

**Uptake**	**Prevalence of BE in eligible population**	**Prevalence of BE in those referred to endoscopy**	**Sample size (with 90% power)**	**Power with n = 6768**
27.0%	0.04	0.06	6768	90%
23.7%	0.04	0.06	8460	83%
20.0%	0.04	0.06	11018	72%
23.7%	0.05	0.06	5484	95%
23.7%	0.04	0.05	10126	75%
23.7%	0.05	0.05	6370	92%
23.0%	0.04	0.06	8886	81%
22.0%	0.04	0.06	9350	79%
21.0%	0.04	0.06	10126	75%

12.2.3 Variable follow-up periods

Because of delays in the implementation of the change of study design (from cluster randomised to cluster randomised + patient-level randomised), it was decided that some participants could have a follow-up longer than 12 months to compensate for participants who will have a follow-up shorter than 12 months due to the study time constraints.

Different practices will have different follow-ups as long as the weighted average follow-up for the study will be greater than 12 months, as shown by the following formula:

$$\text{avg}_{\text{FU}} = \frac{FU_{CR}Neq_{CR} + FU_{PR1}N_{PR1} + FU_{PR2}N_{PR2}}{Neq_{CR} + N_{PR1} + N_{PR2}} \geq 12$$

equivalent size for cluster randomised group (under Neq_{CR})

size of patient-level randomised group (confirmed + projected) (under N_{PR1} and N_{PR2})

According to our current projections, practices will either be on a 9, 12 or 15-month follow-up.

Patients who are lost to follow-up owing to IG restrictions in place locally effectively contribute 0 months of follow-up.

12.3 Statistical analysis plan

A separate statistical analysis plan will be written with full details of planned statistical analyses and signed off before examining the primary outcome in the main database. In brief, we are planning the following analysis. A summary of all secondary endpoints can be found in section 4.2

Primary endpoint

Null Hypothesis: The BE detection rate at the end of follow-up (excluding any found on random exit endoscopies) is the same in the intervention (Cytosponge™ -TFF3 test) arm and the control (usual care) arm.
Alternative hypothesis: The BE detection at the end of follow-up is not the same in the intervention (Cytosponge™ -TFF3 test) arm compared to the control (usual care) arm.

To determine whether the invitation to the Cytosponge™ -TFF3 test leads to an increase in the number of patients diagnosed with BE compared to the usual clinical care pathway in primary care we will compare BE diagnosis between intervention and the control i.e. usual care, arms in all patients entered into the study. We shall compare the proportions of BE between the two groups during follow-up using a generalised estimating equation (GEE) with BE diagnosis as the binary outcome comparing the

two arms as fixed effects with adjustment for age, gender, BMI, and length and dose of acid suppressive treatment together with cluster as a random effect. Briefly, the primary analysis will be a stratified test of proportions taking into account the variation inflation within each stratum (the individually randomised patients will account for a separate cluster with variance inflation factor equal 1). Let P0*s* and P1*s* be the proportions of patients with diagnosed BE for the two study arms in stratum *s*, and Vs the estimated variance of the difference in proportions in the stratum. The test statistic is the sum of (P1s – P0s)/ Vs and its variance is the sum of (1/ Vs). We will use both the assumed intra-class correlation (0.025) and the estimated intra-class correlation. Statistical significance will be based on a two-sided test with alpha equivalent to 5%. The primary analysis will be specified in advance in the Statistical Analysis Plan.

Secondary endpoints (all to be estimated together with (nominal) 95% Confidence Intervals):

(1) To estimate the diagnostic accuracy of the Cytosponge™ in primary care the sensitivity (proportion of true positives identified correctly) will be calculated as the proportion of all TFF3 positive results compared with endoscopy, where an endoscopy was carried out. The specificity (proportion of High-confidence negatives correctly identified) will be calculated as the proportion of TFF3 negative compared with negative endoscopy amongst the ~100 participants randomly invited to endoscopy from negative Cytosponge™ patients.
(2) To confirm the prevalence and incidence of BE and cancer stage diagnosis in the intervention and control arms for 10 years: Long term follow up data will allow Cox proportional Hazard model analysis to compare oesophageal cancer and death rates.
(3) The acceptability of both Cytosponge™ and endoscopy tests will be assessed as the proportion of patients willing to have the test amongst patients offered the test. 95% confidence intervals (Clopper-Pearson) will be used.

Where a full review is not possible, case note reviews will be performed on a limited (randomly selected) number of patients, which will vary according to the resources of each individual GP site. Patients found on case note reviews to have been entered onto the Trial without 6 months' worth of acid-suppressant medications will be retained in the final analysis based on the assumption that the automated search identified them because they did have a prescription for PPI/H2RA in the past. Besides, as the case note reviews are not performed on all of the participants, we would risk creating imbalances in the two arms if we were to exclude patients found to have less than 6 months' worth of PPI/H2RA.

Using the number of case-note reviews available in a practice as the denominator, we will calculate the proportion of patients in a practice that have less than 6 months' worth of acid-suppressant medications in the year preceding baseline. This proportion will be used as a threshold in our sensitivity analyses. However, practices recruited at the late stages of the Trial may not perform any additional case note reviews other than for patients picked up by the local coded search of their clinical information system due to time restrictions. In order to overcome these issues, we plan to perform three different sensitivity analyses:

Sensitivity analysis 1: Exclude the practices we know have **more than 20%** of patients with less than 6 months' worth of acid-suppressant medications as well as

those practices for which this information is unknown i.e. there has been **fewer than 20 patients whose notes have been reviewed manually**.

Sensitivity analysis 2: Exclude only the practices we know have **more than 20%** of patients with less than 6 months' worth of acid-suppressant medications.

Sensitivity analysis 3: Known individual patients with less than 6 months' worth of acid-suppressant medications will be **excluded** from the analysis; this will include patients whose notes have not been reviewed.

12.4 Economic evaluation

Primary endpoint: To gain an in-depth understanding of the health economics of the Cytosponge™ -TFF3 test we will undertake a detailed analysis of the cost and cost-effectiveness of the Cytosponge™ -TFF3 test versus usual care from the perspective of the NHS and personal social services. For the cost analysis cost components will include costs of diagnosis using the Cytosponge™ -TFF3 test, medication, endoscopies and biopsies, endotherapy, oesophagectomy, and follow-up in primary and secondary care. Volume of resource use data will be collected from practice records up until the end of follow-up. Unit costs will be taken from published sources such as NHS Reference Costs, the British National Formulary, and the Personal and Social Services Research Unit (PSSRU). Cytosponge costs and associated laboratory costs will be taken from the BEST3 trial. We will calculate mean (SD) and median (IQR) costs for both study arms for each cost component and all components combined.

Cost-effectiveness will be measured in terms of the incremental cost per QALY gained. We will adapt a previously-developed decision analytic model which compared the cost-effectiveness of two strategies: Cytosponge™ screening with confirmatory endoscopy, and treatment as usual, for 50-year-old men with GERD. The parameters for prevalence of BE, sensitivity and specificity of screening, uptake of the Cytosponge-TFF3 test, costs and utilities will be updated to reflect the BEST3 data. For these and all other parameters, including disease progression and management strategies, we will search the literature for updated evidence. If the data will allow it, we will have a look for any differences in rates of BE detection and resource use between men and women overall/in each study arm, and if significant, we will explore the effect of these on the model in our sensitivity analysis. We will also undertake value-of-information (VoI) analyses, based on the notion that investing in further research on probabilities of events, HRQL and costs will reduce decision uncertainty about the cost-effectiveness of the Cytosponge-TFF3 test. This will include both the expected value of perfect information (EVPI) and the expected value of partial perfect information (EVPPI). The latter focusing on individual model parameters or groups of parameters.

As well as looking at total costs, costs will be disaggregated by sector since this may affect implementation of the Cytosponge™ -TFF3 test (e.g. if the preponderance of costs are borne in primary care). Using epidemiological data on the national incidence of reflux predominant symptoms plus cost data from the present study we will also undertake a budget impact study to calculate what the total cost would be to the NHS if the Cytosponge-TFF3 test was rolled out nationally.

13 Data handling

13.1 Anonymous GP datasets

Each practice, once it has completed screening procedures and is entered onto the Trial, will be allocated a unique randomisation number by KCP CPTU. Any patients who are identified as eligible for BEST3 from the database search carried out by GP staff are given a unique identifier based on a combination of the GP surgery identifier and a number unique for the individual in this practice. This identifier will be noted in the screening log.

Patient data will be extracted from GP databases for all patients participating in BEST3 at entry into the study and at Study endpoint.

In primary care, Endpoint data will be extracted systematically by coded search and, where possible, by a manual review of randomly selected patients' records. To ensure good BE detection at Study endpoint, a coded search will be first run to identify relevant patients with new OGDs/referrals of Upper GI diagnoses during the study period for fuller review. Clinical data will be extracted from secondary care communications (held in GP records), mainly endoscopy and pathology reports and letters from specialist clinicians. A random sample of patients (depending on local capacity) will then be selected for fuller review of primary care data (mentioned before as 'case note reviews'). Additionally, where possible, an encrypted list of participants' NHS numbers will be linked to secondary care data and any additional information on BE diagnosis will be integrated to the final dataset.

Additionally, where possible, an encrypted list of participants' NHS numbers will be linked to secondary care data and any additional information on BE diagnosis will be integrated to the final dataset.

In order to collect primary endpoint data, i.e. BE and OAC diagnoses, at least one of three different data sources will be used:

1) Automated coded search carried out on the GP clinical record for all participants in all sites

2) Manual case-note review of the GP clinical record for all participants identified by the coded search + participants chosen at random in a number depending on the capacity of the sites, including all patients where practice opts to complete a full review

3) NHS number linkage with hospital records for a limited number of sites, depending on the availability of relevant hospital information systems that routinely capture relevant diagnoses and geographical proximity of site in relation to its linked hospital.

Where there is evidence of endoscopy from one of these sources, further details may be sought from clinical records. In particular,study-related endoscopies for participants with positive or other relevant Cytosponge™ results will be used.

Note that it is important to record the date up to which the coded search is looking for BE and OGD referrals. This date should be used at the date of last follow-up for that patient. (BE diagnosed after that date should not be included in the primary endpoint).

This is a schematic of how Methods 1 and 2 work together.

		**SECOND STEP: manual case-note review returns…**		
		**BE diagnosis**	**Negative**	**Not done**
**FIRST STEP: automated coded search returns…**	**BE diagnosis**	For all patients with a BE diagnosis picked up in the coded search	N/A	N/A
	**OGDs/EAC/referral**	Some of the patients with an OGD/EAC/referral will have a BE diagnosis when their record is reviewed.	Some of the patients with an OGD/EAC/referral will not have any BE diagnoses when their record is reviewed.	N/A
	**Negative**	A randomly selected number of patients who have not been identified by the coded search will have their records reviewed, which will show a BE diagnosis.	A randomly selected number of patients who have not been identified by the coded search will have their records reviewed and no BE diagnoses will be found.	Most patients not identified by the coded search will not have their records reviewed manually.

The primary endpoint will be manual case note confirmed BE (column "BE diagnosis" in the Table). Patients negative on review or with no manual review and negative on automated search will be considered not to have BE. All patients with OGD/EAC referral should have a manual review; if they have not had a manual review, the result of the review will be considered as missing and handled through multiple imputations.

Note that, for some of the sites whose follow-up period finishes towards the end of the study (patient-level randomised group), there may be no manual search of patients negative on the automated search, i.e. no manual review of randomly-selected patients, due to time restrictions.

Approximate balance in the number of full case note reviews performed at random should be ensured between the two arms for practices in the cluster randomised group.

Patients with BE detected following a positive Cytosponge™ test result will be treated as any other patient, e.g. they will not have their record manually reviewed if their BE diagnosis and/or OGD referral was not coded in their local practice unless their record is picked up at random for a manual case note review.

At Study endpoint for patients who have not opted out but not provided consent (at point of Cytosponge™ test), the practice team will enter primary care data on a participant-level basis and secondary care events (a referral/OGD, or a BE, dysplasia or cancer diagnosis) in aggregate form only to avoid inadvertent identification once the data have been transferred to the central trial team. Patients who have provided consent for the use of identifiable data will have all data collected on a participant-level basis.

13.2 Case report form

Data will be recorded directly to a database using online Electronic Case Report Forms (eCRFs) i.e no local source data. The eCRFs will be managed by a secure web application, accessible via HTTPS/SSL. Where paper CRFs are additionally used, this documentation will be added to the patient's clinical record. CRFs completed as part of pathology review will use both paper and eCRF. Users will be issued with a username and password and will be required to login for web application access; their activity will be tracked using unique user identities and their access to data controlled by defined access roles. Users should not share account details. Personal Identifiable Data will be encrypted in the database and kept separately from the clinical data. Direct access to the database will be restricted to named users only.

A paper backup system will be established in case of technical failure or for local convenience. Where paper CRFs are used, they should be kept in the Investigator Site File and they will be reviewed as part of source data verification during site monitoring. Patients will be identified only by initials, trial number and year of birth.

The eCRFs will be completed by the Investigator or suitably trained research staff, as designated in the site delegation log, as accurately and completely as possible throughout the study. If, after screening, a patient is found to be ineligible for the Cytosponge™, the date of the screening phone call and reason for non-participation will be added to their pseudo anonymous record on the BEST3 application.

Unless paper CRFs have been used, the BEST3 App will constitute the primary data source for the study, and will be fully accessible by the Central Coordinating Office at KCL CPTU.

13.3 Record retention and archiving

During the course of the research, all records are the responsibility of the Chief Investigator, and must be kept in secure conditions. When the Trial is complete, it is a requirement of the UK Policy Framework for Health and Social Care Research and Queen Mary University of London/King's College London policy that the records are kept for a further 20 years. Site files from other sites must be archived at the local repository for a minimum of five years or as per local practice.

14 Trial Committees

Data Monitoring Committee

The independent Data Monitoring Committee (DMC) will review the trial data and advise the sponsor (directly or indirectly) on the future management of the Trial. Its membership will include two independent clinicians and an independent statistician. The DMC will review quality and compliance data, as well as safety and efficacy. They are privy to interim comparisons by arm and see data in a format not to be shared beyond its independent members, except for the Trial Statistician who will provide the data. Monitoring of the data will be undertaken every 6 months and summary analyses will be provided to the DMC. No formal interim analyses are planned.

The DMC will meet once before the Trial starts and at appropriate intervals as determined by the committee, but at least once a year. They will advise the TSC, and may recommend early closure of the Trial or discontinuation of any research arm as deemed necessary. Given that this is not a high-risk trial and that there will not be any early stopping rule, recommendations regarding premature termination of the study will be at the discretion of the DMC and TSC, but will be the decision of the TMG.

Trial Steering Committee (TSC)

The aim of the TSC is to provide overall supervision for the Trial on behalf of the Trial Sponsor and Trial Funders, and to ensure that the Trial is conducted to the rigorous standards set out in the Medical Research Council's (MRC) Guidelines for Good Research Practice. The TSC will concentrate on progress of the trial, adherence to the protocol, patient welfare and consider new information of relevance to the research question. The TSC will act on the guidance of the Data Monitoring Committee (DMC) to provide advice, through its chair, to the Chief Investigator, trial sponsor, Trial funders, KCL, QMUL and any other relevant party on all appropriate aspects of the Trial. The majority of members of the TSC, including the Chair, are independent of the Trial. Non-independent members are also part of the TSC. Representatives of the trial sponsor and the Trial funders will also be invited to all TSC meetings. The final decision regarding whether or not the trial may continue is the responsibility of the TSC.

Trial Management Group (TMG)

The TMG oversee the progress of the Trial and act on the advice of the TSC. Amongst its members are the lead investigators (clinical and non-clinical), trial co-ordinators, and staff from the Centre for Cancer Prevention. The TMG is responsible for the day-to-day running and management of the Trial.

15 Monitoring, audit and inspection

Monitoring of the trial will be conducted using a risk-based approach and follow a trial monitoring plan developed by the KCL CPTU team through data review and site visits on an agreed frequency and schedule

The following areas will be reviewed on a regular basis:

- Participant enrolment, consent and eligibility
- Adherence to trial interventions and policies to protect participants, including reporting of harm
- Completeness, accuracy, and timeliness of data collection
- Device accountability and handling
- HTA compliance

Sites eligible for monitoring would be those with the highest enrolment rates, large numbers of withdrawals, low numbers of Cytosponge™ uptake, or atypical (low or high) numbers of reported ADEs. Sites would be required to accommodate monitoring visits by providing access to relevant staff, premises and records. Monitoring findings will be reported to the Chief Investigator, TSC and Sponsor as required. Further details will be elaborated upon in the Trial Monitoring Plan.

16 Ethical and regulatory considerations

16.1 Health Research Authority (HRA) and Research Ethics Committee (REC) approvals

The Chief Investigator will ensure that the protocol and supporting participant-facing documentation receive HRA Approval, including being presented to a relevant Research Ethics Committee for approval. Following ethical review, research will only take place once appropriate HRA approvals are in place. The KCL study team will prepare the Annual Progress Report on behalf of the Chief Investigator within 30 days of the anniversary date on which the favourable opinion was given, and annually until the Trial is declared ended. They will also on behalf of the Chief Investigator:

- notify the REC of the end of the study
  will notify the REC, including the reasons for the premature termination if required within one year after the end of the study, submit a final report with the results, including any publications/abstracts, to the REC.

16.2 Notification of No Objection from MHRA

Appropriate approvals will be sought to receive a Notification of No Objection from the MHRA to use the device in the Trial. All reporting to the MHRA will follow the Sponsors' Standard Operating Procedures.

16.3 Peer review

The Trial design was peer reviewed as part of a competitive project grant application with CRUK. The peer review was independent, expert, and proportionate with 5 reviews.

16.4 Regulatory compliance

The Study will not commence without the necessary regulatory and organisational approvals being in place. The Study will have appropriate HRA Approval and site confirmation of capacity and capability in place for GP and hospital sites

The Trial will take place in primary and secondary care and as such, the study team will seek advice from the Sponsor with regard to the necessary organisational approval for the study to take place within their respective organisations. The Trial Master File will hold all relevant communications with regulatory bodies and maintained by KCL. Appropriate approval will be sought from MHRA.

16.5 Protocol compliance

Any accidental protocol deviations will be adequately documented on the relevant forms and reported to the Chief Investigator and Sponsor. All deviations from the protocol which are found to frequently recur will require immediate action and could potentially be classified as a serious breach.

Notification of Serious Breaches to GCP and/or the protocol: A “serious breach” is a breach which is likely to affect to a significant degree

- the safety or physical or mental integrity of the subjects of the Trial; or
- the scientific value of the Trial

The Sponsor will be notified immediately of any case where the above definition applies during the trial conduct phase.

16.6 Data protection and patient confidentiality

All investigators and trial site staff will comply with the requirements of GDPR and the Data Protection Act 2018 with regards to the collection, storage, processing and disclosure of personal information and will uphold the Act’s core principles. Permission for the transfer, storage and use of person identifiable data (PID) in the Trial will be provided by consenting participants. PID will only be collected for participants who have consented to take part in the intervention part of the protocol. This will enable the research team to communicate directly with patients regarding relevant matters e.g. lack of engagement from GP, arranging appointments, newsletter if available etc.

Individually-consented participants will consent to the University of Cambridge and QMUL receiving, transferring, storing and using their personal identifiable data for the purposes of this study. PID will be stored on secure networks in Cambridge and QMUL. All electronic transfers of person-identifiable data will meet industry and NHS-mandated standards including encryption to at least AES 256.

Data will be retrievable on the BEST3 App in a linked anonymised manner in which where the participant’s identifying information is replaced by an unrelated code. The participant’s Anonymous flagging of cancer status with the UK Cancer Registry may be undertaken as suitable models become available in 2017-2018. Individual level flagging consent will be gained to ensure the availability of data for future research aims, and in line with CRUK funding conditions and longer-term monitoring of device-related safety

PID will be stored in a logically-separated system with limited access to central team individuals for quality control, audit, analysis and communications. All paper-based PID will be securely handled and maintained in controlled access locations and following local NHS policies and procedures for information security. All samples leaving practices and NHS trusts and received by the lead site and third party laboratories will carry a unique identifier and may include date of birth (in line with patient consent) to enhance quality assurance.

Participants will be aware that their PID may be accessed by external regulatory bodies for the purposes of assessing legal compliance and meeting relevant regulatory obligations. They will be made aware that external health care professionals may access data for the purpose of second opinion on clinically-relevant findings. PID will be stored for 10 years following completion of the study. All research data will be stored for a period of 5 years after the end of the grant in line with CRUK funding requirements.

For future research, genetic data, including whole genome sequencing data, may be transferred and stored in international repositories for access by international researchers in line with patient consent.

16.7 Financial and other competing interests for the Chief Investigator, PIs at each site and committee members for the overall trial management

Rebecca Fitzgerald and Maria O'Donovan are named inventors of the Cytosponge™ technology. The Trial Steering Committee (TSC) will ensure appropriate oversight with regard to data quality, trial conduct and safety reporting, and report to CRUK as the funder and other stakeholders as required. Medtronic (sub-contractor Europlaz) as the licensee of the Cytosponge™ technology (from Medical Research Council Technology) is providing Cytosponge devices and related consumable cost free to the Trial. All Principal Investigators joining the Trial over the course of Trial will disclose any competing interests to the TSC.

16.8 Insurance and indemnity arrangements

The Trial's Sponsors will ensure that appropriate insurance and indemnity arrangements are in place for the study.

- The University of Cambridge is providing insurance coverage to cover any claims that could result from the design of the research study via protocol insurance.
- If harm occurs to the patient because the device has not been manufactured in accordance with the specification, this will be covered by Europlaz's insurance.
- Cambridge University Hospitals NHS Foundation Trust under the NHS Indemnity Scheme provides an indemnity for any failure to comply with the requirements of the manufacturer in accordance with MHRA requirements.
- Clinical staff holding NHS contracts (substantive or honorary) will be covered for harm caused to participants under the NHS Indemnity Scheme.
- Nurses and GPs employed by their GP practice will be covered by indemnity arrangements in place at their practice as confirmed by the GP practice.

16.9 Amendments

Study amendments will be prepared by the KCL CPTU study team for submission according to Sponsor, HRA and MHRA requirements. Both Cambridge and KCL

teams, and the Trial Steering Committee will approve protocol changes prior to submission. It will be the Sponsors' responsibility to decide whether an amendment is substantial or non-substantial for the purposes of submission to regulatory bodies. KCL will be responsible for cascading amendments to participating sites.

16.10 Access to the final trial dataset

During trial implementation, the main study team will have controlled access to the full dataset. Wider data-sharing with follow the data-sharing and dissemination plans agreed with the funder CRUK.

17 Public and Patient Involvement

Public and Patient Involvement (PPI) for this study is occurring at different levels.

17.1 Cytosponge™ acceptability study

10 interviews and 4 focus group with GERD patients were conducted to obtain a deeper understanding of how potential participants would react to an invitation to the BEST3 Trial. Furthermore, we investigated how the idea of having this sponge procedure would be perceived and any preferences regarding presentation of the procedure in study documents. This study showed that the overall acceptability was high, but there was initial concern about the physical experience of having the test, including swallowing and extracting the Cytosponge™. These worries were reduced after handling the device and seeing a video demonstration of the procedure. The data obtained to guide the development of information materials for the BEST3 Trial.

17.2 Involvement of PPI representatives in the study design

The BEST3 study design and key documents (introductory letter, Cytosponge™ leaflet, and PIS) were discussed with a group of 7 PPI representatives based at Cambridge University Hospitals. This group will also review all patient facing materials, specifically patient information sheets (PIS) and consent forms. Their advice will be used to further guide the design of the study and study materials.

17.3 Further involvement of PPI representatives

Members of the Cambridge PPI study design group will take part in the BEST3 TSC. As part of the TSC, they will be involved in the remainder of the Trial, including analysis of results and dissemination of findings. Other representatives may join as required.

18 Dissemination policy

18.1 Sharing of datasets

It is not expected that the data will be shared until the primary study results have been published by the main study team so as not to compromise the publication plan. Results are expected to be published in at least two peer reviewed articles at the end of the study. The aims will be for at least one of them to be published in a top tier medical journal.

The recruitment period of the study is 18 months with primary endpoints of diagnosis of BE and health economics outcome being complete at the end of the recruitment period. Six months have been planned for data analysis, consolidation, preparation for sharing (including preparation of any relevant supporting documentation), and publication of the main outcomes.

All publications relating to trial data will be jointly approved by Cambridge and KCL prior to submission. The final trial report will be jointly authored by the University of Cambridge and KCL as set out in their collaborative agreement dated February 2019. CRUK as trial funder will be acknowledged in all publications.

Data may be shared bilaterally with external organisations. Data recipients may be required to sign a data sharing agreement which describes the conditions for release and requirements for data transfer, storage, archiving, publication and Intellectual Property. Formal requests for data sharing are considered in line with University of Cambridge and KCL/QMUL policy with due regard given to funder and sponsor guidelines. Requests are via a standard pro forma describing the nature of the proposed research and extent of data requirements. Data sharing will be in accordance with the UCAM and QMUL CCP Policy on Sharing Personal Data. This policy is in line with UK CRC guidance on data sharing which is approved by Cancer Research UK and which is also in line with the requirements issued by the Information Commissioner in the “Data Sharing Code of Practice” under the Data Protection Act 1998 (DPA) to be considered when sharing personal data with Data Processors and with other Data Controllers.

Participants will be made aware of the sharing of anonymised research data with commercial bodies and other research organisations in the UK and overseas. Data may be shared with commercial organisations including for the purposes of research and development, and for commercialisation activities.

Requests for data will be reviewed by the BEST3 Data Access Committee (DAC) in terms of scientific merit and ethical considerations including patient consent. Data sharing is undertaken if proposed projects have a sound scientific or patient benefit rationale as agreed by the Data Access Committee Whilst the Trial Steering Committee (TSC) are active they will approve the membership of the DAC which will typically consist of the CI, the Trial Statistician and an independent member of the TSC. The TSC will also ensure that a suitable DAC is in place when the TSC is disbanded. The post-trial DAC will most likely be the standing BEST3 DAC with the addition of the BEST3 CI. The TSC provides expert independent oversight of a study, or a number of study/trials within a defined area of research, on behalf of sponsors and funders. The TSC works to a charter issued by KCL CPTU that describes its composition, responsibilities and decision-making powers.

External users will be bound by the data sharing agreements according to institutional policy “sharing personal data under the Data Protection Act 1998 (Minute reference OPS/8/12/16: approval date 28.8.12). This policy is in line with the requirements issued by the Information Commissioner in the “Data Sharing Code of Practice” under the Data Protection Act 1988 (DPA) to be considered when sharing personal data. Data recipients are required to sign a data release form which describes the conditions for release.

18.2 Discoverability of dataset

The study will be listed on The Wolfson Institute of Preventive Medicine’s website and MRC Cancer Unit websites. These sites include summary information on study design and endpoints. The full trial dataset may also be deposited in a range of third-party discovery repositories to enhance secondary use of the data. Patients will be made aware of this broad sharing.

18.3 IP considerations

All IP resulting from the trial data will be managed in line with the Cambridge-QMUL-KCL collaborative agreement dated February 2019 and in line with overarching IP arrangements between CRUK and the University of Cambridge and between Medtronic, University of Cambridge and the Medical Research Council as IP owner of the Cytosponge™ technology.

18.4 Authorship guidelines

The final trial report, and related publications, will be authored in line with the arrangements set out in section 6.0 of the Collaborative Agreement dated February 2019. All publications will be approved by the Chief Investigator. Authorship will follow the criteria established by the International Committee of Medical Journal Editors.

19 References

1. Pohl, H., B. Sirovich, and H.G. Welch, *Esophageal adenocarcinoma incidence: are we reaching the peak?* Cancer Epidemiol Biomarkers Prev, 2010. **19**(6): p. 1468-70.
2. Donaldson, L., *On the State of Public Health: Annual Report of the Chief Medical Officer 2007.* 2008. **http://www.dh.gov.uk/en/Publicationsandstatistics/Publications/AnnualReports/DH_086176**: p. Accessed 20/01/2009.
3. NICE, *Dyspepsia and gastro‑oesophageal reflux disease: Investigation and management of dyspepsia, symptoms suggestive of gastro‑oesophageal reflux disease, or both.* 2014(https://www.nice.org.uk/guidance/cg184).
4. Shawihdi, M., et al., *Variation in gastroscopy rate in English general practice and outcome for oesophagogastric cancer: retrospective analysis of Hospital Episode Statistics.* Gut, 2014. **63**(2): p. 250-61.
5. Cameron, A.J., et al., *Prevalence of columnar-lined (Barrett's) esophagus. Comparison of population-based clinical and autopsy findings.* Gastroenterology, 1990. **99**(4): p. 918-22.
6. Vaughan, T.L. and R.C. Fitzgerald, *Precision prevention of oesophageal adenocarcinoma.* Nat Rev Gastroenterol Hepatol, 2015. **12**(4): p. 243-8.
7. Dent, J., et al., *Epidemiology of gastro-oesophageal reflux disease: a systematic review.* Gut, 2005. **54**(5): p. 710-7.
8. Campaign, A., *Be Clear on Cancer.* https://www.gov.uk/government/news/be-clear-on-cancer-campaign-highlights-links-between-heartburn-and-cancer, 2015.
9. NICE, *Endoscopic radiofrequency ablation for Barrett's oesophagus with low‑grade dysplasia or no dysplasia.* NICE interventional procedure guidance [IPG496], 2014(http://www.nice.org.uk/guidance/ipg496/chapter/1-recommendations).
10. Lao-Sirieix, P., et al., *Non-endoscopic screening biomarkers for Barrett's oesophagus: from microarray analysis to the clinic.* Gut, 2009. **58**(11): p. 1451-9.
11. Ross-Innes, C.S., et al., *Novel insights from whole genome sequencing on clonal heterogeneity in Barrett's carcinogenesis.* Nature Genetics, 2015. **In Press**.

12. Kadri, S.R., et al., *Acceptability and accuracy of a non-endoscopic screening test for Barrett's oesophagus in primary care: cohort study.* BMJ, 2010. **341**: p. c4372.
13. Ross-Innes, C.S., et al., *Evaluation of a Minimally Invasive Cell Sampling Device Coupled with Assessment of Trefoil Factor 3 Expression for Diagnosing Barrett's Esophagus: A Multi-Center Case–Control Study.* PLoS Med, 2015. **12**(1): p. e1001780.
14. Lao-Sirieix, P., et al., *Non-endoscopic immunocytological screening test for Barrett's oesophagus.* Gut, 2007. **56**(7): p. 1033-4.
15. Lao-Sirieix, P., et al., *Evaluation of a minimally-invasive cytosponge esophageal cell collection system in patients with Barrett's esophagus.* Gastroenterology, 2015. **148**(4): p. S16.
16. Benaglia, T., et al., *Health benefits and cost effectiveness of endoscopic and nonendoscopic cytosponge screening for Barrett's esophagus.* Gastroenterology, 2013. **144**(1): p. 62-73 e6.
17. Craig, P., et al., *Developing and evaluating complex interventions: new guidance.* www.mrc.ac.uk/complexinterventionsguidance, 2008.
18. Sim, J. and A. Dawson, *Informed Consent and Cluster-Randomized Trials.* American Journal of Public Health, 2012. **102**(3): p. 480-485.
19. Jones, R., K. Coyne, and I. Wiklund, *The Gastro-oesophageal Reflux Disease Impact Scale: a patient management tool for primary care.* Alimentary Pharmacology & Therapeutics, 2007. **25**(12): p. 1451-1459.
20. Ross-Innes, C.S., et al., *Evaluation of a minimally invasive cell sampling device coupled with assessment of trefoil factor 3 expression for diagnosing Barrett's esophagus: a multi-center case-control study.* PLoS Med, 2015. **12**(1): p. e1001780.
21. Lerman, C., et al., *Psychological side effects of breast cancer screening.* Health Psychol, 1991. **10**(4): p. 259-67.
22. Marteau, T.M. and H. Bekker, *The development of a six-item short-form of the state scale of the Spielberger State-Trait Anxiety Inventory (STAI).* Br J Clin Psychol, 1992. **31 (Pt 3)**: p. 301-6.
23. Kadri, S.R., et al., *Acceptability and accuracy of a non-endoscopic screening test for Barrett's oesophagus in primary care: cohort study.* 2010. **341**.
24. Schoen, R.E., et al., *PAtient satisfaction with screening flexible sigmoidoscopy.* Archives of Internal Medicine, 2000. **160**(12): p. 1790-1796.

Appendix 1: List of abbreviations

ADE	Adverse Device Event
AES	Advanced Encryption Standard
BE	Barrett's oEsophagus
Barts CTU	Bart's Clinical Trials Unit
CCG	Clinical Commissioning Group
CI	Chief Investigator

CPTU	Cancer Prevention Trials Unit
CRF	Case Report Form
CRN	Clinical Research Network
CRUK	Cancer Research UK
CTA	Clinical Trial Authorisation
DMC	Data Monitoring Committee
EAC	oEsophageal Cancer
EMIS	Egton Medical Information Systems
GCP	Good Clinical Practice
GERD	Gastro-oEsophageal Reflux Disease
GI	Gastro-Intestinal
GMP	Good Manufacturing Practice
GP	General Practitioner
HRA	Health Research Authority
DMC	Data Monitoring Committee
IG	Information Governance
ISRCTN Trials	International Standard Randomised Controlled Number
KCL	King's College London
NPV	Negative Predictive Value
PI	Principal Investigator
PIS	Participant Information Sheet
PPI	Proton Pump Inhibitors
PPV	Positive Predictive Value
QA	Quality Assurance
QC	Quality Control
RCT	Randomised Control Trial
REC	Research Ethics Committee
SADE	Serious Adverse Device Event
SOP	Standard Operating Procedure

SSI	Site Specific Information
TFF3	TreFoil Factor 3
TSC	Trial Steering Committee

Appendix 2: Risk Assessment

Risks associated with trial interventions
☒ LOW ≡ Comparable to the risk of standard medical care
☐ MODERATE ≡ Somewhat higher than the risk of standard medical care
☐ HIGH ≡ Markedly higher than the risk of standard medical care

Justification: Briefly justify the risk category selected and your conclusions below (where the table is completed in detail the detail need not be repeated, however a summary should be given):

The Cytosponge™ has been assessed in previous research studies to be safe and effective. 2000 participants have received the device with no serious adverse events attributed to it. See KCL CPTU Trial risk assessment for more details

What are the key risks related to interventions you plan to monitor in this trial?		How will these risks be minimised?		
Intervention	Body system/Hazard	Activity	Frequency	Comments
Cytosponge™ procedure	Upper GI Tract	Review of eligibility and contraindications, training of nurses, 7 day safety telephone call, alert card	Single visit (up to 2 attempts)	
Cytosponge™ procedure	Detachment in stomach	Training of nurses in emergency procedure	At study set up and throughout	

Outline any other processes that have been put in place to mitigate risks to participant safety (e.g. DMC, independent data review, etc.)

The DMC will be monitoring safety data relating to the Trial including safety incidents, trends, and patterns and will independently advise the TSC on ongoing implementation.

Appendix 3: Table of procedures

Procedures	Pre-procedure	Cytosponge™ visit	+7-14 days of procedure	+6 weeks of procedure	+12 weeks of procedure	At approximately 12 months (9-15 months)
BEST3 Data collection letter	X					
Cytosponge™ leaflet and reply slip with one reminder	X					
Telephone call to further assess eligibility and arrange appointment	X					
STAI-6	X		X			
Eligibility criteria		X				
Informed consent		X				
Weight and height measurement		X				
Clinical/demographic data		X				
GERD questionnaire		X				
Cytosponge™ procedure		X				
Lifestyle/family history		X				
Perceived risk of oesophageal cancer		X	X			
Inventory to Assess Patient Satisfaction			X			
Visual Analogue Scale			X			
Phone call to assess ADEs			X			
Clinical feedback				X		
Confirmatory endoscopy - positive result					X	
Saliva sample					X	
Invitation to research endoscopy – Usual care						X
Invitation to research endoscopy – Negative result						X
Invitation to research endoscopy – Non responders						X

Appendix 4: Safety Reporting Flow Chart

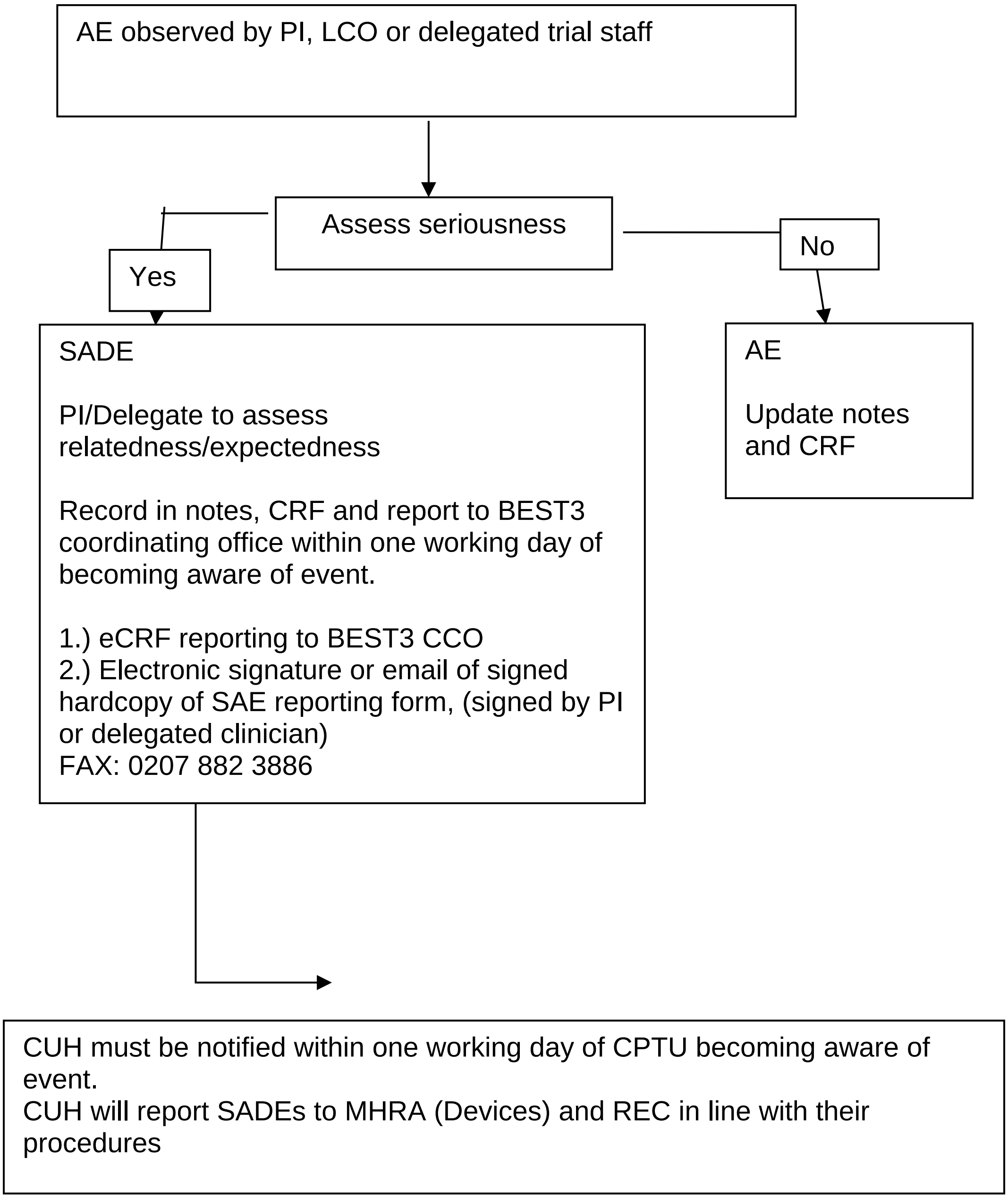

Appendix 5: Amendment History

Amendment No.	Protocol version no.	Date issued	Author(s) of changes	Details of changes made
Pre approval	v1.1	28 November 2016	Beth Muldrew	Request by MHRA to amalgamate Clinical Investigation Plan and protocol
Pre-approval	v1.2	5 January 2017	Beth Muldrew	• Change from 14 to 30 day opt out period<br>• Saliva collection for positive cases at endoscopy only<br>• Strengthening of indemnity and insurance sections.
Pre-approval	v1.3	23 January 2017	Beth Muldrew	• Clarification regarding risk of inhalation and medical cover
Amendment 2 (Amendment 1 non-protocol)	v1.4	20 March 2017	Beth Muldrew	• Removal of Clopidogrel as exclusion<br>• Change from 3 Cytosponge™ attempts to 2.<br>• Clarification regarding baseline data requirements<br>• Removal of Spire Pathology Services and replacing of CUHTB<br>• Changes to SAE reporting to fall in line with App development<br>• Change from written agreement to ' confirmation' prior to randomization

				• Addition of wording regarding Xylocaine to reflect patient information sheet
Amendment 3	v1.4	20 March 2017	Beth Muldrew	• Change from 30 day opt out period to 14.
Amendment 4	v2.0	15 July 2017	Beth Muldrew	• Addition of wording around Female:Male ratio<br>• Addition of wording to allow for practice cohort size to increase<br>• Addition of wording explaining rationale for including participants who have had an Upper GI endoscopy and this is<br>• Addition of formal reminder step<br>• Clarification regarding timing of STAI-6 at baseline<br>• Addition of wording about the provision of Lidocaine<br>• Changes to classification of results and re-testing
Amendment 6	v3.0	Feb 2018	Michelle Sleeth and Beth Muldrew	• Removal of reference to specific stratum for practice size<br>• Addition of patient-level randomisation procedure<br>• Updated power calculations to

				reflect addition of patient-level randomisation<br>• Update that 10% of original total sample size (i.e. n=800) will be invited for research endoscopies.<br>• Changes to reflect that 12 month (average) follow up will be used)<br>• Update to study summary timeline<br>• Change to investigators institutional affiliations<br>• Addition of information that primary endpoint data will also be collected from endoscopy records for the usual care arm<br>• Update that patients not found to have 6 months of PPI therapy at Baseline may still be eligible<br>• Clarification that patients with dysplasia or above will receive standard clinical letters rather than research<br>• Additional information added on how follow up data will be obtained at the 12M (average) follow up point using

				encrypted NHS numbers.
Amendment 8	v3.1	June 2019	Benoit Aigret, Roberta Maroni, Beth Muldrew, Nick Swart, Peter Sasieni, Rebecca Fitzgerald	• References to QMUL/KCL clarified<br>• List of key trials contacts amended.<br>• Update on sample size figures in Trial Summary table.<br>• Cytosponge™ has become CE marked.<br>• Number of research endoscopy invitations offered across both arms amended to 500.<br>• Additional references to 12 months of follow-up have been amended to take into account the variable length of follow-up.<br>• Addition of a section on current uptake and consequences on power<br>• Addition of a section to explain variable follow-up periods<br>• Added updated methods of data collection for the Primary endpoint

Appendix 3 - MHRA approval to amendment in study design

Ms Beth Muldrew
Cambridge University Hospitals NHS Foundation Trust
Barts Clinical Trials Unit
Centre for Cancer Prevention
Queen Mary University of London
Cambridge Biomedical Campus
EC1M 6BQ
United Kingdom

Our ref: CI/2016/0057

Your ref: Amendment 8 (REC Amendment 6)

31 May 2018

Dear Ms Muldrew

CLINICAL INVESTIGATION
AMENDMENT [7] - NO OBJECTION

Manufacturer : Cambridge University Hospitals NHS Foundation Trust
Model Name : BEST3 Cytosponge
Description : "Barrett's Oesophagus Cytosponge Test Kit "

Thank you for your letter dated 20th February 2018 informing us of your intention to make the following amendments as detailed within the letter to the;

1. Randomisation method
2. Additions of the following sites
3. Changes to the Investigator brochure
4. Changes to patient information

Following the teleconference that took place on 31st May 2018 we are writing to inform you that the Competent Authority has no objection to the changes described in your letter with the following recommendations/conditions which are as follows;

1) MHRA recommends that the two parts of the study (pre and post individual randomisation) should be analysed separately based on methods that respect the structure of the data.
2) MHRA recommends to also estimate treatment effect based on a combined analysis from the two parts of the study (pre and post randomisation). A combined analysis should only be performed if the results from the two parts of the study favour the study test.

If you have not done so already and prior to implementing this study amendment MHRA require a copy of the Protocol/Clinical Investigation Plan authorisation page, with all the required signatures.

Please note that the amendment must not be implemented until the relevant Ethics Committee approval has been obtained. Ethics Committee approval forms part of the information required under Section 2.2 of Annex VIII, therefore in light of this please confirm that Ethical approval has been granted for this amendment and provide MHRA with a copy of this as soon as it becomes available.

If you have not done so already you are also required to obtain approval for the new investigation site(s) (for NHS sites this approval must be obtained from the NHS/HSC R&D office and for non-NHS sites this approval must be obtained from your ethics committee). Please notify MHRA of the outcome of the relevant approvals for each new site once received. Please note that this clinical investigation must **not** commence in any of the new UK sites until you have received the relevant approvals for that individual site and you have notified MHRA of this.

May I also take this opportunity to remind you that in the event of a serious adverse incident occurring during the course of the clinical investigation you should inform the MHRA Adverse Incident Centre in line with the requirements in our letter of No Objection.

Yours sincerely,

Sean Williams
(on behalf of the Competent Authority)

Tel: +44 (0)20 3080 7325
Email: sean.williams@mhra.gov.uk

Appendix 4 - BEST3 Statistical Analysis Plan

Cancer Prevention Trials Unit (CPTU)
Cancer Prevention Group School of Cancer & Pharmaceutical Sciences
King's College London
cptu@kcl.ac.uk
Director: Professor Peter Sasieni

Statistical Analysis Plan (SAP)

**Title**	BEST3 Statistical Analysis Plan		
**Reference**	BEST3 SOP 008	**Version Number**	**1.0**
**Approval Date**	16/12/2019	**Effective Date**	30/12/2019
**Review Date**	30/12/2022		

	**Name**	**Position**
**Authors**	Roberta Maroni<br>Marcel Gehrung<br>Judith Offman	Trial Statistician<br>Statistician<br>Trial Epidemiologist

**Approved by**	Peter Sasieni<br><br>Name	Director of CTU/Senior Statistician<br><br>Position
	Signature	16/12/2019<br><br>Date

If this SAP has been printed or saved electronically, please check Sharepoint to ensure this version is the most up-to-date - https://emckclac.sharepoint.com/sites/LSMcpg

**Version**	**Date Approved**	**Reason for Change**	**Author**
1.0	XX/XX/2019	NA	

Table of Contents

Abbreviations 3
1. Purpose and objective 4
2. Study objectives and design 4
2.1 Primary endpoint 6
2.2 Secondary endpoints 6
2.3 Assessment of objectives 7
Assessment of primary endpoint 7
Assessment of secondary endpoints 9
2.4 Level of significance 13
2.5 Sample size 13
2.5.1 Changes to study design after Milestone 1 review 13
2.5.2 Sample size calculations 14
2.5.3 Lower uptake of the Cytosponge™ invitation 16
2.5.4 Variable follow-up periods 16
2.5.5 Randomisation algorithm 17
3. General analysis definitions 18
3.1 Study periods 18
3.2 Study populations 18
3.2.1 Intention-to-treat population 18
3.2.2 Per-protocol population 19
3.2.3 Non-compliance corrected (ITT) population 19
3.2.4 Safety population 20
3.3 Subgroup definitions 20
3.4 Treatment assignment and treatment groups 20
4. Patient disposition 21
4.1 Compliance to the Cytosponge™-TFF3 test 21
4.2 Compliance to confirmatory endoscopies 21
4.3 Compliance to research endoscopies (after end of follow-up) 22
5. Demographics and baseline characteristics 22
5.1 Characteristics collected during the study 23
5.2 End-of-study data 23
5.3 Prior medications and treatments 23
6. Interim analysis and timing for analysis 23
6.1 Interim analysis 23
6.2 Time-points for analysis 24
7. Efficacy analysis 24
7.1 Method for analysis of endpoints 24
7.1.1 Analysis of primary endpoint 24
7.1.2 Analysis of secondary endpoints 27
7.1.3 Analysis of further subgroups 31
7.2 Covariates 31
7.3 Methods for handling missed data and outliers 31
7.3.1 Handling of dropouts 31
7.3.2 Handling of missing data in active subjects 31
8. Safety analysis 32
8.1 Summary of adverse events 32
8.1.1 Number of adverse events 32
8.1.2 Number of patients affected by an adverse event 32
8.2 Analysis of adverse events 32
8.3 Summary of Serious Adverse Events (SAE) 32
8.4 Analysis of SAE 33
9. Presentation of analysis 33
9.1 Reporting of results 33
9.2 Presentation of results 34
10. References, related SOPs, web links 34
11. Appendices and associated documents 35

Abbreviations

AE	Adverse Event
BE	Barrett's oEsophagus
BMI	Body Mass Index
BOC	Benign Oesophageal Condition
CLR	CLuster Randomised
CRF	Case Report Form
DMC	Data Monitoring Committee
EAC	Oesophageal AdenoCarcinoma
GI	GastroIntestinal
GP	General Practitioner
H2RA	Histamine-2 Receptor Antagonists
IM	Intestinal Metaplasia
IQR	Interquartile Range
ITT	Intention-To-Treat
MHRA	Medicines and Healthcare products Regulatory Agency
NHS	National Health Service
NPV	Negative Predictive Value
OGD	Oesophago-Gastro-Duodenoscopy
PLR	Patient-Level Randomised
PPV	Positive Predictive Value
RCT	Randomised Controlled Trial
SAE	Serious Adverse Event
SAP	Statistical Analysis Plan
STAI	Spielberger State-Trait Anxiety Inventory
TFF3	Trefoil Factor 3
VIF	Variance Inflation Factor

1. Purpose and objective

This document contains the **Statistical Analysis Plan** (SAP) for the BEST3 study. The SAP is required by the National Institute of Health to improve reproducibility, transparency and validity of clinical trials.

The table of contents of this SAP follows the one recommended in SOP Barts CTU GEN ST 01 “Statistical Analysis Plan”, version 4.0.

2. Study objectives and design

The BEST3 study is a 1:1 randomised controlled trial (RCT) where consented patients are either recruited to usual care or to receive an invitation to the **Cytosponge™-Trefoil Factor 3** (TFF3) test, a novel non-endoscopic device whose purpose is to detect Barrett’s oEsophagus (BE), a pre-cancerous lesion of oesophageal cancer.

Subjects recruited in the BEST3 study are either cluster randomised (CLR), i.e. General Practitioner (GP) practices are the units of randomisation, or ‘patient-level’ randomised (PLR), i.e. patients are individually randomised to either the intervention or the control arm. This second type of randomisation was added to the study at a later time because of concerns in low recruitment numbers and it was allowed by the fact that some initial conditions of the study were not holding anymore (e.g. GPs were initially expected to recruit patients on an individual basis as they presented, but this was later substituted with automated searches in practice records). Subjects recruited in a PLR fashion confer greater power to the study. For more information on this amendment, see:

G:\EMS\CPTU\BEST3\Section 6 APPROVALS AND AUTHORISATIONS\8. Amendments\Amendment 6

Figure 1. Trial flowchart

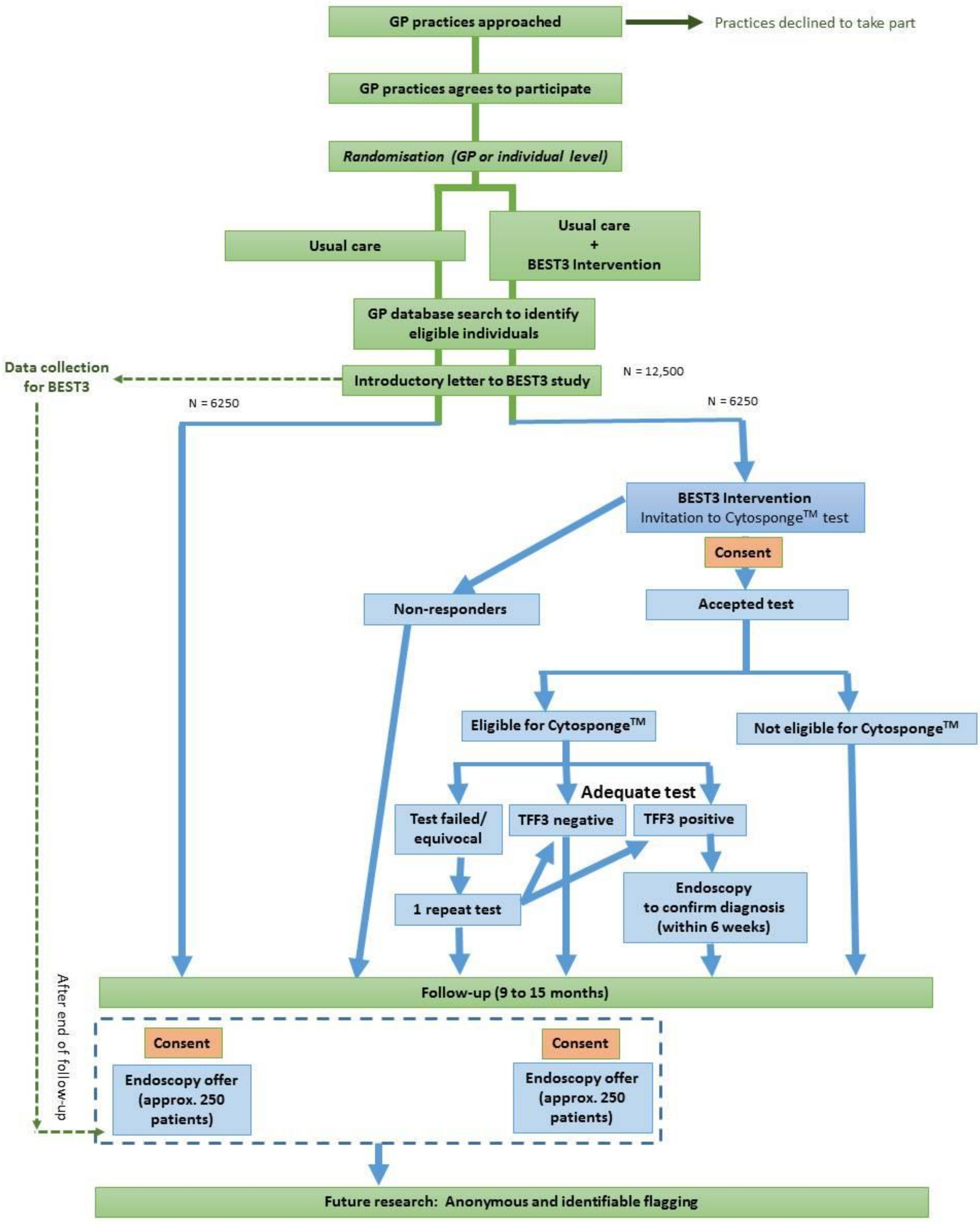

2.1 Primary endpoint

The main aim of the trial is to compare the numbers of histologically **confirmed BE diagnoses** between the two study arms. This will confirm whether the Cytosponge™ detects more BE than the current practice (i.e. GP referring a patient to endoscopy).

2.2 Secondary endpoints

To be assessed using data from the intervention arm *only*:

1) Diagnostic accuracy of Cytosponge™
2) Performance of Cytosponge™ in detecting severity of BE
3) Performance of Cytosponge™ in detecting intestinal metaplasia (IM) of the gastric cardia: based on IM detection by endoscopy of Cytosponge™-positive patients without BE
4) Performance of Cytosponge™ in detecting BE or IM of the gastric cardia
5) Performance of Cytosponge™ in detecting oesophageal adenocarcinoma (EAC) and gastric cancer
6) Sampling adequacy
7) Endoscopy referral rates for adequate test results and successful Cytosponge™ swallows
8) Patient acceptability of Cytosponge™: willingness to have the test and to have a repeat procedure, number of patients who fail to swallow, cancer worry, long-term emotional or physical harm, perceived risk of EAC, test experience.
9) Physician/nurse acceptability of Cytosponge™
10) Safety of Cytosponge™
11) Performance of repeat Cytosponge™ test

To be assessed using data from the usual care or *both* study arms:

12) Number of BE diagnoses missed in the current management
13) Number of undiagnosed BE in the general population vs in the group who received Cytosponge™
14) Prevalence and incidence of benign oesophageal conditions (BOCs)
15) Acceptability of endoscopy
16) Number of BE diagnoses for patients with a negative Cytosponge™ result
17) Quality control of endoscopic and pathology results

Epidemiological and longer-term objectives (for up to 10 years):

18) Prevalence and incidence of BE during the study
19) Prevalence and incidence of BE with dysplasia during the study
20) Prevalence and incidence of EAC during the study
21) Prevalence and incidence of IM and related gastric cancers during the study
22) Prevalence and incidence of BE up to 10 years after the study
23) Prevalence and incidence of BE with dysplasia up to 10 years after the study

24) Prevalence and incidence of EAC up to 10 years after the study
25) Prevalence and incidence of IM and related gastric cancers up to 10 years after the study
26) Research and development: Genetic and biochemical risk factors for disease progression (germline and somatic variants and other biomarkers) including targeted, exome level and whole genome sequencing

Health economics analysis:

27) To undertake modelling to predict the reduction in EAC-related mortality from this strategy (short and long term)
28) Cost of the Cytosponge™ test versus usual care
29) Cost-effectiveness of the Cytosponge™ versus usual care

2.3 Assessment of objectives

Assessment of primary endpoint

To assess the primary endpoint, research endoscopy findings (after the end of the follow-up period) will not be taken into account.

Because of the nature of the study, only participants who accepted the invitation to the Cytosponge™ test gave consent to the use of their data for study purposes. In order to collect primary endpoint data in consented Cytosponge™ patients, non-responders of the invitation and usual care participants, at least one of the following different data sources will be used:

1) ***Automated coded search*** for BEs, OGDs, EACs or upper gastrointestinal (GI) referrals: carried out on the GP electronic clinical records for all participants in all sites.
2) ***Manual case-note review*** of the GP clinical record for all participants identified by the coded search plus a number of other participants depending on the capacity of the sites: some practices will include all participants, some will include no additional participants and others will perform a manual review on a sample of participants chosen at random by the Trial statistician.
3) ***National Health Service (NHS) number linkage*** with hospital records for a limited number of sites, depending on the availability of relevant hospital information systems that routinely capture relevant diagnoses and geographical proximity of site in relation to its linked hospital.
Note: NHS number linkage will not be performed for all hospitals. For instance, in London, this step may be forgone because of the high population density and the consequent difficulty in determining the hospital catchment of sites.

Automated coded searches will be run by the study sites. The central study team does not provide the practices with the codes as different codes are in use in different practices. Following this step, the sites will fill in a spreadsheet prepared by the Trial team asking for the following information by sex and 10-year age range (50-59, 60-69, 70-79, 80-89, 90+): type of upper GI referrals and investigations; upper GI diagnoses during follow-up; treatment for BE. PLR sites will be asked to fill in two different spreadsheets, one for their usual care and one for their intervention patients. They will also indicate in the online database how many endoscopy referrals, BEs and adenocarcinomas (and the affected patients) they will have found in their records.

Manual case-note reviews consist of checking a patient's record closely and filling in an electronic database form with information on: length of follow-up, sex, age range, body mass index (BMI) range, smoking history, drinking history, GP consultations, type and length of acid-suppressant medications, Helicobacter pylori course, aspirin course, medication review, symptoms, any diagnosis following Cytosponge™ test, any treatment for BE.

For NHS linkage, the encrypted NHS numbers of all participants will be sent by practices and NHS numbers of all new diagnoses of BE will be sent by participating endoscopy units to Cambridge University (the "Trusted Third Party") for anonymous matching. NHS numbers will be scrambled using a one-way hashing system (to a SHA256 standard), so will be not identifiable to anyone outside the practice. Sites (GP practices and endoscopy units) will receive an Excel macro tool to perform the encryption. Any endoscopic diagnoses found through this method will be checked directly with the study sites. This step has received approvals from the NHS Research Ethics Committee as part of Amendment 6 to the BEST3 Protocol.

Where there is evidence of endoscopy from one of these sources, further details may be sought from clinical records. Study-related endoscopies for participants with positive or other relevant Cytosponge™ results will be used for this purpose.

Note: it is important to record the date up to which the coded search is looking for BE and OGDs/EACs/upper GI referrals. This date should be used as the date of last follow-up for that patient, i.e. any BE diagnosed after that date should not be included in the primary endpoint.

Table 1: A schematic of how Methods 1 and 2 work together.

		**SECOND STEP: manual case-note review returns…**		
		**BE diagnosis**	**Negative**	**Not done**
**FIRST STEP: automated coded search returns…**	**BE diagnosis**	For all patients with a BE diagnosis picked up in the coded search	N/A	N/A
	**OGDs/EAC/referral**	Some of the patients with an OGD/EAC/referral will have a BE diagnosis when their record is reviewed.	Some of the patients with an OGD/EAC/referral will not have any BE diagnoses when their record is reviewed.	N/A
	**Negative**	A randomly selected number of patients who have not been identified by the coded search will have their records reviewed, which will show a BE diagnosis.	A randomly selected number of patients who have not been identified by the coded search will have their records reviewed and no BE diagnoses will be found.	Most patients not identified by the coded search will not have their records reviewed manually.

The primary endpoint will be histological confirmed BE (column "BE diagnosis" in Table 1 plus any BEs picked up by the NHS number linkage exercise) only. (The case-note review should make clear where the BE was confirmed on histology or whether it was just the impression on endoscopic that was not confirmed.) Patients with BE identified via any of the three routes (automated search plus manual confirmation, manual search, record linkage) will be considered to have BE. Patients whose records have not been searched by any of the three approaches will be considered to have missing BE status. All other patients will be considered not to have a BE diagnosis. All patients with a BE/OGD/EAC/upper GI referral should have a manual review to confirm the diagnosis; if they have not had a manual review, their BE status will be considered as (partially) missing and handled through multiple imputations (of the outcome of the missing review).

Approximate balance in the number of full case-note reviews performed at random should be ensured between the two arms for practices in the CLR group.

Important! BEs known to the study team but not identified through at least one of the three methods describes above will not count towards the primary endpoint analysis. This is most likely to apply to cases detected following a positive Cytosponge™-TFF3 stain and resulting in an endoscopy performed by a study gastroenterologist. This rule is to ensure that the same data collection methods are used in the two study arms (as trial endoscopies are not available in the usual care arm) and helps avoid any biases. However, as a sensitivity analysis, all BEs known to the study team (prior to the exit research endoscopies) will be included. . For statistics relating only to the Cytosponge™ (e.g. PPV of the TFF3 stain), all cases of BE will be included.

Assessment of secondary endpoints

To be assessed using data from the intervention arm *only* (include all BE regardless of the route of study ascertainment):

1) Diagnostic accuracy of Cytosponge™ according to endoscopic findings only (focus: length of BE)
    - Positive Predictive Value (PPV) = proportion of patients with positive Cytosponge™ test result who have a confirmed BE diagnosis following endoscopy
    - PPV by length of BE detected (length of BE categories: >= C1, >= C2, >= C3)
    - Negative Predictive Value (NPV) = proportion of patients with negative Cytosponge™ test result receiving a research endoscopy after the end of the follow-up period who have a confirmed diagnosis of no BE
2) Performance of Cytosponge™ in detecting severity of BE, i.e. diagnostic accuracy of the test according to endoscopic and pathology findings
    - PPV of the test by severity of BE (except for score = 0)

    Severity of BE for positive Cytosponge™ patients will be scored after biopsy according to the following table:

Score	BE severity
0	Pathology report not available

1	IM of oesophagus on biopsy and endoscopic findings not seen in categories below
2	C1 or C0 up to M3 + IM
3	C2 or more, C0 M4 or more + IM
4	C3 or more
5	Low grade dysplasia (LGD)
6	High grade dysplasia (HGD) or T1a cancer

3) Performance of Cytosponge™ in detecting IM of the gastric cardia:
   - PPV of the test for detection of IM of the gastric cardia in patients without BE, i.e. BE with IM will be excluded
4) Performance of Cytosponge™ in detecting BE or IM of the gastric cardia:
   - PPV of the test for detection of BE or IM of the gastric cardia, i.e. patients from endpoints 1) and 3) combined
5) Performance of Cytosponge™ in detecting EAC and gastric cancer:
   - PPV of the test for detecting EAC or gastric cancer
6) Sampling adequacy (for first test, and first and repeat test combined):

   An *adequate* result is defined as: high-confidence negative (squamous and glandular cells), low-confidence positive (squamous and glandular cells with IM), or high-confidence positive (squamous and glandular cells with IM or cellular atypia).
   An *inadequate* result is defined as: processing/technical failure, low-confidence negative (squamous cells only), or equivocal (squamous and glandular cells with equivocal TFF3 staining).
   Sampling adequacy will be reported for the following measures for first test, and then for first and repeat test combined (i.e. not considering the result of the first test for those patients having a repeat test):

   - Inadequacy rate = proportion of Cytosponge™ test results that are deemed insufficient/inadequate due to processing or technical failure, low-confidence negative (squamous cells only) or equivocal (squamous and glandular cells with equivocal TFF3 staining), to be reported with number of.
   - Number and proportion of Cytosponge™ test results deemed insufficient/inadequate due to technical failure only
   - Number and proportion of Cytosponge™ test results deemed low-confidence negative only
   - Number and proportion of Cytosponge™ test results deemed equivocal only

   The above measures will also be presented for first test only and for first and repeat test combined, excluding those patients receiving an inadequate test result but not attending their repeat test.

7) Endoscopy referral rates for ‘adequate’ test results and successful Cytosponge™ swallows:

- Proportion of positive Cytosponge™ patients out of all the ones with an adequate test result, both for first test only, and for first and repeat test combined
- Proportion of positive Cytosponge™ patients out of all patients swallowing successfully a Cytosponge™ test, both for first test only, and for first and repeat test combined

8) Patient acceptability of Cytosponge™:

At first test:

- Number/proportion of patients invited who show interest
- Number/proportion of patients invited who receive screening phone call and how many of these are not eligible to have the test
- Willingness to have the test: number/proportion of patients invited who attend appointment
- Number/proportion of patients who fail to swallow
- Number of attempts to swallow per patient

At repeat test (in case of an ‘inadequate’ result at first test):

- Number/proportion of patients with an ‘inadequate’ first test result invited to a repeat test
- Number/proportion of patients with an ‘inadequate’ first test result interested or not interested in a repeat test
- Willingness to have repeat procedure: number/proportion of patients with an ‘inadequate’ test result who attend a second appointment
- Number/proportion of patients who fail to swallow
- Number of attempts to swallow per patient

All of these figures for first and repeat tests will be presented in a flowchart similar to the one used for the open Data Monitoring Committee (DMC) reports (see Section 10 for reference).

At baseline (before first test):

- Cancer worry and long-term emotional or physical harm as measured by STAI-6, a short-form of the state scale of the Spielberger State-Trait Anxiety Inventory
- Perceived risk of developing EAC: overall and compared to a person of the same age

At day 7-14:

- Cancer worry and long-term emotional or physical harm as measured by STAI-6
- Perceived risk of EAC: overall and compared to a person of the same age
- Test experience as measured by visual analogue scale (0 = “Completely unacceptable”, 10 = “Completely acceptable”)

- Test experience as measured by the Inventory To Assess Patient Satisfaction (5-point ordinal scale with 18 items)
- Difference in STAI-6 scores at day 7-14 and baseline

9) Physician/nurse acceptability of Cytosponge™: assessed through qualitative interviews (not discussed in this document).

10) Safety of Cytosponge™:

    See Section 8.

11) Performance of repeat Cytosponge™ test:

    - Rate of conversion to an ‘adequate’ result after an ‘inadequate’ first test result = proportion of repeat tests that have an ’adequate’ result, to report with number.
    - Number/proportion of repeat tests that have an ‘inadequate’ result.
    - Chances of a repeat test result being TFF3 positive (high or low-confidence) after a low-confidence negative result at the first test.

    The measures above will be recalculated at a second stage excluding any patients invited to a repeat test and refusing to attend.

<u>To be assessed using data from the usual care or *both* study arms:</u>

12) Number of BE diagnoses missed in current management:

    - Usual care arm: research endoscopy findings

13) Number of undiagnosed BEs in the general population vs in the group who received Cytosponge™:

    - Intervention arm: research endoscopy findings
    - Usual care arm: research endoscopy findings

14) Acceptability of endoscopy:

    - Intervention arm: number attending Cytosponge™ appointment
    - Usual care arm: number attending research endoscopies

    Proportion of participants in the usual care arm who attend their research endoscopy invitation compared to proportion of participants in the intervention arm who attend their Cytosponge™ invitation.

15) Number of BE diagnoses for patients with a negative Cytosponge™ result:

    - Intervention arm: research endoscopy findings

    Number of false negatives of the test and false omission rate.

16) Quality control of endoscopic and pathology results:

    A central review by the study team of all endoscopic and pathology records of positive Cytosponge™ patients undergoing a confirmatory endoscopy will be performed. Its results will be compared to the endoscopic and pathology results of the study sites by quantifying the number of BE diagnoses missed, the number of any other malignant diagnoses missed and, if relevant, the number of BEs falsely detected. “True” PPVs will be calculated for overall results (first and repeat test combined).

A similar review will look into the results of research endoscopies for usual care and negative Cytosponge™ patients.

Epidemiological and longer-term objectives (for up to 10 years):

To be detailed in the BEST3 Epidemiological Analysis Plan.

Health economics analysis:

This is detailed in the BEST3 Health Economics Analysis Plan and will not be discussed here.

2.4 Level of significance

The level of significance that will be used in all the statistical analyses is 5%, two-sided. However, 95% confidence intervals will be preferred to *p*-values in the final report.

2.5 Sample size

This section combines the initial power calculations with the ones amended after the Milestone 1 review in January 2018, when the study design was changed from CLR only to CLR and PLR. A more detailed explanation on sample size calculations and variable follow-up periods is available here:

G:\EMS\CPTU\BEST3\Section 26 STATISTICS\26.3 Power calculations\Sample size (following Amendment 6)\BEST3_Sample size.pdf

2.5.1 Changes to study design after Milestone 1 review

The BEST3 study was planned as a cluster randomised trial stratified by practice size in order to achieve a reduction in the variance of the estimated treatment effect. This is done by reducing the coefficient of variation of the cluster size within strata. This gives a higher power to the study.

In the initial stage of the study, uptake of the Cytosponge™ was anticipated to be 50%. A lower uptake than anticipated was the main reason why a change in study design was needed – the sample size for a CLR trial with uptake lower than 50% would have been too large to be sustainable for the study. Given the substantial impact to the power of the trial, it was proposed that an additional PLR randomisation design be added to the trial. The reason for the lower than anticipated participation and for why we considered it important to adapt the design to take account of it is explained below.

Originally, the trial was envisaged as recruiting patients presenting to their GPs with (incident) symptoms of reflux. For this reason, we: (1) felt that GP-practice-level randomisation was essential; and (2) assumed that with the personal endorsement of the GP and the fact that patients were essentially asking their GP for help with their reflux, acceptance of the offer of a Cytosponge™ test would be high (50%).

For practical reasons (related to trial delivery), BEST3 has been randomising “prevalent” patients, i.e. practices identify patients who have drug prescription records indicating reflux and invite them (or not, depending on randomisation) all at once. Identification using prescribing history rather than at the presenting appointment has resulted in a much lower uptake than anticipated (approximately 27%). (Patients may have been on reflux medication for many years without recently having had symptoms or consulted with their GP.) We still believe that, were the Cytosponge™ to be offered by GPs to patients presenting with reflux as routine practice, the uptake would be much higher.

To obtain sufficient power without greatly expanding the number of participants and practices required, we adapted the design to allow PLR. Practices already set up or trained to take part in the CLR continued with this randomisation method. Practices engaged at a later date would use individual randomisation.

2.5.2 Sample size calculations

The sample size calculation is based on the following assumptions:

- p_{BE}: BE prevalence in individuals eligible for the study is 4%
- p_E: 10% of patients in the usual care arm will be referred to endoscopy for clinical reasons (after excluding urgent referral)
- $p_{BE|E}$: The prevalence of BE in patients referred to endoscopy in the usual care arm is 6%
- s_C: Cytosponge™ -TFF3 sensitivity is 85%
- s_E: Endoscopy sensitivity is 100%
- u: uptake of the Cytosponge™ test is expected to be 27%

Since only 27% of patients in the Cytosponge™ arm are predicted to have the Cytosponge™ test and patients who do not take up the offer of the test will have the same management as if they were in the usual care arm, we only expect 1.38% of patients in the **intervention arm** to be diagnosed with BE:

$$\%\text{ BEs in intervention} = u[p_{BE}s_C + (1 - s_C)p_E p_{BE|E}] + (1 - u)p_E p_{BE|E}s_E$$

Note that:

- $up_{BE}s_C$ is the proportion of patients who get a positive Cytosponge^{TM}test result and have BE
- $(1 - u)p_E p_{BE|E}s_E$ is the proportion of patients who do not take up the Cytosponge^{TM}invitation and who are diagnosed with BE after receiving an endoscopy
- $u(1-s_C)p_E p_{BE|E}$ is the proportion of patients who get a negative Cytosponge^{TM}test result despite the fact that they have BE and who then move on to have BE diagnosed by endoscopy

$$\begin{aligned}\%\text{ BEs in intervention} &= 0.27[0.04 \cdot 0.85 + (1 - 0.85)0.1 \cdot 0.06] + (1 - 0.27)0.1 \cdot 0.06 \cdot 1 \\ &= 1.38\%.\end{aligned}$$

Furthermore, we expect 0.6% of patients in the **usual care arm** to be diagnosed with BE:

$$\%\text{ BEs in usual care} = p_E p_{BE|E}s_E = 0.1 \cdot 0.06 \cdot 1 = 0.6\%.$$

For a 90% power in an individually randomised trial) comparing 0.6% with 1.38%, we would need 6764 individuals (3382 in each arm). In Stata, this is given by the following code:

```
power twoproportions 0.0138 0.006, power(0.9)
```

In a CLR trial, individuals within the same cluster do not act independently, so it is necessary to randomise more individuals. We can calculate how many people one needs to include in a CLR trial to provide the same amount of information as one person in a PLR trial. That number is called the variance inflation factor (VIF). With a VIF of 3.0, one would need 9000 patients in a CLR trial to have the same power as

3000 in an individually randomised trial. As we increase the number of invitees per cluster (i.e. GP practice), the VIF increases, too. In simple terms, there are diminishing returns.

The sample size calculated above would have initially been multiplied by the **variance inflation factor** (VIF). We estimated the VIF as follows:

$$VIF = 1 + \left(\left(\frac{k-1}{k}CV^2 + 1\right) mean - 1\right) ICC$$

where:

- k, the number of practices in the group
- $mean$, the average size of the practices in the group
- CV^2, the square of the coefficient of variation of the number of patients per practice, which was in turn calculated by dividing the standard deviation by the mean
- ICC, the intra-class correlation coefficient

The **intra-class correlation** (of the proportion of patients with BE) was assumed to be 0.025.

The VIF can be calculated overall or by stratum. After the Milestone 1 review, practices in the cluster-randomised group where divided into two groups: practices whose enrolment and size (i.e. recruitment numbers) was confirmed, and practices whose enrolment was expected and whose size was estimated. For both groups, practices were grouped into strata based on their size: 50-65, 66-90, 91-125, 126-175, 176-225. With data available on 9th January, we estimated the VIF in each stratum to be: 2.44, 2.96, 3.59, 4.77, and 5.91, respectively, for the confirmed practices. We also estimated VIFs for the projected practices and anticipated 11,816 patients in total from 100 practices contributing the equivalent of 2924 individually randomised patients (1724 "confirmed", 1200projected).

The required **sample size** in a PLR setting was calculated above to be **6764**. The equivalent confirmed and projected sizes are to be subtracted to this number, and the result is the number of patients that needed to be recruited in the **PLR group**:

6764 – 1724 – 1200 = **3840**

and the total sample size overall was 15,656 (11,816 + 3,840) participants.

During the trial, recruitment numbers were checked several times and the size of the PLR group was adjusted accordingly.

As of 13th June 2019, **7844** participants were recruited in the **CLR group** (equivalent size: 2120) and **5390** in the **PLR group** after the initial 14-day opt-out period, for a total of 13,234 participants. Note that the CLR practices eventually recruited fewer patients than expected at the time of the Milestone 1 review, and that implied a higher recruitment in the PLR group, whose participants contribute more to the power of the study.

The table below shows a range of possible values for our sample size depending on how we adjust the estimate of our parameters.

	BE prevalence	Cytosponge™ TFF3 sensitivity	Endoscopy in usual care arm (over 12 months)	BE prevalence in patients getting routine endoscopy	Uptake of Cytosponge™	Intra-class correlation coefficient	Sample size if only individual randomisation	Equivalent sample size accounted for by cluster randomisation (11,816 patients in 100 practices)	*Remaining sample size for PLR group*
Our assumptions	4.0%	85%	10%	6%	27%	0.025	6764	2924	***3840***
	3.9%	85%	10%	6%	27%	0.025	7098	2924	***4174***
	4.1%	85%	10%	6%	27%	0.025	6456	2924	***3532***
	4.0%	***84%***	10%	6%	27%	0.025	6894	2924	***3970***
	4.0%	***86%***	10%	6%	27%	0.025	6638	2924	***3714***
	4.0%	85%	***9%***	6%	27%	0.025	5658	2924	***2734***
Varying assumptions	4.0%	85%	***11%***	6%	27%	0.025	8176	2924	***5252***
	4.0%	85%	10%	***5%***	27%	0.025	5048	2924	***2124***
	4.0%	85%	10%	***7%***	27%	0.025	9344	2924	***6420***
	4.0%	85%	10%	6%	***26%***	0.025	7190	2924	***4266***
	4.0%	85%	10%	6%	***28%***	0.025	6380	2924	***3456***
	4.0%	85%	10%	6%	27%	***0.024***	6764	**3012**	***3752***
	4.0%	85%	10%	6%	27%	***0.026***	6764	**2840**	***3924***

2.5.3 Lower uptake of the Cytosponge™ invitation

In the updated sample size calculations of Milestone 1, uptake of the Cytosponge™ invitation was expected to be 27%. However, in the data available in June 2019, uptake was closer to 24.0%, which would be equivalent to 84% power according to the same assumptions as in the section above. At the same time, it should be noted that currently we are expecting to recruit 746 patients more than needed by our sample size calculations, so that would raise the power of the study to 87%.

The following table shows how the power of the study varies by keeping the same sample size and changing some of the initial assumptions.

Uptake	Prevalence of BE in eligible population	Prevalence of BE in those referred to endoscopy	Sample size (with 90% power)	Power with n = 6764
27.0%	0.04	0.06	6768	90%
24.0%	0.04	0.06	8260	83%
24.0%	0.05	0.06	5388	95%
24.0%	0.04	0.05	10126	75%
24.0%	0.05	0.05	6246	92%
23.0%	0.04	0.06	8886	81%
22.0%	0.04	0.06	9350	79%
21.0%	0.04	0.06	10126	75%

2.5.4 Variable follow-up periods

Because of delays in the implementation of the change of study design (from CLR to CLR with additional PLR group), it was decided that participants/practices could have

a follow-up longer than 12 months to compensate for participants/practices who will have a follow-up shorter than 12 months due to time constraints.

Different practices will have different follow-ups as long as the **weighted average follow-up** for the study will be greater than 12 months, as shown by the following formula:

$$\text{avg}_{\text{FU}} = \frac{\sum_{i=1}^{P} F_i M_i + \sum_{j=1}^{Q} F_j N_j}{\sum_{i=1}^{P} M_i + \sum_{j=1}^{Q} N_j} \geq 12$$

where F_i, F_j are the different lengths of follow-up (in months) for each different group, M_i are the numbers of patients with different follow-ups in the CLR group in 'equivalent size' terms, N_j are the numbers of patients with different follow-ups in the PLR group, $\sum_{i=1}^{P} M_i$ is the total size (in 'equivalent' terms) of the CLR group, and $\sum_{j=1}^{Q} N_j$ is the total size of the PLR group.

According to our current projections, all practices will have between 8 and 18 months of follow-up. The end date of follow-up will be when a site performs their local coded search; it will therefore be taken from the Coded Search case report form (CRF).

Note: in case a weighted average follow-up of 12 months or more could not be guaranteed, the power of the study would be less than 90%. The following table illustrates the potential loss in power with shorter follow-up:

Total average follow-up (months)	Factor by which to inflate sample size
9	1.24
9.5	1.18
10	1.13
10.5	1.09
11	1.06
11.5	1.03

A detailed explanation on how to calculate the factors by which to inflate the sample size is available here:

G:\EMS\CPTU\BEST3\Section 26 STATISTICS\26.3 Power calculations\Sample size (following Amendment 6)\BEST3_Sample size.pdf

2.5.5 Randomisation algorithm

For the CLR group, practices were randomised to either the intervention or the usual care arm. This was done using a randomisation algorithm that stratified by practice size.

For the PLR group, a single randomisation list was created using block randomisation, allowing for 40 practices of 250 patients each to be randomised, for a total of slightly more than 10,000 potential patients (depending on the size of the last randomisation block). A further step of randomisation was introduced to decide the order in which the practices would be assigned to the list. In January 2009, in order to allow for more practices to be enrolled into the study, a new randomisation list with 10 more practices of 250 patients each was created.

More details on the randomisation algorithms and validations can be found here:

G:\EMS\CPTU\BEST3\Section 9 REGISTRATION AND RANDOMISATION

3. General analysis definitions

3.1 Study periods

Follow-up periods will be **variable** as shown in Section 2.5.4.

3.2 Study populations

3.2.1 Intention-to-treat population

The intention-to-treat (ITT) population will include all participants enrolled into the study. The following participants will be *included* in the ITT analysis:

- Deceased patients:
  BE status at death will be used as their BE status at the end of the trial.
- Study subjects that moved away from a practice:
  We are unlikely to know how long such patients were followed because we only receive aggregate data regarding most patients. For this reason, we will treat them as if they were followed for the same duration as other patients in that practice unless we know when they moved, in which case their follow-up will be treated as censored at that time.
- Participants lost to follow-up due to local information governance restrictions:
  If we have no follow-up on all patients in a particular practice, that practice will effectively be excluded. The fact that this has happened will be noted. Individuals with a Cytosponge™ test will still be included in the intervention-arm only results assuming that BE found as a result of a positive Cytosponge™ will have been recorded. If a patient withdraws consent for their data to be used for research purposes (‘Type 2’ objection), they will be excluded.

As detailed data are only available for consented patients in the intervention arm, for all patients we assume a follow-up equal to the follow-up of their practice, with the exception of any known participants invited to a research endoscopy before the end of their follow-up period. However, it should be noted that IDs of participants with a BE/OGD/EAC/upper GI referral will be collected for both arms and available for the statistical analysis.

Subjects who opted out (Type 2 objection to their data being used, or explicitly writing to the study team asking for their data to be removed from the database) *after* being entered into the study (i.e. more than 14 days after receiving the trial introductory letter) will be *excluded* from the ITT analysis and any further analyses. The numbers of such patients in each arm will be reported.

Further exploratory epidemiological analyses to study the risk of BE according to baseline/demographics data may be detailed in a separate SAP for epidemiological analyses.

3.2.2 Per-protocol population

The per-protocol analysis will exclude any patient who we know should not have been randomised had the inclusion and exclusion criteria been followed precisely (for example, a patient outside of the age range). It may not be possible to identify individuals who do not meet the eligibility criteria in terms of acid-suppression use (see below).

The per-protocol analysis will also exclude any study subjects in the PLR group randomised to one study arm but erroneously assigned to the other and any participants who moved away before receiving the invitation to the test.

The per-protocol analysis will be performed for the primary endpoint only.

Using the number of case-note reviews available in a practice as the denominator, we will calculate the proportion of patients in a practice that have less than 6 months' worth of acid-suppressant medication prescriptions in the year preceding baseline. This proportion will be used as a threshold in our sensitivity analyses. However, practices recruited at the late stages of the Trial may not perform any additional case-note reviews other than for patients picked up by the local coded search of their clinical information system due to time restrictions. In order to overcome these issues, we plan to perform three different sensitivity analyses:

Sensitivity analysis 1: Exclude the practices we know have more than 20% of patients with less than 6 months' worth of acid-suppressant medication prescriptions as well as those practices for which this information is unknown i.e. there has been fewer than 20 patients whose notes have been reviewed manually.

Sensitivity analysis 2: Exclude only the practices we know have more than 20% of patients with less than 6 months' worth of acid-suppressant medication prescriptions.

Sensitivity analysis 3: Only exclude individual patients known to have had less than 6 months' worth of acid-suppressant medication prescriptions at randomisation.

Sensitivity analyses will be performed for the primary endpoint only.

Further investigations on this will compare the proportion of BEs detected in patients with less than 6 months' worth of acid-suppressant medication prescriptions vs the proportion of BEs detected in patients with more than 6 months' worth of such prescriptions. Because of the nature of the data (aggregated data + individual-level data for consented Cytosponge™ patients and patients who have their case-note reviewed), this exercise will only be performed with the individual-level data available.

3.2.3 Non-compliance corrected (ITT) population

An adjustment for lack of Cytosponge™ use in the intervention arm (non-compliance) will be made following the method detailed in Cuzick et al., which gives an estimate of the effect of the intervention for those who attend the Cytosponge™ test invitation. This will show the causal impact of the Cytosponge™ on BE detection.

BE detection in compliers in the intervention arm will be compared to BE detection in potential compliers in the usual care, i.e. participants who would have received the test if they had been offered it. In order to do that, the proportions of actual or potential compliers/non-compliers in the two arms is assumed to be the same, and so is the proportion of BE detection in actual or potential non-compliers. The proportion of BE detection in potential compliers in the usual care arm will be estimated as:

$$\frac{no.BEs\ in\ usual\ care - no.BEs\ in\ potential\ non\ compliers}{no.potential\ compliers}$$

Data on number of BEs detected in the two study arms will come from the ITT analysis on the primary endpoint.

The relative protection given by the intervention in those who comply is to be estimated as

$$\frac{S_{11}/N_1}{S_0/N_0 - S_{10}/N_1}$$

where $\{S_0, S_{10}, S_{11}\}$ and $\{N_0, N_{10}, N_{11}\}$ are the numbers of BE detected and the numbers of individuals in the usual care arm, in the non-complier population (intervention) and in the complier population (intervention), respectively.

Note: in order to obtain an estimate of the non-compliance corrected effect, data from the CLR group will be treated in the same way as data from the PLR group, i.e. the two datasets will be merged and the effect of clustering in the CLR group will not be considered. However, in order to obtain confidence intervals for the effect, the variance inflation factor for the study as a whole will be applied to the variance of the effect that ignores the clustering. It is noted that this method assumes that the compliance within cluster is independent of the BE prevalence within cluster. New methodology to better adjust for the clustering may be developed.

3.2.4 Safety population

The safety analysis will look at all participants attempting to swallow a Cytosponge™: both the ones producing a Cytosponge™ sample ('successful swallows'), independently from the test result, and the ones not able to swallow a sponge. The endpoints related to the safety of the Cytosponge™, i.e. total number of AEs, number of AEs per participant, number/proportion of participants experiencing an AE, will be analysed on this population.

3.3 Subgroup definitions

Primary endpoint analysis will be carried out separately for the CLR and PLR groups as asked by the Medicines and Healthcare products Regulatory Agency (MHRA) in a communication on 31 May 2018. If the results from both subgroups favour the study intervention, i.e. the proportion of participants with diagnosed BE is greater (regardless of the level of significance) in the invitation to the Cytosponge™ arm than in the usual-care arm, then a combined analysis shall be performed.

3.4 Treatment assignment and treatment groups

Participants in the study were selected by an automated search in GP databases followed by a manual review of their records. All study subjects received an introductory letter to the study, allowing them 14 days to opt out of anonymised data collection. Following this, GP practices (for the CLR group) or participants (for the PLR group) were randomised to either receiving an invitation to the Cytosponge™ test or to usual care.

Non-responders of invitation to the Cytosponge™ were managed as were the patients in the usual care arm.

Participants with a positive Cytosponge™ result were invited to a **confirmatory endoscopy**. Negative Cytosponge™ patients were subsequently managed as were patients in the usual care arm.

Note: A small sample of patients in the usual care arm and negative Cytosponge™ patients were invited to a **research endoscopy** *after* the end of their follow-up period. The result of their research endoscopy is *not* part of the primary endpoint analysis. A handful of patients invited to the test were also invited to have a research

endoscopy at the beginning of the roll-out of the procedure, but invitations to that group were stopped shortly thereafter, and any research endoscopy results in this group of patients will not be taken into account for any analyses.

4. Patient disposition

4.1 Compliance to the Cytosponge™-TFF3 test

Compliance to test (intervention arm) will be defined on two aspects: attendance and successful swallows.

Participants will be provided with two opportunities to successfully swallow the device. A participant will be considered as having had the Cytosponge™ test if he or she has at least one successful swallow. Attenders will include patients who successfully swallow a sponge and those who present at their appointment but are not able to produce a successful swallow.

Study subjects successfully swallowing a Cytosponge™ may produce a sample deemed inadequate because of processing/technical failures or because the test result is considered to be low-confidence negative (squamous cells only) or equivocal (squamous and glandular cells with equivocal TFF3 staining). These patients will be invited to a repeat Cytosponge™ test.

The following numbers and proportions on compliance will feed into the Trial flowchart (see Figure 2):

- responders: overall, to first invitation letter only, to second invitation letter only
- interested and not interested (out of all responders)
- received screening phone call (out of interested): eligible, ineligible
- attenders and non-attenders/withdrawn (out of eligible)
- produced a successful swallow and unable to swallow (out of attenders)
- ‘inadequate’ samples (out of successful swallows) at first attempt and at repeat test: processing/technical failures, low-confidence negative (squamous cells only), equivocal (squamous and glandular cells with equivocal TFF3 staining)
- participants with ‘inadequate’ samples invited for a repeat test, attending the test, producing a successful swallow or unable to swallow
- ‘adequate’ samples (out of successful swallows) at first attempt, at repeat test, overall (i.e. only repeat test counts for participants having two tests) and in total (i.e. all tests counts): negative (squamous and glandular cells), low-confidence positive (squamous and glandular cells with IM), high-confidence positive (squamous and glandular cells with IM and cellular atypia)

Time from first invitation letter to response will be analysed with a Kaplan-Meier estimate, where the event is “responding to the invite” and survival is “not responding to the invite”. However, it should be noted that, in a handful of cases when patients replied very late (> 1 month after invitation) to their test invite, the nurse was not able to offer an appointment (because the study was no longer working in the area) and the patient was marked as a non-responder.

4.2 Compliance to confirmatory endoscopies

Compliance to confirmatory endoscopies will be measured out of all patients receiving a low or high-confidence positive Cytosponge™ test result. We will report on number of attenders and types of diagnoses.

4.3 Compliance to research endoscopies (after end of follow-up)

Compliance to research endoscopies will be measured out of all patients receiving an invite to a research endoscopy. We will report on number of responders, interested/not interested, attenders and types of diagnoses both overall and by study arm.

Note: only participants in the usual care arm and patients receiving a negative Cytosponge™ test result were invited to a research endoscopy. A limited number of 'non-responders', i.e. patients who did not take up their Cytosponge™ invitation, were also invited to research endoscopies at the beginning of the rollout of this part of the trial; their invites were suspended shortly after. Despite the small figures, we will report on number of non-responders invited and attending a research endoscopy in the final statistical report.

5. Demographics and baseline characteristics

The following data will be available at baseline for each GP practice:

Usual care, intervention and PLR practices will send the study team the following baseline data in *aggregated* form (Excel spreadsheet see below):

- Number of participants enrolled by sex and age group
- Drugs administered and dosage

For PLR practices, the trial arm is not included in the aggregated baseline data.

Sex	Age bracket (yrs)					
	50-59	60-69	70-79	80-89	90-99	Total
Female<br>Male<br>Total						

Demographics and baseline characteristics will be presented by summary statistics. No statistical tests will be performed to compare these between study arms.

Number of sites, number of participants per study arm and average practice size will also be presented.

Patients who take up the Cytosponge™ invitation will have to complete the following CRFs, with the following information available to the Trial Statistician:

- Personal details CRF: sex, year of birth, ethnicity (sensitive data, available to the Statistician via the Trial Senior Research Application Programmer only)
- Allergies CRF
- Baseline Clinical CRF: height, weight, waist/hip circumference, medication for reflux symptoms and dose, gastro-oesophageal reflux disease impact scale questionnaire, heartburn start, H. Pylori, comorbidities
- Baseline Questionnaire CRF: education, smoking history, alcohol intake, risk perception, STAI 6 questionnaire, family history

A number of patients selected at random, both in the usual care and the intervention arm, will have their baseline data and any data regarding a potential upper GI diagnosis and treatment reviewed at the end of their follow-up period. When possible,

sites will review the records of all their patients and fill in a case-note review CRF for each one of them.

A copy of all CRFs is available here:

G:\EMS\CPTU\BEST3\Section 10 CASE REPORT FORM (CRF)\10.1 Current version\BEST3 eCRFs

5.1 Characteristics collected during the study

Participants taking up the Cytosponge™ invitation will also see the following information gathered on them:

- 7-14 day follow-up questionnaire CRF: questions on different elements of the test experience, perceived risk of oesophageal cancer, STAI 6

5.2 End-of-study data

A number of patients selected at random by the Trial Statistician, both in the usual care and the intervention arm, will have their demographics data collected at the end of their follow-up period. These will be the same patients randomly selected to have their baseline data reviewed and a case-note review CRF will be filled in for each one of them.

The primary endpoint data on BE diagnosis will be collected via local coded search + manual case-note review + NHS number linkage as explained in Section 2.3.

5.3 Prior medications and treatments

Acid suppression medication data at baseline is available in aggregated form for all practices. Only medication dose and drug name (not length of treatment) will be available for all individuals.

A number of patients selected at random, both in the usual care and the intervention arm, will have their medication data at baseline reviewed and their medication data at end of study collected at the end of their follow-up period. A case-note review CRF will be filled in for each one of them. For these patients, length of treatment will be available in three monthly categories up to one year and more than one year.

Aggregated data on medication will be compared to medication data gathered during case-note reviews in those practices performing a review of all of their patients.

6. Interim analysis and timing for analysis

6.1 Interim analysis

A Milestone 1 review was planned in January 2018 after six months of opening the first GP site to evaluate the proportion of eligible individuals per surgery (% of population covered), the proportion of participating individuals (% of eligible population), and the Cytosponge™ uptake. This eventually led to a review of the sample size and of the study design (from CLR to CLR and PLR). For more details on this, see Section 2.5.1.

Closed endpoint data on participants who took up the Cytosponge™ invitation were presented at the closed sessions of the DMC meetings of March 2018, October 2018 and October 2019. Reports for the closed session meetings are available here:

G:\EMS\CPTU\BEST3\Section 17 TRIAL COMMITTEES\Data Monitoring Committee (DMC)\Meetings (agenda and minutes)\Reports for closed session

6.2 Time-points for analysis

Only a statistical analysis at the end of the trial is planned.

The last patients were randomised into the trial on 05/04/2019. The coding for the statistical analysis will start in October 2019. The final data lock is expected to happen on 31 January 2020.

7. Efficacy analysis

The statistical analysis will be run using Stata and R. The trial statistician will write the code, which will then be checked by another statistician. The primary analysis of the primary endpoint will be undertaken independently by a second statistician. If the two analyses do not produce identical results, the two statisticians will review their analyses together to reach consensus.

7.1 Method for analysis of endpoints

7.1.1 Analysis of primary endpoint

The power under various assumptions regarding sensitivity, BE prevalence and intra-class correlation will be calculated based on the actual numbers recruited, the uptake observed and the actual duration of follow-up.

Null Hypothesis: The BE detection rate at 12 months (excluding any found on random exit endoscopies) is the same in the intervention arm and the control (usual care) arm.

Alternative hypothesis: The BE detection rate at 12 months is greater in the intervention arm than in the control (usual care) arm.

The CLR and PLR group will be first analysed separately; if the results from the two parts of the study favour the test, a combined analysis will be performed.

Primary endpoint data will be collected according to the methods explained in Section 2.3. This aims at guaranteeing an equal approach to data collection in the two study arms, but implies that, for the primary endpoint analysis, we will only consider BEs that were ascertained systematically through one of the three methods.

Sites have variable follow-ups. The number of person-years of follow-up will be calculated by taking as the end date the date of the local coded search in a practice and as start date the date the first letter of introduction to the study was sent plus 15 days. Follow-up will be considered until whichever is first: diagnosis of BE, the date of the systematic search for BE, the day before a research endoscopy. BE found on research endoscopy will not be counted towards the primary endpoint.

Rates will be calculated out of 1000 person-years. A single rate will be calculated during follow-up in the control arm. Two rates will be calculated in the intervention arm: the rate within four months of randomisation and the rate beyond four months from randomisation. In order to estimate the average rate within 12 months of randomisation in the intervention arm, a weighted average of these two rates will be taken with weights 2:1.

The methods mentioned in the sections below are taken from:

Hayes RJ and Moulton LH. (2009). *Cluster randomised trials*. ed. Boca Raton, FL: Chapman & Hall/CRC, pp. 178-9.

7.1.1.1 Unadjusted analysis

CLR group

The CLR group is stratified by cluster size, i.e. number of participants per practice, as per the categories defined in the Milestone 1 review of the sample size (see Section 2.5.2): 50-65, 66-90, 91-125, 126-175, 176-225.

As a primary analysis, we will run a regression analysis based on individual-level data, followed by a secondary analysis based on cluster-level summaries to ensure that the conclusions are robust.

We will report on number of clusters and patients by stratum and study arm, and on the weighted average follow-up for the CLR group as shown in Section 2.5.4.

Individual-level data

We will first report on cumulative BE detection rate at one year (/ 1000 person-years) by stratum and study arm, and overall, using individual-level data. The one-year rate in the intervention arm will be estimated assuming a constant rate in the first four months and a (possibly different) constant rate thereafter (up to 18 months).

Primary endpoint data will be analysed by a mixed-effects Poisson regression for BE with fixed effects for the treatment and random effects to account for between-cluster variation (i.e. a random effect for the level of BE in each GP practice), with the number of person-years of follow-up as the offset. Additional fixed effect parameters will be included to account for strata (size of clusters in each stratum: 50-65, 66-90, 91-125, 126-175, 176-225).

The resulting 12-month rate ratio will be reported with 95% confidence interval and will be formally tested to see if it is significantly greater than 1.0 (with a two-sided alpha of 0.05).

To fit the Poisson regression random effects model to data, we will model the random effects using a log-gamma distribution.

In Stata, the `mepoisson` command performs a mixed-effects Poisson regression. See Section 11.2.1.1 of Hayes and Moulton for an explanation on the method. There will be two observations (and two durations of follow-up) for each patient: one for the first four months and a second thereafter. There will be a separate treatment effect for each period. The overall treatment effect will be calculated as the weighted mean of the two treatment effects using the Stata command `nlcom`.

Cluster-level data

As a secondary analysis, we will also analyse the data from the cluster-randomised practices using cluster level data. The analysis on cluster-level summaries will follow closely the method explained in Section 12.3.2 of Hayes and Moulton (see also Example 12.3 in the same textbook for a coding example in Stata in the case where the number of clusters (i.e. GP practices) in each stratum is balanced across study arms).

We will first report on mean BE detection rate (/ 1000 person-years) by stratum and study arm, and overall, using cluster-level data. As before, the cumulative rate at 12 months for the intervention arm will be estimated by dividing the follow-up into two periods: the first four months, and the subsequent follow-up (up to a maximum of 18 months).

By stratum: The rate ratio of BE detection for each stratum (approximate number of eligible patients in the practice) will be calculated as the exponential of the difference of the mean log(rates) for BE detection in the two study arms, which is equivalent to the ratio of the geometric means of the rates in the two arms for that stratum. 95% confidence intervals for stratum-specific RRs are calculated according to the method in Section 10.3.2.2 and Example 10.5 of Hayes and Moulton, using the number of clusters minus 2 as the degrees of freedom for the *t* distribution, the number of clusters per study arm, BE detection rates and standard deviations of cluster rates by study arm.

Overall: The overall estimate of the log rate-ratio will then be calculated as a weighted average of the stratum-specific estimates, with weights depending on the number of clusters per study arm and under the assumption that the between-cluster variance in log-rates within each combination of stratum and study arm is constant.

A *stratified t-test* will allow us to test the null hypothesis that the true rate ratio is 1 and to calculate the 95% confidence interval for the RR. The between-cluster variance for this test will be calculated as the residual mean square from the two-way analysis of variance of BE detection rate on stratum and study arm.

Sensitivity analyses will aim at substituting the empirical between-cluster variance with the following predefined values of ICC: 0.025, lower and upper bound of 50% confidence interval of empirical ICC.

<u>Permutation test (on cluster-level summaries)</u>

To check the validity of our inferences, we will use a permutation test. See Sections 6.2.1 and 10.6.3 of Hayes and Moulton on Restricted Randomisation and Permutation Test.

The stratified design of the CLR group implies that restricted randomisation was used in assigning each cluster to its study arm. Let *N* be the total number of clusters, *M* be the number of strata and $\{m_i \mid i = 1, \ldots, M\}$ the size of the strata, so that $\sum_{i=1}^{M} m_i = N$. Then, if we require an equal number of clusters in the two study arms within each stratum, the number of possible allocations is:

$$\frac{m_1!}{\left(\frac{m_1}{2}\right)!\left(\frac{m_1}{2}\right)!} \times \ldots \times \frac{m_M!}{\left(\frac{m_M}{2}\right)!\left(\frac{m_M}{2}\right)!}$$

assuming the number of clusters per stratum is even.

According to the strata chosen after the Milestone 1 review and the number and size of practices as of November 2018, this number should be roughly equal to $3\text{x}10^{19}$, which is too large for the test to be computationally feasible (in a reasonable time). We will instead select a random sample of 5000 permutations. For each permutation, a t-test comparing BE detection rates between study arms will be performed. If the null hypothesis is true, then the observed effect measure can be regarded as having been randomly selected from this permutation distribution.

In Stata, this is done using the `permute` command.

PLR group

We will first report on the number of sites and patients per study arm, and average site size.

We will report on the weighted average follow-up for the PLR group as shown in Section 2.5.4.

Once again, the cumulative rate of BE diagnoses at 12 months will be estimated by dividing the follow-up into two periods: the first four months, and the subsequent follow-up (up to a maximum of 18 months).

A Poisson regression with BE detection rates / 1000 person-years as the outcome, study arm as the exposure and number of person-years as the offset will be run. The resulting rate ratio will be reported with 95% confidence interval.

Combined analysis (CLR + PLR group)

For the purposes of this analysis, the whole dataset will be considered. The same analysis as for the CLR group will be repeated (Poisson regression with random effects on individual-level data, stratified t-test on cluster-level data and permutation test), with the difference that the PLR group will represent a separate stratum of two clusters: one for patients randomised to the intervention and one for patients randomised to usual care. Note that the VIF for this cluster will be equal to 1 as we assume the ICC to be equal to 0: an ICC of 0 implies that there is no clustering so that individuals within the same cluster are no more similar than individuals from different clusters.

A weighted average follow-up will be calculated for the whole dataset as shown in Section 2.5.4. A further estimate of this will be made by considering only 6764 participants (in equivalent size terms) and we will check that this estimate is greater or equal than 12 months.

7.1.1.2 Adjusted analysis

Baseline data on age groups by sex, and length and dosage of acid suppressant medications are only available at practice level. The aggregated nature of the covariate data causes issues for the adjusted analysis both at individual level and at cluster level. For the latter, this is because adjustments for covariates are carried out with a two-stage procedure (see Section 12.3.2 of Hayes and Moulton) that, at first, relies on a regression model with individual-level data. Moreover, the aggregation of baseline data in PLR sites makes it impossible to separate intervention patients from usual care ones. Therefore, any adjusted analyses will not be possible for the primary endpoint.

7.1.1.3 Sensitivity analyses

As mentioned in Section 2.3, the primary endpoint analysis will be reiterated including also actual data on BE diagnoses in the intervention arm deriving from confirmatory trial endoscopies.

A further sensitivity analysis will impute possible additional cases of BE had all three data collection methods been used for all participants.

Moreover, in Section 3.2.2, we explained that three more sensitivity analyses will performed on the per-protocol population to control for the fact that a number of patients have less than 6 months' worth of acid-suppressant medication prescriptions in the year preceding baseline.

7.1.2 Analysis of secondary endpoints

For the sake of simplicity, the cluster design of part of the Trial will be ignored for the analysis of secondary endpoints.

Any endpoints using data on BE diagnoses will rely on *actual* data available from the Trial, i.e. the methods used for data collection for the primary endpoint will not apply.

The analysis of the secondary endpoints will be further detailed in a separate supplementary SAP.

<u>Using data from the intervention arm *only*:</u>

1) Diagnostic accuracy of the Cytosponge™ according to endoscopic findings:

   PPVs will be presented with 95% Clopper-Pearson (exact) confidence intervals overall, by age group/sex, by duration of acid-suppressant medication prescriptions prior to baseline and by number of columnal cells present on the sponge.

   In Stata, these can be calculated using the command `diagt` or `diagti`. In R, the function `epi.tests` should be used.

2) Diagnostic accuracy of the Cytosponge™ test according to endoscopic and pathology findings, i.e. by score of BE severity:

   PPV will be presented with a 95% exact confidence interval.

3) Performance of Cytosponge™ in detecting IM of the gastric cardia:

   PPV will be presented with a 95% exact confidence interval.

4) Performance of Cytosponge™ in detecting BE or IM of the gastric cardia:

   PPV will be presented with a 95% exact confidence interval.

5) Performance of Cytosponge™ in detecting EAC and gastric cancer:

   PPV will be presented with a 95% exact confidence interval;
   Numbers needed to examine (by Cytosponge™) to detect one OAC or one HGDB will also be calculated.

6) Sampling adequacy:

   Inadequacy rate will be presented with a 95% exact confidence interval.

7) Endoscopy referral rates for adequate test results and successful Cytosponge™ swallows:

   The two proportions will be presented with a 95% exact confidence interval.

8) Patient acceptability of Cytosponge™:

   Proportions will be presented with a 95% exact confidence interval.

   Median number of attempts to swallow per patient will be presented with interquartile range (IQR) and range.

   At baseline:

   - Measures will be presented with median, IQR and range

   At day 7-14:

   - Measures will be presented with median, IQR and range
   - Differences in STAI-6 scores at day 7-14 and baseline will be compared with a Wilcoxon signed-rank test.

9) Physician/nurse acceptability of Cytosponge™: qualitative outcome.

10) Safety of Cytosponge™:

    See Section 8.

11) Performance of repeat Cytosponge™ test:

Measures will be presented with a 95% exact confidence interval.

To be assessed using data from the usual care or *both* study arms:

12) Number of BE diagnoses missed in current management:

It will be estimated according to the following method.
Denote by *B* the number of cases of BE found and by *N* the numbers of participants in the denominator:
B_0 and N_0 refer to the numbers in the control arm (excluding the exit research endoscopies)
B_{01} and N_{01} refer to the numbers on the research endoscopies in the control arm
B_{11} and N_{11} refer to the numbers on the research endoscopies in the intervention arm (all Cytosponge™ negative at entry)
B_{10} and N_{10} refer to the numbers who did not have a Cytosponge™ in the intervention arm
N_{12} had a Cytosponge™ in the intervention arm with B_{12} BEs (excluding research endoscopies)
N_{13} had a positive Cytosponge™ and N_{14} had a subsequent endoscopy. B_{14} had BE found via that endoscopy. B_{15} had BE found subsequent to the endoscopy (i.e. after a "negative" endoscopy). B_{16} had BE found following a positive Cytosponge™ despite not having endoscopy as a result of that positive.
The number of BE cases found by usual care is B_0.
Among the N_{01} with a research endoscopy in the control arm, B_{01} cases of BE were missed under the current management. We need to calculate how many were missed in the $N_0 - N_{01}$ participants in the usual care arm without a research endoscopy.
First, consider how many BEs should have been found in the intervention arm had everyone been fully evaluated.
Had everyone with a positive Cytosponge™ had endoscopy, we estimate that $\frac{B_{14}}{N_{14}}N_{13}$ cases would have been found initially and $\frac{B_{15}}{N_{14}}N_{13}$ subsequently. The estimated total number of BE cases in those with a positive Cytosponge™ is:

$$T_1 = \frac{B_{14} + B_{15}}{N_{14}}N_{13} - B_{16}$$

The number of cases missed by Cytosponge™ could be estimated directly:

$$\frac{B_{11}}{N_{11}}(N_{12} - N_{13}).$$

But because N_{11} is (relatively) small, this number will be unstable. Instead, we will use the sensitivity of the Cytosponge™ from BEST2 (80%) in those who did not have a research endoscopy. We then estimate a total of:

$$B_{11} + 0.25(B_{14} + B_{15})\frac{N_{13}}{N_{14}} \times \frac{N_{12} - N_{13} - N_{11}}{N_{12} - N_{13}}$$

missed cases among those with a negative Cytosponge™. The estimated total number of cases in those with a negative Cytosponge™ is:

$$T_2 = (B_{12} - B_{14} - B_{15} - B_{16}) + B_{11} + 0.25T_1\frac{N_{12} - N_{13} - N_{11}}{N_{12} - N_{13}}$$

Next, we need to consider how many cases would have been found in those having a Cytosponge™ had they been in the usual care arm. We

assume that those accepting a Cytosponge™ may not have the same rate as in those that did not accept. By subtraction, we estimate

$$T_3 = \frac{B_0}{N_0}(N_{10} + N_{12}) - B_{10}$$

cases among people accepting a Cytosponge™ (had they not been offered a Cytosponge™). So, among those using a Cytosponge™, BE was increased by the factor: $\frac{T_1+T_2}{T_3}$.

As in the intervention arm, we do not simply scale up from the research endoscopies in the control arm. Rather we combine the cases observed directly among those with a research endoscopy, by the expected number amongst the others using the intervention arm to scale up. The scale factor needed $R_0 = \frac{N_0 - N_{01}}{N_{10}+N_{12}}$, i.e. the numbers of people who did not get a research endoscopy in the control arm, divided by the total number in the intervention arm. The total number of cases in the intervention arm is made up of three parts: those observed by research endoscopy plus those in people who would have accepted the Cytosponge™, plus those among people who would not have accepted the Cytosponge™ is offered. The total is estimated as:

$$T_4 = B_{01} + (T_1 + T_2)R + \frac{T_1 + T_2}{T_3} B_{10} R$$

Hence the proportion of BE missed by current management is $\frac{T_4 - B_0}{T_4}$.

13) Number of undiagnosed BE in the general population vs in the group who received Cytosponge™:

The number of undiagnosed BEs in the patients who received the Cytosponge™ will be estimated by multiplying the proportion of BEs detected following a research endoscopy in the negative test group by the number of negative test patients.

The number of undiagnosed BEs in the usual care arm will be estimated by multiplying the proportion of BEs following a research endoscopy in the usual care arm by the number of patients in the usual care arm.

The two proportions of undiagnosed BEs will then be calculated out of the total number of patients in each of the two groups and will be compared using a chi-squared test.

14) Acceptability of endoscopy:

The proportion of participants in the usual care arm who attend their research endoscopy invitation will be compared to the proportion of participants in the intervention arm who attend their Cytosponge™ invitation using a chi-squared test.

15) Number of BE diagnoses for patients with a negative Cytosponge™ result:

Number of false negatives of the test arising from research endoscopies will be used to estimate the false omission rate, where we will use as denominator the number of negative Cytosponge™ patients who attend a research endoscopy invitation. The False Omission Rate will be reported with 95% confidence interval.

16) Quality control of endoscopic and pathology results:

For participants swallowing the Cytosponge™ successfully:

- Number (%) of BE diagnoses missed
- Number (%) of any other malignant diagnoses missed
- Number (%) of BEs falsely detected (if any)
- A “true” PPV for BE will be calculated and presented with 95% confidence interval.

For research endoscopies, usual care arm and negative Cytosponge™ patients separately:

- Number (%) of BE diagnoses missed
- Number (%) of any other malignant diagnoses missed
- Number (%) of BEs falsely detected (if any).

7.1.3 Analysis of further subgroups

Because of the nature of the data, we only have individual-level information available for Cytosponge™ patients. Exploratory analyses may be performed by subgroup created using data gathered during the Cytosponge™ appointment, such as age group, gender, BMI, smoking history, etc.

7.2 Covariates

No covariates will be introduced in the primary endpoint analysis because of the type of analysis and the structure of the data available (see Section 7.1.1.2). It should be noted, however, that the primary endpoint analysis will be performed by period (up to 4 months vs from 4 to 18 months).

7.3 Methods for handling missed data and outliers

Any outliers found in the data will be checked with the study sites when possible. Otherwise, they will be substituted by empty fields.

7.3.1 Handling of dropouts

All study subjects received a letter before their follow-up began to inform them about the use of their data within the Trial and to give them the option of opting out of it before 14 days. However, in a handful of cases, participants withdrew consent to the study after the 14-day period (or the practice alerted the trials team late about the objection) and their records were consequently deleted from the Trial database. File notes were filled in for each one of these withdrawals.

As intervention subjects received further letters inviting them to the Cytosponge™ test, it is more likely that they will have withdrawn of the Trial after the 14-day opt-out period in a higher number than usual care patients. For a similar argument, intervention practices in the CLR group, who were more involved in the trial, were more likely to report to the trials team any late opt-outs than usual care practices.

It should also be noted that, in the PLR group, a handful of patients also opted out after being randomised.

Number of dropouts will be reported on, but they will be excluded from any endpoint analysis. However, because of the aggregated (i.e. site-level) nature of the data on age groups and medications, we will not be able to exclude these patients from any summary statistics on these baseline characteristics. A further sensitivity analysis will see dropouts not excluded and treated as participants without BE.

7.3.2 Handling of missing data in active subjects

We do not expect to see any missing data for any primary or secondary endpoints, except for those outcomes linked to patient acceptability questionnaires, for any

outcomes measured in patients who died or moved away during the trial, and for any sites not performing any manual case-note reviews of their records at the end of follow-up.

As we expect very low percentages of missing data, when dealing with missing values for an endpoint analysis, we will exclude individual records accordingly..

8. Safety analysis

8.1 Summary of adverse events

All of the following will be presented by participants producing a successful swallow at first test, participants producing a successful swallow at repeat test, overall (first and repeat test combined, with only AEs from the repeat test contributing for participants who had two tests) and in total (both first and repeat test counted as separate instances).

8.1.1 Number of adverse events

AEs up to 7 days after receiving the test for participants successfully swallowing the Cytosponge™ test will be presented:

- by type: number and distribution
- by severity (severe, moderate, mild): number and distribution
- by study site: number and distribution; median/range by site
- overall: total number

An example of this is available in the DMC report from October 2018:

G:\EMS\CPTU\BEST3\Section 17 TRIAL COMMITTEES\Data Monitoring Committee (DMC)\Meetings (agenda and minutes)\4. DMC meeting - 30 October 2018\BEST3 DMC Report - Open - 30 October 2018.pdf

8.1.2 Number of patients affected by an adverse event

We will report on total number of patients affected by an AE up to 7 days after receiving the test and their proportion over the number of patients who swallowed a Cytosponge™ successfully.

As patients can experience more than one AE, we will also show the median number and range of AEs per participant.

Number and distribution of patients affected by AEs will be presented by site. Median/range by site should also be presented.

8.2 Analysis of adverse events

No statistical analysis of AEs is planned due to the fact that there is no comparison between study arms. However, we may choose to report some of the figures on AEs by subgroup, such as age group, gender, BMI, smoking history, etc.

8.3 Summary of Serious Adverse Events (SAE)

As for the above, SAEs will only be listed for responders of the Cytosponge™. They will be presented individually stating the participant ID, the event narrative, and the relationship with having undertaken the Cytosponge™.

An example of this is available in the DMC report from October 2018:

G:\EMS\CPTU\BEST3\Section 17 TRIAL COMMITTEES\Data Monitoring Committee (DMC)\Meetings (agenda and minutes)\4. DMC meeting - 30 October 2018\BEST3 DMC Report - Open - 30 October 2018.pdf

Number/proportion of SAE (out of all successful swallows) will be reported.

8.4 Analysis of SAE

SAEs are expected to be a rare occurrence in the Trial, so no statistical analysis is planned.

9. Presentation of analysis

Two statisticians will work on the statistical analysis to ensure its reliability: one will write the code, the other one will review it.

9.1 Reporting of results

A statistical report will be prepared, which will follow loosely the following structure:

- CONSORT diagram
- Power calculations
- Check on weighted average follow-up
- Patients' demographics summary (for groups/individuals for which these are available)
- Primary endpoint
- Secondary endpoints
- AEs
- Protocol deviations/violations

The CONSORT diagram will be prepared expanding on the Trial flowchart below (temporary figures as of July 2019). The following numbers will be added to the diagram:

- Number of sites who opted out *after* randomisation (CLR group only)
- Patients who opted out *after* randomisation (PLR group only)
- Patients excluded from analysis

Labels for "Enrolment", "Allocation" and "Analysis" will also be added.

Figure 2. Trial flowchart – as of July 2019

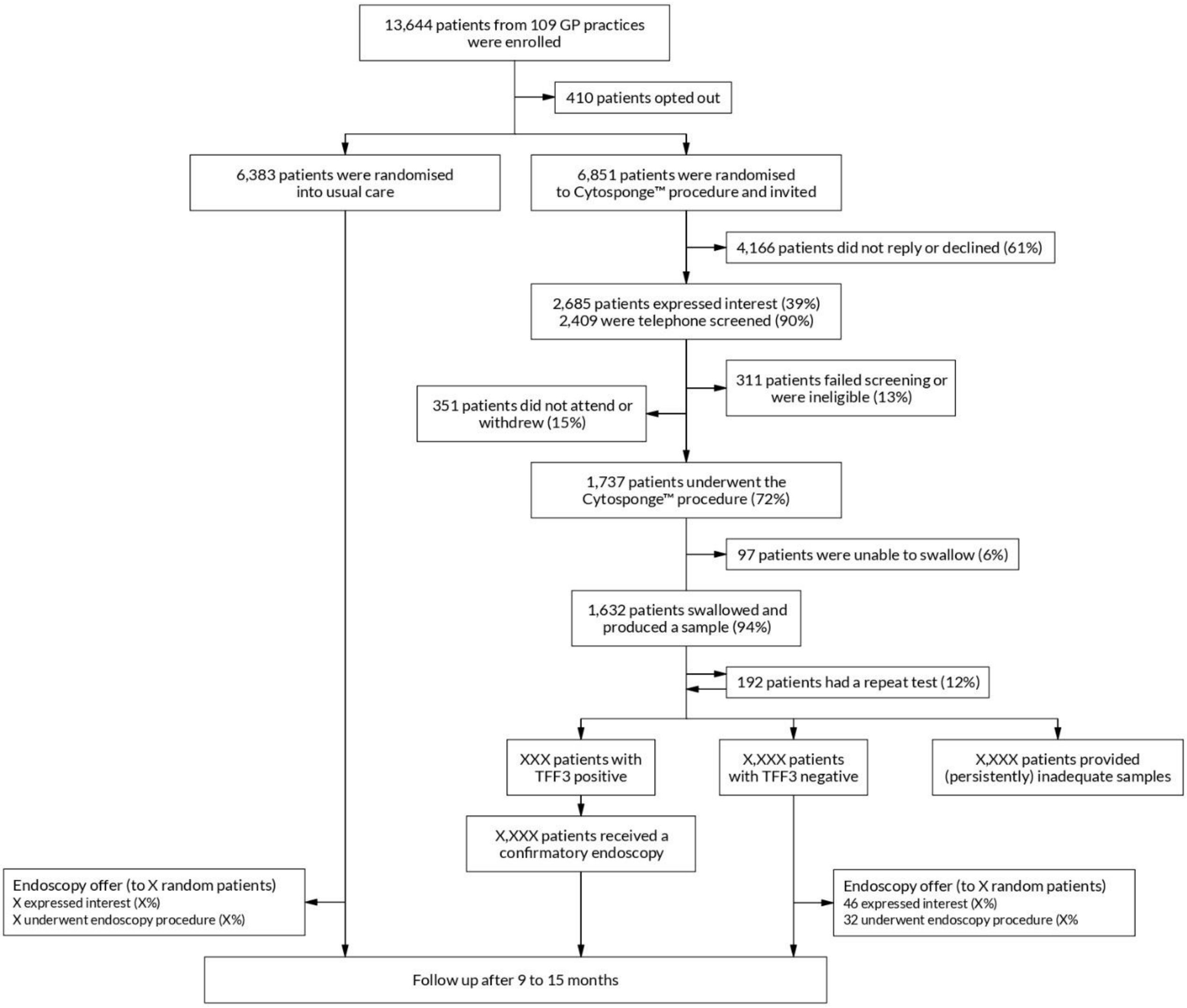

9.2 Presentation of results

A statistical report will be prepared. Results will be discussed in a meeting with the study team.

One or more publications will follow.

10. References, related SOPs, web links

SOP Barts CTU GEN ST 01 “Statistical Analysis Plan”, version 4.0

BEST3 Epidemiological Analysis Plan

BEST3 Health Economics Analysis Plan

Randomisation SOP, validation and list: G:\EMS\CPTU\BEST3\Section 9 REGISTRATION AND RANDOMISATION

Cuzick J, Edwards R, Segnan N. Adjusting for non-compliance and contamination in randomized clinical trials. Stat Med. 1997 May 15;16(9):1017-29. Erratum in: Stat Med. 2007 Sep 10;26(20):3821.

Hayes RJ and Moulton LH. (2009). Cluster randomised trials. ed. Boca Raton, FL: Chapman & Hall/CRC, pp. 178-9

StataCorp. 2017. *Stata Statistical Software: Release 15*. College Station, TX: StataCorp LLC

R Core Team (2019). R: A language and environment for statistical computing. R Foundation for Statistical Computing, Vienna, Austria. URL https://www.R-project.org/.

RStudio Team (2018). RStudio: Integrated Development for R. RStudio, Inc., Boston, MA URL http://www.rstudio.com/

11. Appendices and associated documents

Study protocol: G:\EMS\CPTU\BEST3\Section 4 PROTOCOL

Protocol amendments: G:\EMS\CPTU\BEST3\Section 6 APPROVALS AND AUTHORISATIONS\8. Amendments

DMC meeting reports: G:\EMS\CPTU\BEST3\Section 17 TRIAL COMMITTEES\Data Monitoring Committee (DMC)\Meetings (agenda and minutes)

Sample size calculations: G:\EMS\CPTU\BEST3\Section 26 STATISTICS\26.3 Power calculations\Sample size (following Amendment 6)\BEST3_Sample size.pdf

Communication from MHRA, Your Ref: Amendment 8 (REC Amendment 6), 31 May 2018: G:\EMS\CPTU\BEST3\Section 26 STATISTICS\26.4 Statistical analysis plan\References\Amendment [7] Final Decision Letter.pdf

CRFs: G:\EMS\CPTU\BEST3\Section 10 CASE REPORT FORM (CRF)\10.1 Current version\BEST3 eCRFs

Appendix 5 – Supplementary figures and tables

Figure S1 – CONSORT diagram for the cluster randomised group.

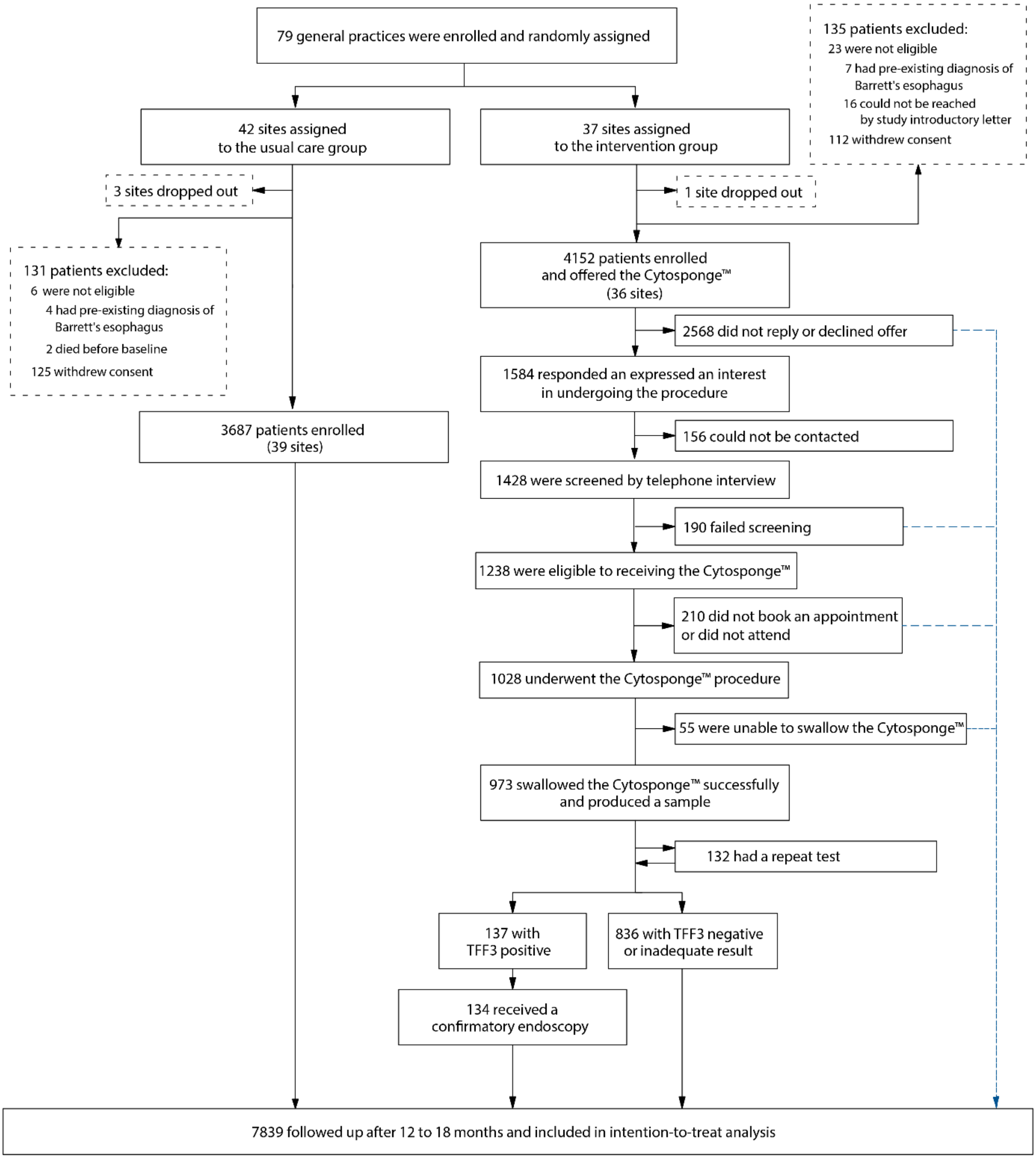

Figure S2 – CONSORT diagram for the individual randomised group.

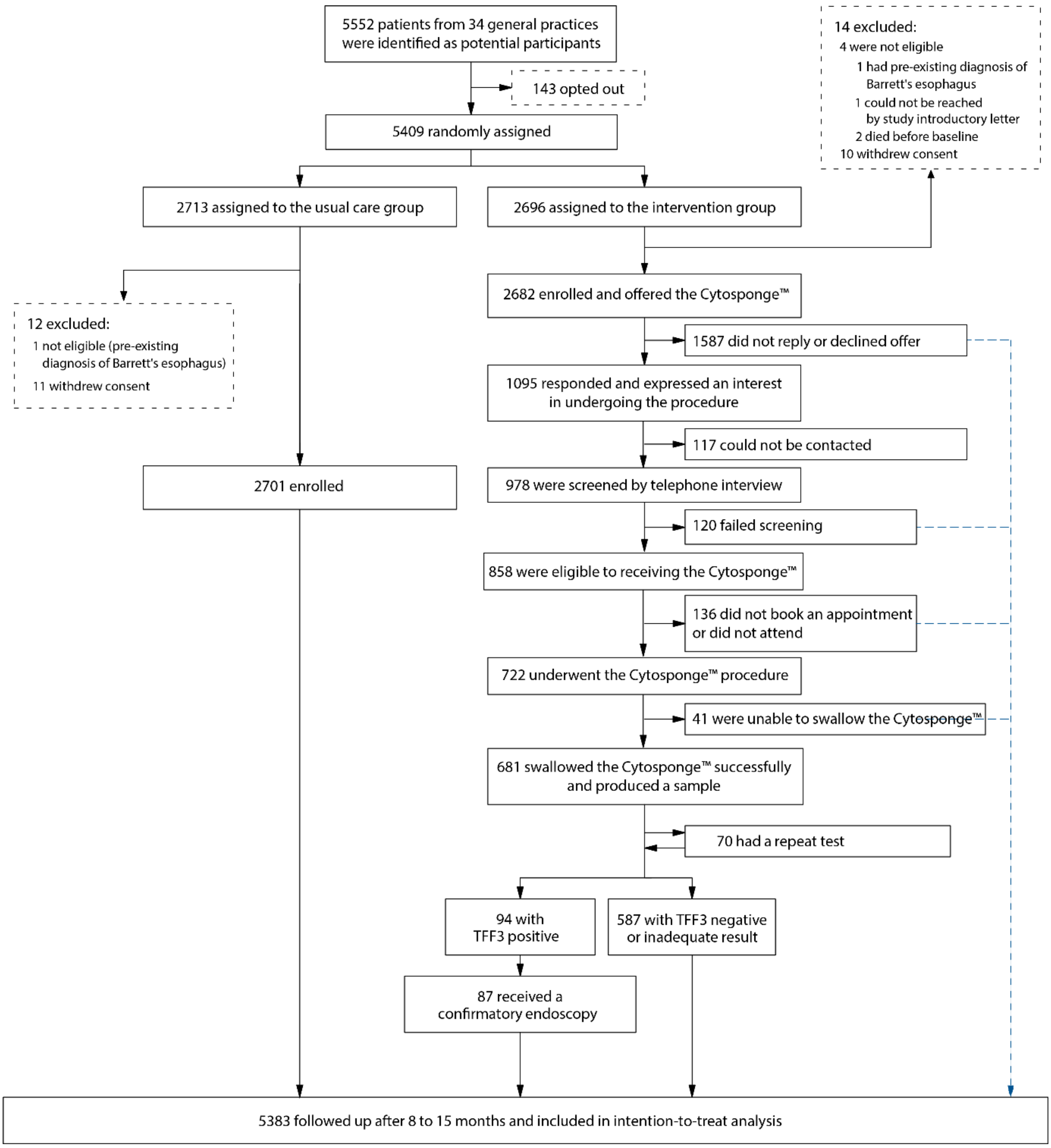

Table S1. List of BEST3 clinics and their postcodes.

Acorn	PE29 7HN	Hampstead Medical Practice	NW3 2QU
Adam Practice	BH15 2HX	Hoveton	NR12 8DU
Abbey Field Medical Centre	CO2 7UW	Haxby Surgery	YO32 2LL
St. Andrew’s Medical Practice	DL16 6QA	Bicester	OX26 6AT
Boroughbury Medical Centre	PE1 2EJ	Imeary Street Practice	NE33 4EG
Beccles Medical Practice	NR34 9NX	Ixworth Surgery	IP31 2HD
Bury Road Surgery	PO12 3PW	Keats Group Practice	NW3 1NR
Beacon Medical Group	PL7 2QP	Leadgate Surgery	DH8 6DP
Bishopgate Medical Centre	DL14 7EJ	Liphook & Liss Surgery	GU33 7LE
Broadshires Medical Practice	OX18 1JA	Little St John's	IP12 1EE
Buckden	PE19 5SZ	Lawson Road Surgery	NR3 4LE
Bottisham Surgery	CB25 9DU	Manor Farm - Swaffham	PE37 7QN
Bridge Street Medical Centre	CB2 3LS	Magdalen Medical Practice	NR3 4LF
Bretton Medical Practice	PE3 8DT	Millfield Surgery	YO61 3JR
Burwell Surgery	CB25 0AE	Market Surgery	NR11 6BW
Bungay Medical Practice	NR35 1LP	MyHealth Practice	YO32 5UA
Comberton Surgery	CB23 7DY	New Queen St	PE7 1AT
Cedar House Surgery	PE19 1BQ	Orchard Surgery	NR19 1AE
Cherry Hinton	CB1 9HR	Oaks Medical Centre	NG9 2NY
Clanfield Surgery	PO8 0QL	Orchard House Surgery	CB8 8NU
Cathedral	CB6 1DN	The Peninsula Practice	IP12 3DA
Lower Clapton Group Practice	E5 0PQ	Pickering Practice	YO18 8BL
Colchester Medical Practice	CO3 4RY	Pelton Surgery	DH2 1HS
Castle Partnership	NR4 7QX	Portmill, Hitchin	SG4 9TH
Church St	OX12 9BN	Posterngate Surgery	YO8 4QH
Cornerstone	PE15 9BF	Priory Fields	PE29 3RL
Cottenham Medical Practice	CB24 8SE	Parsonage Surgery	CM23 5JH
Chesterfield Drive	IP1 6DW	Papworth Surgery	CB23 3QQ
Cowplain Family Practice Site	PO8 8DL	Quarterjack Surgery	BH21 1AP
Claypath Medical Group	DH1 1QW	Queens Road Surgery	DH8 0BW
Bridge Rd, Lowestoft	NR32 3LJ	Roborough	PL6 6PH
The Denmead Practice	PO7 6NR	Rosedale Surgery	NR33 8LG
Derby Rd, Nottingham	NG7 2DW	Riverside	PE15 8BG
East Norfolk Medical Practice	NR30 1QP	South Oxford Health Centre	OX1 4RP
Eynsham Medical Practice	OX29 4QB	Southgates Medical & Surgi-cal Centre	PE30 5QX
Fakenham Medical Practice	NR21 8SY	Sheringham Medical Practice	NR26 8RT
Doddington	PE15 0TG	The Spinney Surgery	PE27 3TP
Flitwick Surgery	MK45 1DW	Skerne Medical Group	TS21 3BN
Great Bentley Surgery	CO7 8PJ	Shelford Medical Practice	CB22 5FY
Gosford Hill Medical Practice	OX5 2NS	St Mary's Surgery	CB7 4HF
Gt Massingham/Docking	PE32 2JQ	Staploe Medical Centre	CB7 5JD
Granta Medical Practice	CB22 3HU	St Stephen's Gate	NR2 2TJ
Grove Surgery	IP24 2HY	Staithe Surgery	NR12 9BU
Great Lumley Surgery	DH3 4LE	Summertown Health Centre	OX2 7BS
The Health Centre Practice	SG8 7BS	Swan Surgery	IP33 1AE
Stowhealth, Suffolk	IP14 1NL	Tollerton Surgery	YO61 1QW

Hingham Surgery	NR9 4JB
Homewell.Curlew Practice	PO9 2AQ
Hampstead Medical Practice	NW3 2QU
Hoveton	NR12 8DU
Haxby Surgery	YO32 2LL
Bicester	OX26 6AT
Imeary Street Practice	NE33 4EG
Ixworth Surgery	IP31 2HD
Keats Group Practice	NW3 1NR
Leadgate Surgery	DH8 6DP
Liphook & Liss Surgery	GU33 7LE
Little St John's	IP12 1EE
Lawson Road Surgery	NR3 4LE
Manor Farm - Swaffham	PE37 7QN
Magdalen Medical Practice	NR3 4LF
Millfield Surgery	YO61 3JR
Market Surgery	NR11 6BW
MyHealth Practice	YO32 5UA
New Queen St	PE7 1AT
Orchard Surgery	NR19 1AE
Oaks Medical Centre	NG9 2NY
Orchard House Surgery	CB8 8NU
The Peninsula Practice	IP12 3DA
Pickering Practice	YO18 8BL
Pelton Surgery	DH2 1HS
Portmill, Hitchin	SG4 9TH
Posterngate Surgery	YO8 4QH
Priory Fields	PE29 3RL
Parsonage Surgery	CM23 5JH
Papworth Surgery	CB23 3QQ
Quarterjack Surgery	BH21 1AP
Queens Road Surgery	DH8 0BW
Roborough	PL6 6PH
Rosedale Surgery	NR33 8LG
Riverside	PE15 8BG
South Oxford Health Centre	OX1 4RP
Southgates Medical & Surgical Centre	PE30 5QX
Sheringham Medical Practice	NR26 8RT
The Spinney Surgery	PE27 3TP
Skerne Medical Group	TS21 3BN
Shelford Medical Practice	CB22 5FY
St Mary's Surgery	CB7 4HF
Staploe Medical Centre	CB7 5JD
St Stephen's Gate	NR2 2TJ
Staithe Surgery	NR12 9BU
Summertown Health Centre	OX2 7BS
Swan Surgery	IP33 1AE
Trafalgar Medical Group Practice	PO5 3ND
Tollerton Surgery	YO61 1QW
Trafalgar Medical Group Practice	PO5 3ND
Tavyside Health Centre	PL19 9FD
Vida Healthcare - Carole Brown Surgery	PE36 5DN
Victoria Medical Centre	NE31 1NU
Vida Healthcare	PE31 6GZ
Vine Medical Practice	PO7 7AH
Woodlands	TS18 1YE
Westlands Medical Practice	PO16 9AD
Wansford and Kings Cliffe	PE8 6PL
Whiteley Surgery	PO15 7LB
Wickham Market Medical Centre	IP13 0SB
Woolpit Health Centre	IP30 9QU
Wellside	PE28 5SU
White Horse Medical Practice	SN7 7YP
Yarm	TS15 9DD

Table S2. Barrett's oesophagus diagnoses in the usual care group compared with the intervention group, cluster-randomised group only.

	Usual care group (n = 3687)	Intervention group (n = 4152)	Absolute difference (95% CI)	Overall rate ratio (95% CI)	Overall adjusted rate ratio* (95% CI); p-value
Number of participants diagnosed with Barrett's oesophagus	9 (0.2%)	92 (2.2%)†	-	-	-
Follow-up, person-years	4,006	4,421	-	-	-
Incidence of Barrett's oesophagus, per 1000 person-years	2·2	21·2‡	18·9 (16·8-21·0)	9·4 (4·8-18·7)	10·0 (5·0-20·0); p < 0·0001

Data are n (%), unless otherwise specified.
*Overall adjusted rate ratio accounts for cluster-level randomisation
†Number of participants diagnosed with Barrett's oesophagus in the intervention group includes all participants who were offered the Cytosponge procedure.
‡The incidence of Barrett's oesophagus in the intervention group was calculated as the weighted average of the incidence in the first 4 months of follow-up and the incidence in the following months, with a weight ratio of 1:2

Table S3. Barrett's oesophagus diagnoses in the usual care group compared with the intervention group, individual randomised group only.

	Usual care group (n = 2701)	Intervention group (n = 2682)	Absolute difference (95% CI)	Overall rate ratio (95% CI); p-value
Number of participants diagnosed with Barrett's oesophagus	4 (0.1%)	48 (1.8%)†	-	-
Follow-up, person-years	2,573	2,531	-	-
Incidence of Barrett's oesophagus, per 1000 person-years	1·6	18·6‡	17·1 (11·5-22·6)	12·0 (4·3-33·2); p < 0·0001

Data are n (%), unless otherwise specified.
†Number of participants diagnosed with Barrett's oesophagus in the intervention group includes all participants who were offered the Cytosponge procedure.
‡The incidence of Barrett's oesophagus in the intervention group was calculated as the weighted average of the incidence in the first 4 months of follow-up and the incidence in the following months, with a weight ratio of 1:2

Table S4. Length of Barrett's oesophagus in cm (Maximal length (M) from Prague CM Classification) across the study arms.

	Usual care group (n = 6388)	Intervention group		
		Underwent the Cytosponge procedure (n = 1750)	Did not undergo the Cytosponge procedure (n = 5084)	Overall (n = 6834)
< 1cm	1 (8%)	2	0	2 (1%)
1 to <2 cm	3 (23%)	41	3	44 (31%)
2 to <3 cm	3 (23%)	21	4	25 (18%)
3 to <4 cm	1 (8%)	14	1	15 (11%)
4 to <5 cm	0	8	1	9 (6%)
5 to <6 cm	1 (8%)	9	1	10 (7%)
6 to <7 cm	1 (8%)	2	0	2 (1%)
7 to <8 cm	0	3	0	3 (2%)
8+ cm	1 (8%)	6	1	7 (5%)
missing	2 (15%)	21	2	23 (16%)
**Total number of participants with Barrett's oesophagus**	**13 (100%)**	**127**	**13**	**140 (100%)**

Data are n (%). Only coded Barrett's oesophagus diagnoses, i.e. used for the intention-to-treat primary endpoint analysis, are shown.

Table S5. Stage and treatment for dysplasia and cancer cases across all study arms.

Case by case	TNM stage	Overall stage	Treatment
Usual care group (n = 6388)	T3N0MX	Stage IIB	Robotic-assisted esophagectomy
	T3N2M0	Stage IIIB	Palliative radiotherapy + stent<br>RIP
	T3N3M1	Stage IVB	Best supportive care + stent<br>RIP 1 month post diagnosis
Intervention group – Underwent the Cytosponge procedure (n = 1750)	LGD	Dysplasia	APC
	LGD-HGD	Dysplasia	RFA
	LGD-HGD	Dysplasia	RFA
	HGD	Dysplasia	EMR
	T1N0MX	Stage I	EMR
	T1bN0M0	Stage I	Oesophagectomy
	SM1 OAC	Stage I	EMR
	T1N0M0 (Gastric on background extensive IM)	Stage I	ESD
Intervention group – Did not undergo the Cytosponge procedure (n = 5084)	T1N0M0	Stage I	EMR, RFA and APC<br>(Patient initially expressed interest in receiving the Cytosponge)
	T3N2M0	Stage IVA	Palliative chemotherapy
	T3N2M1b	Stage IVB	Best supportive care<br>RIP 3 months post diagnosis

RIP = Rest in peace
APC = Argon plasma coagulation
RFA = Radiofrequency ablation
EMR = Endoscopic mucosal resection
ESD = Endoscopic submucosal dissection

Supplementary Table S6. Cytosponge-TFF3 acceptability scores.

Acceptability score*	Participants who successfully swallowed the Cytosponge (n = 1654)
0	1 (<0·1%)
1	2 (0·1%)
2	5 (0·3%)
3	13 (0·9%)
4	16 (1·1%)
5	92 (6·2%)
6	63 (4·3%)
7	103 (7·0%)
8	247 (16·9%)
9	317 (21·7%)
10	605 (41·3%)
**Total number of patients filling in the questionnaire**	**1464 (100·0%)**

Data are n (%).
*11-point visual analogue scale: 0 = unacceptable, 10 = completely acceptable.
